# Tunable Gold‐catalyzed Reactions of Propargyl Alcohols and Aryl Nucleophiles

**DOI:** 10.1002/open.202200030

**Published:** 2022-03-10

**Authors:** Helgi Freyr Jónsson, Thomas Nordbø Solvi, Sondre Lomeland, Ann Christin Reiersølmoen, Anne Fiksdahl

**Affiliations:** ^1^ Department of Chemistry Norwegian University of Science and Technology Høgskoleringen 5 7491 Trondheim Norway

**Keywords:** allene, aryl nucleophiles, gold catalysis, propargyl alcohol, tunable conditions

## Abstract

Gold‐catalyzed transformations of 1,3‐diarylpropargyl alcohols and various aryl nucleophiles were studied. Selective tunable synthetic methods were developed for 1,1,3‐triarylallenes, diaryl‐indenes and tetraaryl‐allyl target products by C3 nucleophilic substitution and subsequent intra‐ or intermolecular hydroarylation, respectively. The reactions were scoped with regards to gold(I)/(III) catalysts, solvent, temperature, and electronic and steric effects of both the diarylpropargyl alcohol and the aryl nucleophiles. High yields of triaryl‐allenes and diaryl‐indenes by gold(III) catalysis were observed. Depending on the choice of aryl nucleophile and control of reaction temperature, different product ratios have been obtained. Alternatively, tetraaryl‐allyl target products were formed by a sequential one‐pot tandem process from appropriate propargyl substrates and two different aryl nucleophiles. Corresponding halo‐arylation products (I and Br; up to 95 % 2‐halo‐diaryl‐indenes) were obtained in a one‐pot manner in the presence of the respective *N*‐halosuccinimides (NIS, NBS).

## Graphical abstract

**Tunable gold(III)‐catalyzed transformations** of 1,3‐diarylpropargyl alcohols with aryl nucleophiles were studied. Highly selective and tunable synthetic methods were developed for one‐pot formation of either triaryl‐allenes, diaryl‐indenes or tetraaryl‐allyl target products by appropriate reaction conditions (time, temperature, catalyst, substrate, nucleophile, solvent). Corresponding 2‐halo‐indenes, appropriate for further Pd‐catalyzed reactions, were also obtained in a one‐pot manner in the presence of NXS (X=Br, I).

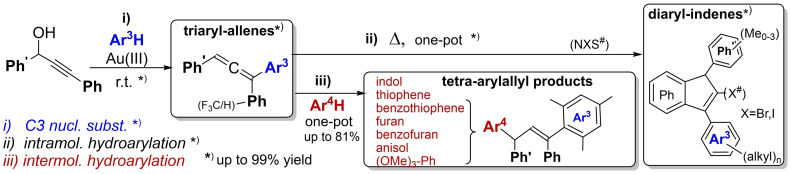

## Introduction

**Scheme 1:**
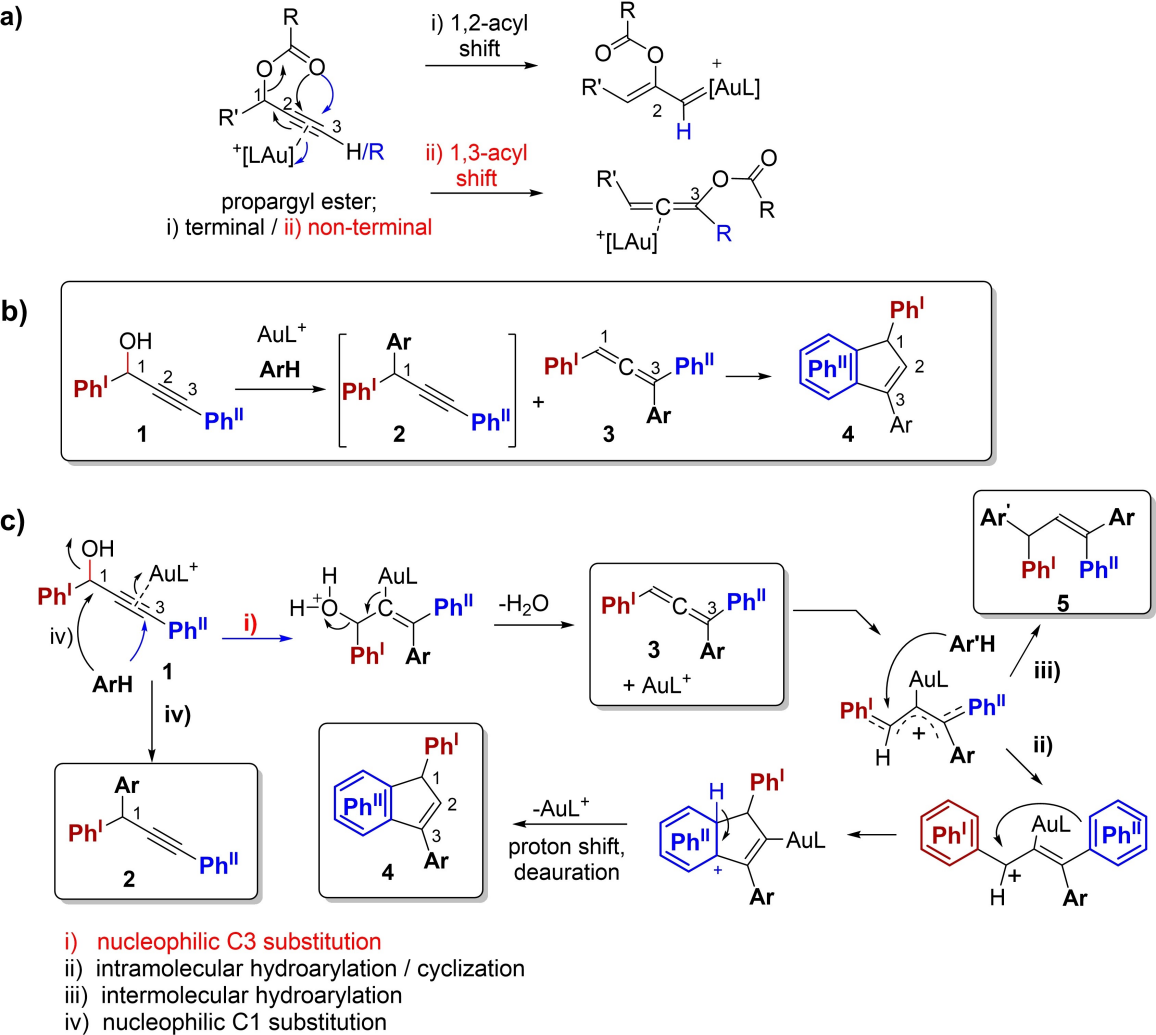
Gold‐catalyzed transformations of propargyl substrates. a) Reactivity patterns of propargyl esters. b) Reaction of propargyl alcohols **1** with aryl nucleophiles, ArH.^[30]^ c) Suggested mechanism for Au‐catalyzed formation of i) 1,1,3‐triarylsubstituted allenes^[27]^ **3**, ii) diaryl‐indenes^[27]^ **4**, iii) 1,1,3,3‐tetraaryl‐allyl products **5** and iv) propynes **2**, from propargyl alcohol **1** and aryl nucleophiles ArH/Ar'H.

Propargyl substrates represent a versatile group of substrates for a great variety of gold‐catalyzed transformations. Propargylic esters may undergo a series of inter‐ and intramolecular transformations, initiated by a gold‐catalyzed 1,2‐ or 1,3‐acyl shift (Scheme 1a), to give the respective gold carbenoid or allene species, which both may be prone for further transformations.[^1^, ^2^, ^3^, ^4^, ^5^, ^6^, ^7^] The Fiksdahl group has contributed to the progress of gold catalysis in organic synthesis by the development of a number of cycloaddition reactions based on the highly reactive terminal propargyl acetal substrates and a series of reactants through initial gold‐catalyzed 1,2‐alkoxy shifts.[^8^, ^9^, ^10^, ^11^, ^12^, ^13^, ^14^, ^15^, ^16^, ^17^, ^18^, ^19^, ^20^, ^21^, ^22^, ^23^, ^24^, ^25^, ^26^] Our studies demonstrate the particular potential and versatility of propargyl acetals in gold(I)‐catalyzed cycloaddition reactions.

The combination of a C‐1 alcohol leaving group in non‐terminal propargyl alcohols **1** (Scheme 1b) and an external protic aromatic nucleophile ArH allows for a similar allene reaction pathway as the 1,3‐acyl shift of propargyl esters (Scheme 1a–ii). Thus diaromatic (Ph^I^, Ph^II^) propargyl alcohols **1** and aryl nucleophiles are reported to give triaryl‐allenes **3** and indenes **4** by gold catalysis (Scheme 1b).^[27]^ The initial allenes **3**, formed by i) S_N_2’ nucleophilic aryl attack at C3, undergo subsequent ii) intramolecular hydroarylation by a Nazarov cyclisation‐like step[^28^, ^29^] by heating, to give indene product **4** (Scheme 1c). Whether heating assisted the Au‐allene interaction or the Nazarov cyclisation was not discussed. In general, the indene formation by ii) cyclisation (Scheme 1c) of the vinyl‐gold intermediate could potentially take place by incorporation of either of the two phenyl groups (Ph^I^ or Ph^II^ in Scheme 1) of allene **3**. Reaction with Ph^I^ would proceed via a doubly stabilized benzylic carbocation but would give a potential geminal di‐aryl‐substituted indene product. However, as proved by the obtained indene products **4**, steric effects and incorporation of Ph^II^ via the planar gold cationic mono‐benzylic intermediate, seemed to be dominant for successful reactions. An alternative iii) intermolecular hydroarylation pathway of allenes **3** with a second aromatic nucleophile Ar'H, affording 1,1,3,3‐tetraaryl‐allylic product **5**, could be envisioned, but has not previously been studied.^[30]^ Additionally, the competing direct iv) aryl C1 nucleophilic substitution on propargyl alcohols **1** would yield 1,1,3‐triarylpropargyl products (**2**) by gold catalysis,[^30^, ^31^, ^32^] also known with other transition metals or Lewis acids.[^33^, ^34^, ^35^, ^36^]

Allenes are important subunits in a variety of natural products and pharmaceutically related compounds,^[37]^ as well as versatile synthons in synthetic organic chemistry because of their ability to undergo a diversity of transformations in inter‐ or intramolecular fashion. Gold interacts with allenes, and may form stable, isolable complexes.^[38]^ Gold‐catalyzed transformations of allenes mainly involve cycloadditions or inter‐/intramolecular nucleophilic addition reactions,^[39]^ including hydroarylations.^[40]^ Thus, a variety of carbo‐ or heterocycles can be produced by allene cyclizations.^[41]^ The gold catalyst can coordinate to either allenic double bond, and the regioselectivity of the subsequent nucleophilic attack depends on the structure of both the allene substrate and the reactant, and different products may be formed. Hence, efficient and simple approaches for allene synthesis are important. In addition to the presently studied synthesis of allenes by gold‐catalyzed intermolecular reaction of benzylic propargylic alcohols and aryl nucleophiles,^[42]^ allenes are normally synthesized by prototropic rearrangement of the corresponding propyne,[^43^, ^44^] by sigmatropic rearrangements,[^45^, ^46^] as well as Cu^II^‐catalyzed coupling, additions to enynes, 1,2‐eliminations and Wittig‐type reactions.[^47^, ^48^]

The indene (1*H*‐indene) structure moiety (**4**) is an attractive scaffold due to its biological activities.[^49^, ^50^] Indene is a stable structure, resisting oxidation of the cyclopentene ring even by harsh conditions.^[51]^ Several metal‐catalyzed reactions[^2^, ^52^, ^53^, ^54^, ^55^] are used for synthesis of substituted indenes. The Au‐catalyzed indene cyclisation of propargyl acetates proceeds through the allene precursor and gives different indene regioisomers[^1^, ^56^] by 1,2‐ and 1,3‐acyl shift, followed by hydroarylation.^[56]^ This reaction is further developed with propargyl alcohols.^[30]^

The Fiksdahl research group is currently working on the development of novel Au^I^ and Au^III^ complexes. We have established a set of standardized Au‐catalyzed test‐reactions for screening and evaluation of the catalytic ability of new Au^I^ and Au^III^ complexes. The results demonstrate how the catalyst properties and stability are dependent on ligand structure, and the importance of ligand design. High catalytic activity has been proved in selected test reactions, such as propargyl based transformations.[^10^, ^11^, ^12^, ^13^, ^14^, ^15^, ^16^, ^21^, ^23^, ^24^, ^25^] As gold‐catalyzed transformations of nonterminal propargyl alcohols are not commonly reported, we wanted to investigate the potential of the reaction of propargyl alcohols **1** with aromatic nucleophile ArH (Scheme 1) in order to be included in our list of gold‐catalyzed model reactions. Based on the previous studies^[30]^ of the reaction, which applied a limited choice of Au catalysts and mainly focused on formation of the indene product **4**, we have carried out a more comprehensive study to look closer at reaction conditions and additional products. With the aim of understanding factors favoring selective synthesis of allenes **2**, indenes **4** and possible tetraaryl‐allylic **5** products, the reactions were scoped with regards to solvent, Au catalyst, electronic and steric effects of propargyl alcohol **1** and aryl nucleophiles.

## Results and Discussion

### Synthesis of Propargyl Alcohols

**Scheme 2:**
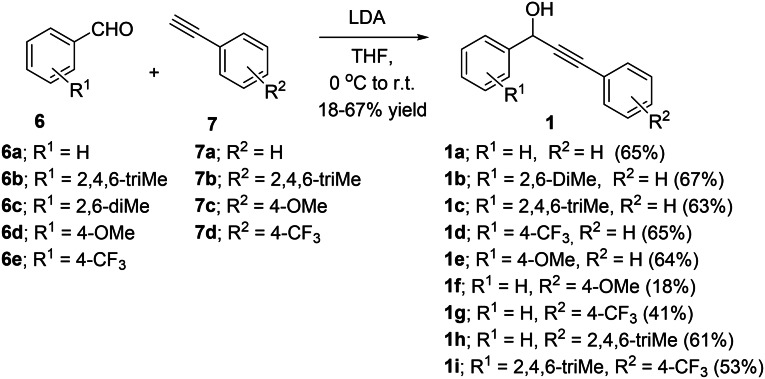
Synthesis of propargyl alcohols **1a**–**i**.

A range of propargyl alcohols **1a**–**i** with varied electronic and steric properties were prepared from electron‐rich and electron‐deficient aldehydes **6a**–**e** and arylalkynes **7a**–**d** (Scheme 2).

LDA deprotonation of arylalkynes **7** and nucleophilic attack of the corresponding lithium acetylide on added aldehyde **6**, gave the desired propargyl alcohol products **1** (41–67 %). The electron‐rich alkyne **1f** was isolated in low yields (18 %), due to challenging purification.

### Au‐Catalyzed Reactions of Propargyl Alcohols with Aryl Nucleophiles

It soon became clear that the reactions of propargyl alcohols **1** with aryl nucleophiles ArH (Scheme 1b,c) were more complex than previously reported. In addition to variable ratios of allene **3** and indene **4** products, also the competing C1 substitution products **2** and **2^solv^** could be formed, depending on the nucleophilic ability of ArH and solvent. Our hypothesis was that the reaction of propargyl alcohols **1** and aryl nucleophiles could be tuned to give either the initially formed allene **3** by C3 substitution or to further proceed to the 1,3‐diaryl‐indene product **4** by intramolecular hydroarylation. Consequently, the scope of the reaction and the allene **3** /indene **4** product selectivity was investigated by varying time, temperature, gold catalyst, solvent (Table 1), propargyl alcohol (Table 2) and aryl nucleophile (Table 3a). Also, the formation of tetraaryl‐allyl products **5** was studied by intermolecular hydroarylation of allene **3** with a second nucleophilic aryl compound (Table 4). ^1^H NMR product quantification of reaction mixtures readily gave essential information of the outcome of the reactions. The relative abundance of formed compounds was determined by integration of selected characteristic ^1^H NMR signals (Table 1) in the spectrum of crude product mixtures. New products **1**–**5** were also synthesized, isolated (% yields, Tables 2, 3) and characterized (^1^H and ^13^C NMR, HRMS). Full ^1^H, and ^13^C NMR data assignments of products **2**–**4** (based on 2D NMR studies; COSY, HSQC, HMBC, NOESY), are available in the Supporting Information.

**Table 1:** Effects of gold catalyst, reaction time, temperature and solvent on reaction outcome.^[a,b]^

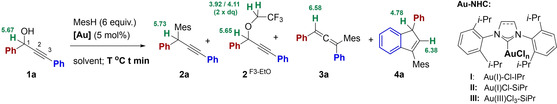										
	**[Au]** (equiv. AgSbF_6_)	Solvent	Product ratio [%]^[b]^ r.t., 15 min				Product ratio [%]^[b]^ 80 °C, 90 min			
Entry			**1a**	**2a** (**2^solv^**)	**3a**	**4a**	**1a**	**2a** (**2^solv^**)	**3a**	**4a**
1	JohnPhosAu^I^(ACN)SbF_6_	F_3_‐EtOH	74	3 (4)	19	0	0	8 (2)	0	90
2	Me_2_SAu^I^Cl	F_3_‐EtOH	74	3 (4)	19	0	0	5	0	95
										
3	AuCl_3_	F_3_‐EtOH	0	10	86	4	0	5	0	90
4	AuBr_3_	F_3_‐EtOH	0	10	85	5	0	7	0	93
5	KAuCl_4_	F_3_‐EtOH	0	9	91	0	0	8	2	90
										
6	AuBr_3_	EtOH	–	–	–	–	0	(**2** ^OEt^ 76)	0	0
7	AuBr_3_	ACN	–	–	–	–	0	≈75	0	≈25
8	AuBr_3_	MeNO_2_	–	–	–	–	0	10	5	85
9	AuBr_3_	DCM^c^	–	–	–	–	35	3	57	5
10	AuBr_3_ (2)	DCM^c^	0	6	90	4	0	6	0	94 (5 h)
										
11	**I** Au^I^Cl‐IPr (1)	F_3_‐EtOH	0	10 (15)	55	20	0	0	0	93 (24 h)
12	**II** Au^I^Cl‐SIPr (1)	F_3_‐EtOH	0	16 (8)	56	20	–	–	–	–
13	**III** Au^III^Cl_3_‐SIPr (1)	DCM	34	5	61	0	–	–	–	–
14	**III** Au^III^Cl_3_‐SIPr (2)	DCM	0	8	92	0	0	0	2	98 (24 h)^[c]^

[a] Standard procedure: A mixture of Au catalyst (5 mol %), propargyl alcohol **1a** (1 equiv. and MesH (6 equiv.) in solvent (1 mL) was stirred at given temperature and time before addition of water, a few drops of NEt_3_ and extraction into diethyl ether, followed by removal of solvent in vacuo. [b] Compound ratios (**1a**, **2a**, **2a^s^** ^olv^, **3a**, **4a**) are based on ^1^H NMR integration of the resulting reaction mixtures. ^1^H NMR shift values of characteristic signals, used for compound identification and quantification in product mixtures, are shown in green numbers above and are given in ppm. [c] DCM reflux at approx. 40 °C.

### Effect of Time, Temperature, Solvent and Gold Catalyst

Initial studies verified that proper choice of reaction time and temperature could favor formation of either allene **3** or indene **4** target products. Hence, the reactions of substrate **1a** with mesitylene nucleophile (MesH, 6 equiv.) in trifluoroethanol (F_3_‐EtOH) were tuned both for selective formation of allene **3a** (r.t., 15 min) and the diaryl‐indene **4a**, (80 °C, 1.5 h). A selection of commercially available Au^I^ and Au^III^ catalysts (5 mol %) were tested (Table 1).

It appeared that the choice of gold salts strongly affected the outcome of the reactions. The two tested Au^I^ salts JohnPhosAu(ACN)SbF_6_ and Me_2_SAuCl (entries 1,2), were weak catalysts for the initial allene **3a** formation (19 % at r.t., 15 min), and large amount of substrate **1a** (74 %) remained unreacted. In contrast, Au^III^ salts (AuCl_3_, AuBr_3_, KAuCl_4_) generated the initial allene **3a** product in high yields (85–91 %; entries 3–5) by similar mild conditions. However, both Au^I^ and Au^III^ salts afforded high conversion into indene product **4a** by heating (90–95 %; 80 °C, 1.5 h; entries 1–5). KAuCl_4_ generated the initial allene **3a** most selectively (91 %); while Me_2_SAuCl afforded the final indene product **4a** most efficiently (95 %) by heating. But only gold(III) salts were unique to allow appropriate temperature tuning of the reactivity to give high yields of both allene **3a** or indene **4a** target products in F_3_‐EtOH.

Previously reported studies concluded that the AuBr_3_‐catalyzed formation of indene **4** was strongly dependent on the solvent, as the reaction was unsuccessful in refluxing toluene and THF, while moderate to high yields were obtained in DCE and CF_3_‐EtOH at reflux.^[30]^ To avoid fluorinated solvents, other more conventional solvents for gold catalysis were attempted for indene formation (Table 1, entries 6–10). Competing nucleophilic C1 substitution by the aryl nucleophile MesH or the solvent (F_3_‐EtOH) was expected to take place to give varying amounts of unwanted by‐products **2a** and **2a^solvent^**. In ethanol, the propargyl ether **2a^OEt^** was mainly formed (76 %, entry 6), while in acetonitrile (entry 7), the C1 aryl substitution product **2a** dominated (**2a**: **4a**; 3 : 1 ratio). As not all gold complexes may be compatible with F_3_‐EtOH, nitromethane could serve as an alternative non‐fluorinated solvent for the reaction. High reactivity and selectivity were obtained in MeNO_2_ (85 % indene **4**, entry 8), but somewhat lower than in F_3_‐EtOH (93 %, entry 4). Reduced reactivity would be expected in DCM, a common solvent for Au catalysis, due to lower reflux temperature. In fact, only modest conversion of substrate **1a** into allene **3a** (57 %) and indene **4a** (5 %) took place in DCM (entry 9).

Standard activation of Au^I^‐Cl pre‐catalysts by anion exchange with an appropriate weakly coordinating anion is performed by addition of 1 equiv. of the respective silver salt. However, increased yields in gold(III)‐catalyzed reactions, including AuCl_3_ and Au^III^‐NHC, have been obtained by increasing the number of equivalents of AgSbF_6_ (from 1 to 2), presumably due to generation of a more electrophilic gold(III) species.[^57^, ^58^, ^59^] In fact, a dramatic effect of the AuBr_3_‐catalyzed reaction in DCM was observed by addition of AgSbF_6_ (2 equiv.), and full conversion of substrate **1a** gave highly selective formation of allene **3a** (90 %, r.t., 15 min) and indene **4a** (94 %, reflux (40 °C), 5 h), respectively (entry 10).

Studies on the catalytic potential of NHC‐Au^I^ and Au^III^ salts (**I**–**III)** (entries 11–14) showed modest catalytic ability and moderate yields of allene **3a** (55–61 %, r.t. 15 min, entries 11–13) in DCM by standard procedure (1 equiv. AgSbF_6_). However, addition of 2 equiv. of silver salt, increased the NHC‐Au^III^ (**III**) catalytic activity to give full conversion and superior amounts of allene **3a** (92 %, DCM, r.t. 15 min, entry 14). Excellent yields of indene **4a** were formed by heating with both Au^I^‐NHC (**I**) in F_3_‐EtOH and NHC‐Au^III^Cl_3_ (**III**) in DCM (93–98 %, 40–80 °C, 24 h, entries 11 and 14).

Thus, highly selective formation of either allene **3a** or indene **4a** from propargyl alcohol **1a** may take place at low and high temperature, respectively. By varying reaction time and temperature, as well as gold catalysts and solvents, the most promising tunable catalytic conditions were obtained by the Au^III^ halide salts (AuCl_3,_ AuBr_3,_ KAuCl_4_) in F_3_‐EtOH, or with gold(III) catalysts AuBr_3_‐(AgSbF_6_ )_2_ and Au^III^Cl_3_‐SIPr‐(AgSbF_6_)_2_ in DCM. The scope of the reaction was therefore further studied.

### Effect of Diarylpropargyl Alcohol Properties

In order to study how the electronic and steric characteristics of propargyl substrate **1** may impact the Au‐catalyzed reaction, a series of modified propargyl alcohols **1‐Ar^1^**‐**Ar^2^** were reacted with mesitylene in the presence of AuBr_3_ at mild and more harsh reaction conditions (20‐80 °C in F_3_‐EtOH; Table 2). Due to steric effects by aryl‐incorporation for formation of indene products **4** (Scheme 1c) the cyclization reaction only took place with non‐substituted phenyl Ar^2^ group (Ar^2^=Ph). Thus, a prerequisite for successful, tunable, and selective allene and indene synthesis was the use of 3‐phenyl‐propargyl alcohol substrates (**1‐Ar^1^‐Ph**), such as **1‐Ph‐Ph**, **1‐(2,6‐diMe)Ph‐Ph** and **1‐Mes‐Ph** (**1a**,**1b**,**1c**) with electron‐rich and/or bulky Ar^1^ groups. These substrates had great ability to allow temperature fine‐tuning to afford selective and nearly quantitative formation of either allenes (**3a**–**c**, 85–100 %, r.t.) or subsequent intramolecular hydroarylation indene products (**4a**–**c**, 93–100 %) at low or high temperature, respectively (entries 1, 2, 3)

**Table 2:** Studies on diarylpropargyl alcohol **1** properties.^[a]^

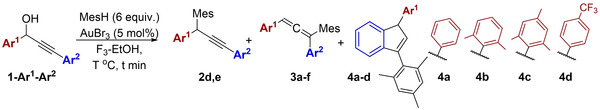										
		Product ratio [%]^[b]^ (% isol. yield) r.t. 15 min					Product ratio [%]^[b]^ (% isol. yield) 80 °C, 90 min			
Entry	**1‐Ar^1^‐Ar^2^**	**1**		**2**	**3‐Ar^1^‐Ar^2^**	**4**	**1**	**2**	**3**	**4‐Ar^1^‐Ar^2^**
	**Ar^2^=Ph**:									
1	**Ar^1^=Ph**	**1a**	0	10	**3a**: 85 (40)	5	0	7	0	**4a**: 93 (92)
2	**Ar^1^=1‐(2,6‐diMe)Ph**	**1b**	0	0	**3b**: 98 (81)	2	0	0	0	**4b**: >99 (78)
3	**Ar^1^=1‐Mes**	**1c**	0	0	**3c**: >99 (37)	0	0	0	0	**4c**: >99 (50)
4	**Ar^1^=1‐(4‐CF_3_)Ph**	**1d**	4	**2d**: 53	**3d**: 43 (19)	0	0	**2d**: 5	0	**4d**: 69 (29) **4d‐Br** 31^d^
5	**Ar^1^=1‐(4‐OMe)Ph**	**1e**	–^c^	–^c^	–^c^	–^c^	–	–	–	–
										
	**Ar^1^=Ph**:									
6	**Ar^2^=1‐(4‐OMe)Ph**	**1f**	<10	0	0	0	–^e^	–	–	–
7	**Ar^2^=1‐(4‐CF_3_)Ph**	**1g**	0^e^	**2g**: 65	**3g**: 26	0		–	–	–
8	**Ar^2^=1‐Mes**	**1h**	0^e^	0	0	0	–^e^	–	–	–
										
9	**Ar^1^=1‐Mes**, **Ar^2^=(4‐CF_3_)Ph**	**1i**	5^e^	0	**3i** 95 (40)	0	–	–	–^e^	–

[a] Standard procedure: AuBr_3_ (5 mol %) with propargyl alcohol **1** (1 equiv.) and MesH (6 equiv.) in F_3_‐EtOH (1 mL). The mixture was stirred at T °C for t min before addition of water, a few drops of NEt_3_ and extraction into diethyl ether followed by removal of solvent in vacuo. [b] Compound ratios (**1**, **2**, **3**, **4**) are based on ^1^H NMR integration of the resulting reaction mixtures. [c] Complex product mixture; including the **2e^F3−EtO^** product; which was prepared in 28 % yield for identification. [d] Additional 5 mol % AuBr_3_ added to reaction mixture; 80 °C for 5 h. Products **4d** and **4d‐Br** could not be separated. [e] Unidentified product mixture.

The electron‐rich propargyl alcohols **1‐(4‐OMe)Ph‐Ph**, **1‐Ph‐(4‐OMe)Ph** and **1‐Ph‐Mes** (**1e**, **1f**, **1h**; entries 5, 6, 8) were not appropriate substrates for aryl nucleophilic C3 substitution, and the anisole and mesitylene substrates gave no conversion or complex mixture of products at r.t. or by heating. On the other hand, the electron deficient substrates **1‐(4‐CF_3_)Ph‐Ph** and **1‐Ph‐(4‐CF_3_)Ph** (**1d**, **1g**; entries 4, 7) mainly activated for unwanted C1 substitution byproducts **2d** and **2g** (53/65 %), along with allenes **3d** and **3g** (43/26 %). Allene **3d** did undergo cyclization into indene **4d** by heating. Unexpectedly, C1‐substitution product **2d** also gave indene **4d** by slow rearrangement. Hence, by 5 h reaction time and 10 mol % AuBr_3_, indene **4d** (64 %) and an additional inseparable indene product (**X**, 31 %) was formed (entry 5). Treatment of the (**4d**+**X**) mixture with *meta*‐chloroperoxybenzoic acid (MCPBA) enabled separation of unreacted unknown indene **X** from a mixture of unidentified products. The unknown **X** was shown (NMR, HRMS) to be the 2‐Br‐indene derivative **4d‐Br**, which probably is formed by electrophilic bromination, due to AuBr_3_ decomposition into nanogold/Au(0). A pure sample of 2‐Br‐indene **4d‐Br** (entry 5) was prepared for characterization, by NBS in situ treatment of allene **3d**, and the 2‐bromo‐structure was confirmed.

Attempts to slow down the C1‐aryl substitution at 0 ${}^{\circ }$ C resulted in no substrate conversion at all. However, by modifying substrate **1‐Ph‐(4‐CF_3_)Ph** (**1g**) by blocking unwanted C1 substitution with introduction of *ortho*/*para* methyl groups (Ar^1^=mesitylene), full conversion of the electron deficient substrate **1‐Mes‐(4‐CF_3_)Ph** (**1i**) took place into the corresponding allene **3i** (95 %, entry 9). The electron deficient allene **3i** was, however, unstable and did not undergo further indene cyclization, in contrast to the successful cyclization of allene **3b**, formed from the other *ortho*‐2,6‐diMe‐blocked electron‐rich substrates **1b**, **1c** (entries 2, 3).

The results showed that gold(III)‐catalyzed reactions of modified diaryl‐propargyl alcohols **1‐Ar^1^‐Ar^2^** with mesitylene to give selective allene **3** or indene **4** products are highly sensitive to electronic and steric factors in both aryl groups. However, 1‐aryl‐3‐phenyl‐propargyl alcohols (**1‐Ar^1^‐Ph**), with electron‐rich and/or bulky Ar^1^ groups were excellent substrates for selective allene **3** and indene **4** synthesis (up to 99 %). The pure allenes **3a**–**d**,**g**,**i** and indene **4a**–**d** products were prepared for characterization (NMR, HRMS).

### Effect of Aromatic Nucleophiles, Ar^3^H and Ar^4^H

**Scheme 3:**
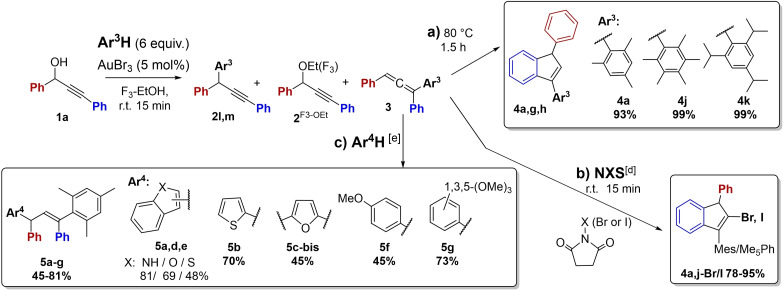
Studies of aromatic nucleophiles Ar^3^H and Ar^4^H in on‐pot tandem formation of a) indenes **4** by intramolecular hydroarylation; b) halo‐indenes **4‐X** by intermolecular haloarylation and c) tetraaryl‐allyl products **5** by intermolecular hydroarylation.

**Allene 3 and Indene 4 Target Products**: A study of allene/indene formation was performed with substrate **1a** and different aromatic nucleophiles Ar^3^H with varied electron density and steric properties (Table 3a, Scheme 3a). Only the reaction of pentamethylbenzene (entry 2) followed the original selective AuBr_3_‐catalyzed pathways (C3 substitution/intramolecular hydroarylation), but even higher yields were obtained than with mesitylene (entry 1; from Table 2), as the allene **3j** and indene **4j** products were quantitatively formed at room temperature and by reflux conditions, respectively. The reaction with the 1,3,5‐triisopropylbenzene nucleophile gave an unexpected outcome (entry 3), as no allene intermediate was seen, while the C1 solvent substitution product **2a^F3‐OEt^** (>80 % at r.t.) was readily formed and directly generated the final indene product **4k** (<99 %) under reflux conditions. A separate control experiment with a purified sample of the propargyl ether product **2a^F3‐EtO^** and mesitylene confirmed that quantitative formation of indene **4a** directly took place without any observation of the expected allene intermediate **3a**. The electron‐rich and strong Ar^3^H aromatic nucleophiles anisole and the bulky 1,3,5‐trimethoxybenzene favored unwanted C1 substitution products **2l** and **2m** (entries 4, 5, 80–99 %). A series of other aryl compounds (ethylbenzene, *p*‐xylene, toluene, styrene, *tert‐*butylbenzene, phenyl acetate, acetanilide, *N‐*Ts‐anilide, nitrobenzene, 1,3‐di‐CF_3_‐benzene, *N*‐methylpyrrole, thianaphthene) failed to give allene **3** or indene **4** products.

**Table 3:** Properties of aromatic nucleophiles Ar^3^H in formation of indene products **4**.^[a,b]^

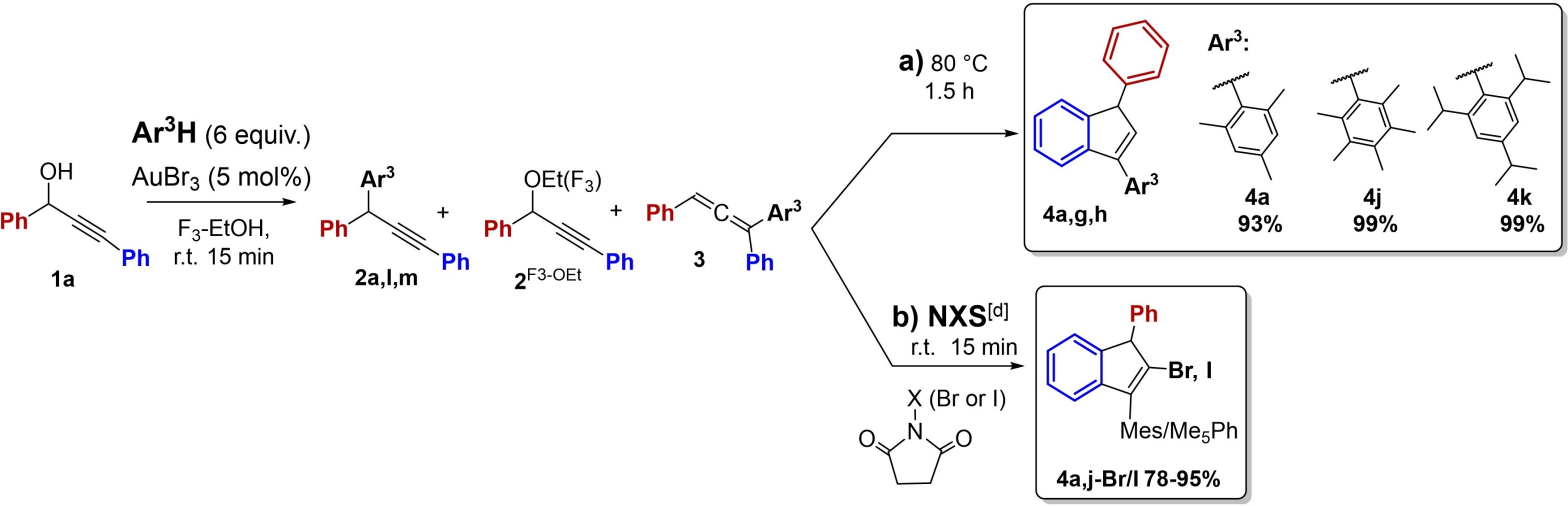						
Entry	***Reactants:*** a) **1a**+**Ar^3^H**	Product ratio [%] (% isol. yield) r.t., 15 min			Product ratio [%] (% isol. yield) 80 °C, 90 min	
		**2**	**3**	**4**	**2**	**4**
1	Mesitylene (Table 2)	**2a** 10	**3a** 85 (40)	5	**2a** 7	**4a** 93 (92)
2	PentaMe‐Ph	–	**3j**: >99 (99)	0	–	**4j**: >99 (56) ^[c]^
3	1,3,5‐tri‐iPr‐Ph	(**2a** ^F3‐OEt^>80 (23)) ^[c]^	**0**	0	0	**4k**: >99 (15) ^[c]^
4	Anisole	**2l**: >99 (77)	–	–	**2l**: >99 (77)	–
5	1,3,5‐triOMe‐Ph	**2m**: >80^[c]^ (52)	–	–	**2m**: >80^[c]^ (52)	–

	b) **1a**+**Ar^3^H**+**NXS** ^[d]^				Product ratio [%] (% isol. yield) r.t., 15 min	
6	Mesitylene+NIS				**2a** 10	**4a**–**I** 90 (68)
	PentaMe‐Ph+NIS				**2** ^F3−OEt^ 5	**4j**–**I** 95 (82)
7	Mesitylene+NBS				**2a** 22	**4a**–**Br** 78 (56) ^[c]^

[a] Standard procedure: AuBr_3_ (5 mol %) with propargyl alcohol **1** (1 equiv.) and aryl nucleophile Ar^3^H (6 equiv.) in F_3_‐EtOH (1 mL). The mixture was stirred at T °C for t min before addition of water, a few drops of NEt_3_ and extraction into diethyl ether followed by removal of solvent in vacuo. [b] Compound ratios are based on ^1^H NMR integration of the resulting reaction mixtures. [c] Additional unidentified products. [d] One‐pot reaction; after full conversion into allene **3a** (r.t, 15 min), NXS (1.1 equiv.) was added to the reaction mixture which was subsequently stirred at r.t. for 15 min.

Thus, successful tunable formation of target allene **3** or indene **4** products seems to require moderately activated Ar^3^H alkylbenzene nucleophiles, while strong alkoxyaryl nucleophiles react by undesired C1 substitution. The pure allene **3j** (99 %) and indenes **4j** and **4k** (unstable; 56 % and 15 %) were prepared for characterization (NMR, HRMS).

The developed haloarylation strategy to synthesize functionalized 2‐haloindenyl from propargylic alcohol substrates represents a gold‐catalyzed tandem reaction with aryl carbon nucleophiles, in contrast to other similar iodination reactions, which include heteroatom nucleophiles.[^61^, ^62^, ^63^, ^64^] The 2‐halo‐sp^2^‐carbon moiety represents a versatile reactive position for subsequent transformations, such as a series of efficient Pd‐catalyzed C−C coupling reactions, which may give rise to a great variety of indene based target products.

    

**2‐Haloindenes**: As arylallenes are known to provide 2‐iodoindenes by iodocarbocyclization in the presence of NIS,^[60]^ a one‐pot iodoarylation strategy from propargylic alcohol **1a** was tested for iodo‐incorporation in products **4** (Table 3b, Scheme 3b). Actually, iodo‐modified hydroarylation reaction conditions successfully allowed for electrophilic iodination through intramolecular iodoarylation of triarylallene **3a** and **3j** intermediates by in situ addition of NIS (*N*‐iodo‐succinimide) to the respective allene (**3a**,**j**) reaction mixtures (r.t., 15 min.). Thus, the corresponding 2‐iodo‐diaryl‐indene products **4a**–**I** and **4j**–**I** (90–95 %, entry 6) were formed in one‐pot gold(III)‐catalyzed transformations of propargylic alcohol **1a**. No competing aromatic halogenation took place at the phenyl groups by this chemoselective reaction. As expected, the corresponding NBS reactions were somewhat less efficient than the NIS haloarylations, and 78 % of the corresponding bromo‐arylation product **4a‐Br** was formed (entry 7) from substrate **1a**. As discussed above (Table 2, entry 5), lower amounts of the 2‐Br‐derivative **4d‐Br** were generated from the electron‐deficient substrate **1d** and NBS. The pure 2‐haloindene products **4a**–**I** (68 %), **4j**–**I** (82 %) and **4a‐Br** (56 %) were prepared for characterization (NMR, HRMS).

    

**Tetraaryl‐allyl Products 5**: Attempts with allene **3a** to follow an alternative competing gold(III)‐catalyzed intermolecular hydroarylation pathway with a second external aryl nucleophile (Ar^4^H) to afford 1,1,3,3‐tetraaryl‐allyl products **5** were promising (Table 4, Scheme 3c). By a one‐pot procedure, addition of the heterocyclic indole to the initially formed allene **3a** reaction mixture, efficient incorporation of 3‐indole took place by intermolecular hydroarylation. The amount of target tetraaryl‐allyl product **5a** increased by heating from 1.5 h to 6 h (27–81 %; 80 °C, entries 1,2). The preferred site for electrophilic substitution on indole is C3 rather than C2, as expected from the greater electron density at C3 of the enamine structure moiety and higher stabilization of the iminium cation formed by C3 attack. In contrast to most aryl nucleophiles Ar^4^H below (entries 6–10), the indole reactions afforded target 3‐indol‐substituted product **5a** as *E*/*Z* double‐bond stereoisomers (6 : 1). The facts that no conversion took place at r.t., and that the competing indene by‐product **4a** was only formed in minor amounts (5 %), may indicate that the gold(III)‐catalyzed indole hydroarylation follows a different reaction mechanism than the other aryl nucleophiles.

**Table 4:** Properties of aromatic nucleophiles Ar^4^H in formation of tetraaryl‐allyl products **5**.^[a,b]^

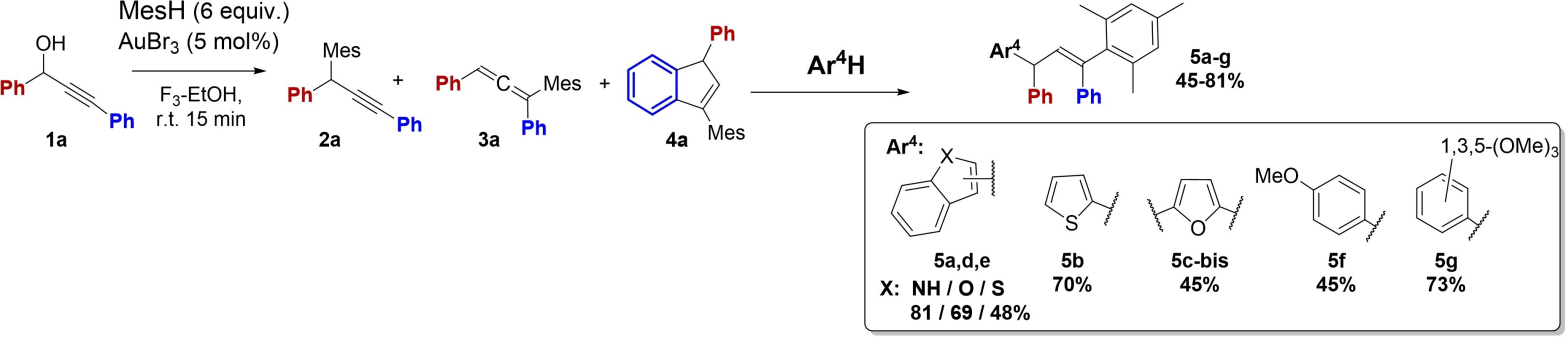							
Entry	***Reactants:*** **3a+Ar^4^H** (equiv.) ^[c]^	Temperature, Time	Product ratio [%] (% isol. yield)				
			**2a**	**3a**	**4a**	**5**	regio‐isomer (E,Z ratio)
1	Indole (1)	80 °C, 1.5 h	13	55	5	**5a** 27	3‐pos. (6 : 1^[d]^)
2	Indole (1)	80 °C, 6 h	14	0	5	**5a** 81 (37)	3‐pos. (6 : 1^[d]^)
3	Thiophene (1)	80 °C, 1.5 h	4	0	51	**5b** 20 (16) **5b‐bis** 25 (16)	2‐pos. 2,5‐pos
4	Thiophene (5)	80 °C, 1.5 h or r.t., 24 h	10	0	30	**5b** 32 (27) **5b‐bis** 28 (24)	2‐pos. (5 : 1^[d]^) 2,5‐pos
5	Thiophene (0.5)	r.t., 24 h	15	0	38	**5b** 4 **5b‐bis** 43 (37)	2‐pos. 2,5‐pos
6	Furan (1)	80 °C, 1.5 h	17	0	38	**5c‐bis** 45 (20)	2,5‐pos.
7	Benzofuran (1)	r.t., 24 h	10	0	21	**5d** 69 (53)	2‐pos
8	Benzothiophene (1)	r.t., 24 h	8	0	44	**5e^2,3^** 48 (32)	2‐,3‐pos.(1 : 1^[e]^)
9	Anisole (1)	r.t., 24 h	10		45	**5f** 45 (42)	4‐pos.
10	1,3,5‐(OMe)_3_‐Ph (1)	r.t., 24 h	9	0	18	**5g** 73 (58)	
11	Benzofuran, benzothiophene, anisole, 1,3,5‐(OMe)_3_‐Ph	80 °C, 1.5 h	5–10	0	90–95	0	
12	(*N*‐Me‐)pyrrole (1)	r.t., 48 h	10	85	5	0	
13	Furan (5 equiv.), (Me‐)pyrrole (1)	80 °C, 1.5 h	Complex product mixtures				

[a] Standard procedure: AuBr_3_ (5 mol %) with propargyl alcohol **1** (1 equiv.) and aryl nucleophile Ar^3^H (6 equiv.) in F_3_‐EtOH (1 mL). The mixture was stirred at T °C for t min before addition of water, a few drops of NEt_3_ and extraction into diethyl ether followed by removal of solvent *in vacuo*. [b] Compound ratios are based on ^1^H NMR integration of the resulting reaction mixtures. [c] One‐pot reaction; after full conversion into allene **3a** (r.t, 15 min), the second nucleophile, Ar^4^H (1–5 equiv.) was added to the reaction mixture and stirred at T °C for t hours. [d] ratio double‐bond stereoisomers (*E*/*Z*). [e] Ratio of regio‐isomers.

Our results for intermolecular hydroarylation with five‐membered heterocycles were in accordance with expected reactivity and positional selectivity. The order of reactivity in electrophilic substitution of these heterocycles has been shown to be thiophene<furan≪pyrrole.^[65]^ In contrast, it is known that the C3‐/C2‐positional selectivity (β : α ratio) of five‐membered heterocycles increases with increasing ability of the heteroatoms to stabilize the corresponding onium states of the elements (O+<S+<N+).^[65]^ The less reactive thiophene did undergo a more efficient hydroarylation with a large excess of thiophene (5 equiv., entries 3–4), also without heating. The thiophene nucleophile gave regioselective 2(α)‐substitution products **5b** (obtained as a 5 : 1 mixture of *E/Z* isomers) as sulfur provides insufficient stabilization relative to the indole cation giving 3‐substitution. Thiophene did additionally undergo double hydroarylation to give mixtures of 2‐mono‐ and 2,5‐bis‐products **5b** and **5b‐bis** (32 %; 28 %; entry 4). However, almost selective formation of the bis‐product **5b‐bis** (43 %) was observed with a limited amount of thiophene (0.5 equiv., entry 5), demonstrating the higher nucleophilic ability of the mono‐product **5b** to undergo a second hydroarylation. We attempted to identify the double‐bond stereoselectivity of the indole and thiophene product **5a** and **5b** product mixtures by NOESY NMR but no conclusive data were obtained. However, based on steric factors of the bulky tetraaryl‐allyl structure, the *E*‐double bond would be expected in all products **5**, as shown in Scheme 3.

The furan nucleophile also demonstrated the expected preference for α‐substitution. Being more electron‐rich and reactive, furan favored complete bis‐hydroarylation and gave the 2,5‐bisfuran product **5c‐bis** (45 %, 80 °C, 1.5 h, entry 6) from equimolar reactions, while benzofuran afforded mono‐hydroarylation product **5d** by C2 attack (69 %, r.t., 24 h, entry 7). Electrophilic substitution of benzothiophene usually give both α and β isomers. Thus, the benzothiophene reactions gave 1 : 1 mixtures of C2‐/C3‐regioisomers **5e^2^** and **5e^3^** (48 % in total, entry 8). Benzothiophene was less reactive towards the electrophile than thiophene (60 %, entry 4). Both the slightly and the highly activated phenyl derivatives, anisole and 1,3,5‐triOMe‐benzene, successfully afforded the respective tetraaryl‐allyl products **5f**,**g** in variable degrees by similar conditions (45–73 %, r.t., 24 h, entries 9, 10). Selective *para* attack afforded anisole‐product **5f**.

At higher temperature, benzofuran, benzothiophene, anisole and 1,3,5‐tri‐OMe‐benzene were not incorporated by intermolecular hydroarylation, as the competing indene **4a** (90–95 %) formation took place by selective intramolecular hydroarylation (entry 11). By similar harsh conditions, the reactive furan, pyrrole and *N*‐Me‐pyrrole Ar^4^H nucleophiles only gave undefined product mixtures via allene **3a** (entries 12, 13). By addition of aniline or hydrazine‐Boc nucleophiles to the allene **3a** reaction mixture at r.t., no further reaction took place and allene **3a** was recovered, probably due to amine *N*‐coordination and deactivation of the gold catalyst, demonstrating that gold catalysis is required for subsequent indene **4a** formation.

Thus, the one‐pot intermolecular hydroarylation tandem process was successful with several aryl nucleophiles Ar^4^H, which provided tetraaryl‐allyl products **5** as mixtures of stereo‐, regio‐isomers or mono/bis‐adducts, depending on Ar^4^H nucleophile properties. The indole, benzofuran, thiophene and 1,3,5‐triOMe‐benzene nucleophiles afforded highest yields of target products **5a**–**g** (60‐81 % yield). The pure tetraaryl‐allylic **5a**–**g** as well as the **5b‐bis** and **5c‐bis** products were prepared for characterization (NMR, HRMS).

## Conclusion

Gold‐catalyzed transformations of diarylpropargyl alcohols **1** with aryl nucleophiles were studied in order to identify the most promising *i) catalytic conditions*, ii) *propargyl alcohol substrates **1*** and *iii) aryl nucleophiles* for tunable and selective preparation of 1,1,3‐triallylallenes **3**, diaryl‐indenes **4** or 1,1,3,3‐tetraaryl‐allyl **5** products.

- Optimized conditions for highly selective formation of allene **3a** or indene **4a** from diphenylpropargyl alcohol **1a** and mesitylene nucleophile were developed by varying reaction time and temperature, as well as gold catalysts and solvents. The most promising and tunable catalytic conditions (up to 92 % **3a**/98 % **4a**) were obtained with AuX_3_ halide salts in F_3_‐EtOH, or with gold(III) catalysts Au^III^Br_3_‐(AgSbF_6_)_2_ and Au^III^Cl_3_‐SIPr‐(AgSbF_6_)_2_ in DCM.
- Further studies showed that selective gold(III)‐catalyzed reactions of modified diarylpropargyl alcohols **1‐Ar^1^‐Ar^2^** with mesitylene are sensitive to electronic and steric factors in both aryl groups. The 3‐phenyl‐propargyl alcohols **1‐Ar^1^‐Ph** with electron‐rich and/or bulky Ar^1^ groups (**1‐Ph‐Ph**, **1‐Mes‐Ph**, **1‐(2,6‐diMe)Ph‐Ph**) allowed successful temperature fine‐tuning of reactivity to afford selective and quantitative formation of allene **3a**–**d** or indenes **4a**–**d**.
- Generally, successful tunable and selective formation of initial allenes **3** (by C3 substitution at r.t.) or subsequent indene **4** products (by intramolecular hydroarylation by heating) required moderately activated Ar^3^H alkylbenzene nucleophiles. Stronger alkoxyaryl nucleophiles reacted by undesired C1 substitution and failed to react by the tunable dual pathways. The present allene‐indene synthetic strategy is useful for further indene functionalization, such as halogenation, as shown by the chemoselective formation of corresponding 2‐iodo and 2‐bromo indenes (**4‐I**, **4‐Br**; 78–95 %) by one‐pot intramolecular allene **3** haloarylation in the presence of NXS. The 2‐haloindenes are appropriate substrates for further Pd‐catalyzed reactions.

An alternative intermolecular hydroarylation of allene **3a** with a second external aryl nucleophile Ar^4^H provided tetraaryl‐allyl products **5** (up to 81 % yield). Several strong nucleophiles (indole, thiophene, furan, benzofuran, benzothiophene, anisole and 1,3,5‐triOMe‐Ph) successfully followed this reaction pathway by a sequential one‐pot tandem process. Specific stereo‐, regio‐isomers or mono/bis versions of tetraaryl‐allyl products **5** were identified for each group of heteroaromatic nucleophiles.

## Experimental Section

### General

All reactions, except the synthesis of gold complexes, were performed under inert N_2_‐atmosphere. Commercial grade reagents were used without any additional purification. Dry solvents were collected from a MB SPS‐800 solvent purification system. All reactions were monitored by NMR and/or thin‐layer chromatography (TLC) using silica gel 60 F254 (0.25 mm thickness). TLC plates were developed using UV‐light, *p*‐anisaldehyde stain, or I_2_ stain. Flash chromatography was performed with Merck silica gel 60 (0.040–0.063 mm). ^1^H and ^13^C NMR spectra were recorded by a Bruker Avance DPX 400 MHz or a Bruker Avance III 600 MHz spectrometer. Chemical shifts are reported in ppm (δ) downfield from tetramethylsilane (TMS) as an internal standard. Coupling constants (*J*) are given in Hz. Specific NMR assignments (^1^H, ^13^C) of synthesized and purified products **2**–**4** below, based on 2D NMR studies (COSY, HSQC, HMBC, NOESY), are available in Supporting Information. Accurate mass determination (HRMS) was performed on a “Synapt G2‐S” Q‐TOF instrument from Water TM. Samples were ionized with an ASAP probe (APCI) or ESI probe with no chromatographic separation performed prior to mass analysis. Calculated exact mass and spectra processing was done by Waters TM Software Masslynx V4.1 SCN871.

### Preparation of Diarylpropargyl Alcohols (1)

***General Procedure A***: A solution of arylacetylene **7a**–**d** (1–1.1 equiv.) in dry THF was cooled to 0 °C and LDA (1.5 equiv., 2 m in THF) was added slowly under a N_2_‐atmosphere. The solution was stirred for 30 min before addition of aldehyde **6a**–**e** (1 equiv.). The solution was stirred for 2 h and was allowed to warm to r.t. before being quenched with aqueous NH_4_Cl (sat., 10 mL). The layers were separated, and the aqueous phase was extracted with EtOAc (3×15 mL). The combined organic layers were washed with brine, dried over Na_2_SO_4_, and the solvent was removed in vacuo. Purification by flash column chromatography (EtOAc:pentane) yielded pure propargyl alcohols **1a**–**i**.

### Preparation of C1 Substitution Products (2)

See Supporting Information for preparation of products **2a**,**d**,**g**,**I**,**m**; **2a^OEt^**; **2h^F3−OEt^**.

### Preparation of 1,1,3‐Triarylallenes (3)

***General procedure B***: Propargyl alcohol **1** (1 equiv.) and an aromatic nucleophile (1–6 equiv.) were dissolved in either F_3_‐EtOH or MeNO_2_ (1 mL). A solution of AuBr_3_ (0.05 equiv.) in the same solvent (1 mL) was added, and the solution was stirred at r.t. for 15 min. H_2_O (5 mL), a few drops of NEt_3_ and diethyl ether (5 mL) were added and the layers were separated. The aqueous layer was extracted with diethyl ether (3×10 mL), and the combined organic layers were dried over Na_2_SO_4_, followed by removal of the solvent in vacuo. Purification by flash column chromatography (1 : 200 EtOAc:pentane) yielded allenes **3**.

### Preparation of Diarylindenes (4)

***General Procedure C***: Propargyl alcohol **1** (1 equiv.) and an aromatic nucleophile (1–6 equiv.) were dissolved in either F_3_‐EtOH or MeNO_2_ (1 mL). A solution of AuBr_3_ (0.05 equiv.) in the same solvent (1 mL) was added, and the solution stirred at 80 °C for 1.5 h. H_2_O (5 mL), a few drops of NEt_3_ and diethyl ether (5 mL) were added, and the layers separated. The aqueous layer was extracted with diethyl ether (3×5 mL), and the combined organic layers were dried over Na_2_SO_4_, followed by removal of solvent in vacuo. Purification by flash column chromatography (1 : 200 EtOAc:pentane) yielded indenes **4**.

### Preparation of 2‐Halo‐indenes (4‐X)

***General Procedure D***: Propargyl alcohol **1a** (1 equiv.) and aromatic nucleophile (1–6 equiv.) were dissolved in 1 mL F_3_‐EtOH and AuBr_3_ (0.05 equiv.) was added. The resulting mixture was stirred for 15 min at room temperature. After conversion to the allene **3a/j** was complete, NXS (1.1 equiv.) was added, and the reaction was stirred for 15–120 min. After completion of the reaction, diethyl ether was added and the organic phase was washed with sat. NaHCO_3_, followed by washing with brine, drying over anhydrous Na_2_SO_4_ and evaporation of the solvent in vacuo. The crude products were then purified by column chromatography.

### Preparation of Tetraaryl‐allyl Compounds (5)

***General Procedure E***: Propargyl alcohol **1a** (1 equiv.) and mesitylene (6 equiv.) were dissolved in 1 mL F_3_‐EtOH and AuBr_3_ (0.05 equiv.) was added. The resulting mixture was stirred for 15 min at room temperature. After conversion to the allene **3a** was complete, the aryl nucleophile (1–10 equiv.) was added, and the reaction mixture was stirred at an appropriate temperature and time. After completion of the reaction, diethyl ether was added and the organic phase washed with water, followed by washing with brine, drying over anhydrous Na_2_SO_4_ and evaporation of the solvent in vacuo. The crude products were then purified by column chromatography.

## Conflict of interest

The authors declare no conflict of interest.

1

## Supporting information

As a service to our authors and readers, this journal provides supporting information supplied by the authors. Such materials are peer reviewed and may be re‐organized for online delivery, but are not copy‐edited or typeset. Technical support issues arising from supporting information (other than missing files) should be addressed to the authors.

Supporting Information

## Data Availability

The data that support the findings of this study are available in the supplementary material of this article.
