# Supplementary material for: Tunable Gold‐catalyzed Reactions of Propargyl Alcohols and Aryl Nucleophiles
Source: ChemistryOpen. 2022 Mar 10;11(5):e202200030. doi: 10.1002/open.202200030 (PMC9059295; doi:10.1002/open.202200030)
Supplement: Supplementary file 1 — Supporting Information [file OPEN-11-e202200030-s001.pdf]

ChemistryOpen

Supporting Information

Tunable Gold-catalyzed Reactions of Propargyl Alcohols and Aryl Nucleophiles

Helgi Freyr Jónsson, Thomas Nordbø Solvi, Sondre Lomeland, Ann Christin Reiersølmoen, and Anne Fiksdahl*

Supporting Information

**Experimental section;** preparation of compounds **1-5**	S2-S9
References	S10
**^{1}H and ^{13}C NMR spectra:**	S11-S46
Propargyl alcohols **1a-i**	S11-17
C1 subst. products **2a,d,g,l,m**	S18-S22
Allenes **3a-g**	S23-S28
Indenes **4a-d,j,k**	S29-S32
Halo-products **4a-Br, 4d-Br, 4a-I, 4j-I**	S33-S36
Tetra-arylallyl products **5a-g**	S37-S46
**NMR assignments** (^{1}H, ^{13}C) of selected products (**2-4**)	S47-S48

Experimental section

General: All reactions, unless stated, were performed under ambient atmosphere. Commercial grade reagents were used without any additional purification. Dry solvents were collected from a MB SPS-800 solvent purification system. All reactions were monitored by NMR and/or thin-layer chromatography (TLC) using silica gel 60 F254 (0.25 mm thickness). TLC plates were developed using UV-light, *p*-anisaldehyde stain, or I_2 stain. Flash chromatography was performed with Merck silica gel 60 (0.040- 0.063 mm). ^{1}H and ^{13}C NMR spectra were recorded by a Bruker Avance DPX 400 MHz or a Bruker Avance III 600 MHz spectrometer. Chemical shifts are reported in ppm (δ) downfield from tetramethylsilane (TMS) as an internal standard. Coupling constants (*J*) are given in Hz. Specific NMR assignments (^{1}H, ^{13}C) of synthesized and purified products **2-4** below, based on 2D NMR studies (COSY, HSQC, HMBC, NOESY), are available below. Accurate mass determination (HRMS) was performed on a "Synapt G2-S" Q-TOF instrument from Water TM. Samples were ionized with an ASAP probe (APCI) or ESI probe with no chromatographic separation performed prior to mass analysis. Calculated exact mass and spectra processing was done by Waters TM Software Masslynx V4.1 SCN871.

***Preparation of diarylpropargyl alcohols (1); General procedure A*:** A solution of arylacetylene **7a-d** (1-1.1 eq.) in dry THF was cooled to 0 °C and LDA (1.5 eq., 2 M in THF) was added slowly under a N_2-atmosphere. The solution was stirred for 30 min before addition of aldehyde **6a-e** (1 eq.). The solution was stirred for 2 h and allowed to warm to r.t. before being quenched with aqueous NH_4Cl (sat., 10 mL). The layers were separated, and the aqueous phase was extracted with EtOAc (3x15 mL). The combined organic layers were washed with brine, dried over Na_2SO_4, and the solvent removed *in vacuo*. Purification by flash column chromatography (EtOAc:pentane) yielded pure propargyl alcohols **1a-i.**

1,3-Diphenylprop-2-yn-1-ol (1a). Following general procedure A, acetylene **7a** (539 mg, 5.28 mmol) in THF (10 mL) was reacted with LDA (3.6 mL, 2 M, 7.20 mmol). Addition of aldehyde **6a** (509 mg, 4.80 mmol), 4.80 mmol) yielded propargyl alcohol **1a** (647 mg, 65 %) as a pale-yellow oil after flash column chromatography (1:10 EtOAc:pentane). ^{1}H NMR (600 MHz, $CDCl_3$) δ (ppm): 7.61 (d, J = 7.1, 2H), 7.46 (m, 2H), 7.39 (t, *J* = 7.1, 2H), 7.36-7.25 (m, 4H), 5.67 (d, *J* = 6.1, 1H), 2.45 (d, 1H, OH). ^{1}H NMR was in accordance with literature.[1]

1-(2,6-Dimethylphenyl)-3-phenylprop-2-yn-1-ol (1b). Following general procedure A, acetylene **7a** (475 mg, 4.65 mmol) in THF (3 mL) was reacted with LDA (3.17 mL, 2 M, 6.34 mmol). Addition of aldehyde **6c** (565 mg, 4.23 mmol) yielded propargyl alcohol **1b** (666 mg, 67 %) as a green solid after flash column chromatography (1:9 EtOAc:pentane). ^{1}H NMR (400 MHz, $CDCl_3$) δ (ppm): 7.38-7.45 (m, 2H), 7.27-7.33 (m, 3H), 7.12 (dd, *J* = 8.4, 6.5, 1H), 7.05 (m, 2H), 6.16 (d, *J* = 3.6, 1H), 2.60 (s, 6 h), 2.10 (d, *J* = 3.9, 1H, OH). ^{1}H NMR was in accordance with literature data.[3]

1-Mesityl-3-phenylprop-2-yn-1-ol (1c). Following general procedure A, acetylene **7a** (612 mg, 5.99 mmol) in THF (3 mL) was reacted with LDA (4.0 mL, 2 M, 8.0 mmol). Addition of aldehyde **6b** (591 mg, 3.99 mmol) yielded propargyl alcohol **1c** (630 mg (2.51 mol 63 %) as a yellow oil after flash column chromatography (1:9 EtOAC:pentane). ^{1}H NMR (600 MHz, $CDCl_3$) δ (ppm): 7.38-7.44 (m, 2H, H9), 7.25-7.32 (m, 3H, H10 and H11), 6.87 (s, 2H, H6), 6.11 (s, 1H, H1), 2.55 (s, 6 h, Me5), 2.27 (s, 3H, Me7), 2.10 (bs, 1H, OH); ^{13}C NMR (150 MHz, $CDCl_3$) δ (ppm): 137.8, 136, 133.6, 131.6, 130.0, 128.3, 128.2, 122.8, 88.8, 85.7, 60.8, 20.9, 20.3. HRMS (ASAP) calcd for $C_{18}H_{17}$ $[M\text{-}OH]^+$ 233.1330, obsd 233.1330.

3-Phenyl-1-(4-(trifluoromethyl)phenyl)prop-2-yn-1-ol (1d). Following general procedure A, acetylene **7a** (406 mg, 3.98 mmol) in THF (5 mL) was reacted with LDA (2.8 mL, 2 M, 5.6 mmol). Addition of aldehyde **6e** (630 mg, 3.62 mmol) yielded propargyl alcohol **1d** (652 mg, 65 %) as an orange oil after flash chromatography (1:9 EtOAc:pentane). ^{1}H NMR (400 MHz, $CDCl_3$) δ (ppm): 7.72 (d, *J* = 8.0, 2H), 7.64 (d, *J* = 8.3, 2H), 7.48 (m, 2H), 7.30-7.40 (m, 3H), 5.75 (s, 1H), 3.37 (s, 1H, OH). ^{1}H NMR was in accordance with literature.[4]

1-(4-Methoxyphenyl)-3-phenylprop-2-yn-1-ol (1e). Following general procedure A, acetylene **7a** (472 mg, 4.62 mmol) in THF (10 mL) was reacted with LDA (3.2 mL, 2 M, 6.4 mmol). Addition of aldehyde **6d** (639 mg, 4.69 mmol) yielded propargyl alcohol **1e** (702 mg, 64 %) as a yellow solid after flash column chromatography (1:7 EtOAc:pentane). ^{1}H NMR (600 MHz, $CDCl_3$) δ (ppm): 7.54 (d, *J* = 8.5, 2H), 7.47 (m, 2H), 7.28-7.35 (m, 3H), 6.93 (d, *J* = 8.8, 2H), 5.65 (d, *J* = 6.1, 1H), 3.82 (s, 3H), 2.20 (d, *J* = 6.1, 1H, OH). ^{1}H NMR was in accordance with literature.[2]

3-(4-Methoxyphenyl)-1-phenylprop-2-yn-1-ol (1f). Following general procedure A, acetylene **7c** (609 mg, 4.61 mmol) in THF (10 mL) was reacted with LDA (3.5 mL, 2 M, 7.0 mmol). Addition of aldehyde **6a** (446 mg, 4.20 mmol) yielded propargyl alcohol **1f** (183mg, 18%) as a yellow solid after flash column chromatography (1:5 EtOAc:pentane). ^{1}H NMR (400 MHz, $CDCl_3$) δ (ppm): 7.62 (dm, J = 7.3, 2H), 7.39-7.42 (m, 4H), 7.34 (tt, J = 8.2, 2.0, 1H), 6.84 (d, J = 8.8, 2H), 5.68 (d, J = 6.1, 1H), 3.81 (s, 3H), 2.26 (d, J = 6.1, 1H, OH). ^{1}H NMR was in accordance with literature.[2]

1-Phenyl-3-(4-(trifluoromethyl)phenyl)prop-2-yn-1-ol (1g). Following general procedure A, acetylene **7d** (605 mg, 3.556 mmol) in THF (5 mL) was reacted with LDA (2.5 mL, 2 M, 5.0 mmol). Addition of aldehyde **6a** (345 mg, 3.25 mmol) yielded propargyl alcohol **1g** (367.4 mg, 41 %) as a white powder after flash column chromatography (1:10 EtOAc:pentane). ^{1}H NMR (600 MHz, $CDCl_3$) δ (ppm): 7.61 (m, 2H), 7.58 (s, 4H), 7.43 (m, 2H), 7.37 (m, 1H), 5.71 (d, J = 6.1), 2.27 (d, J = 6.2, OH). ^{1}H NMR was in accordance with literature.[2]

3-Mesityl-1-phenylprop-2-yn-1-ol (1h). Following general procedure A, acetylene **7b** (470 mg, 3.26 mmol) in THF (5 mL) was reacted with LDA (2.2 mL, 2 M, 4.34 mmol). Addition of aldehyde **6a** (230 mg, 2.17 mmol) yielded propargyl alcohol **1h** (329 mg, 61 %) as a light brown powder after flash column chromatography (1:13 EtOAc:pentane). ^{1}H NMR (600 MHz, $CDCl_3$) δ (ppm): 7.65 (d, J = 7.3, 2H), 7.40 (t, J = 7.5, 2H), 7.34 (t, J = 7.3, 1H), 6.86 (s, 2H), 5.77 (d, J = 6.3, 1H), 2.40 (s, 6 h, 2xMe), 2.27 (s, 3H, Me), 2.23 (d, J = 6.3, 1H, OH); ^{13}C NMR (150 MHz, $CDCl_3$) δ (ppm): 141.1, 140.4, 138.1 , 128.6, 128.4, 127.6 , 126.8, 119.1, 96.3, 84.7, 65.5, 21.30, 21.03. HRMS (ASAP) calcd for $C_{18}H_{17}$ [M-OH]$^{+}$ 233.1330, obsd 233.1328.

1-Mesityl-3-(4-(trifluoromethyl)phenyl)prop-2-yn-1-ol (1i). Following general procedure A, acetylene **7d** (589 mg, 3.46 mmol) in THF (3 mL) was reacted with LDA (3.00 mL, 2M, 6.00 mmol). Addition of aldehyde **6b** (465 mg, 3.14 mmol) yielded propargyl alcohol **1i** (528 mg, 53 %) as a green solid after flash column chromatography (1:10 EtOAc:pentane). ^{1}H NMR (600 MHz, $CDCl_3$) δ (ppm): 7.55 (d, J = 8.4, 2H), 7.51 (d, J = 8.3, 2H), 6.89 (s, 2H), 6.13 (d, J = 3.3, 1H), 2.55 (s, 6 h, 2xMe), 2.27 (s, 3H, Me), 2.08 (s, 1H, OH); ^{13}C NMR (150 MHz, CDCl3) δ (ppm): 138.2, 136.6, 133.2, 131.9, 130.12, 130.11 (q, J = 32.6), 126.6, 125.2 (q, J = 3.7), 123.9 (q, J = 272.5), 91.3, 84.3, 60.7, 20.9, 20.3. HRMS (ASAP) calcd for $C_{19}H_{17}OF_3$ [M*]$^{+}$ 318.1231, obsd 318.1229.

Preparation of C3 substitution products (2):

(3-Mesitylprop-1-yne-1,3-diyl)dibenzene (2a). Propargyl alcohol **1a** (42.2 mg, 0.203 mmol), mesitylene (146 mg, 1.216 mmol) and $AuBr_3$ (5.8 mg, 0.013 mmol) were stirred in ACN (3 mL) at 85 °C for 1.5 h. Water (10 mL) was added, and the solution extracted into diethyl ether (3x10 mL), dried over Na_2SO_4 and the solvent removed *in vacuo*. Purification by flash column chromatography (1:100 EtOAc:pentane) gave a 1:3 mixture of alkyne **2a** and indene **4a** (51.3 mg total). ^{1}H NMR values are extracted from a 1:3 mixture of alkyne **2a** / indene **4a**. See SI for the combined (**2a** + **4a**) ^{1}H NMR spectrum. ^{1}H NMR (400 MHz, $CDCl_3$) δ (ppm): 7.46 (m, 2H), 7.38 (d, J = 7.7, 2H), 7.25-7.31 (m, 4H), 7.20 (m, 2H), 6.88 (s, 2H), 5.72 (s, 1H), 2.29 (s, 6 h), 2.28 (s, 3H). ^{1}H NMR was in accordance with literature.[5]

1,3,5-Trimethyl-2-(3-phenyl-1-(4-(trifluoromethyl)phenyl)prop-2-yn-1-yl)benzene (2d). Propargyl alcohol **1d** (19.0 mg, 0.069 mmol), mesitylene (49.5 mg, 0.4122 mmol) and $AuBr_3$ (1.5 mg, 0.003 mmol) were stirred in F_3-EtOH at r.t. for 15 min. H_2O (5 mL) was added and the solution extracted into diethyl ether (3x10 mL), dried over Na_2SO_4 and solvent removed *in vacuo*. Through flash column chromatography (1:100 EtOAc:pentane) alkyne **2d** was isolated as a minor product (1.2 mg, 5 %). ^{1}H NMR (600 MHz, $CDCl_3$) δ (ppm): 7.54 (m, 2H), 7.49 (m, 2H), 7.47 (m, 2H), 7.31 (m, 3H), 6.90 (s, 2H), 5.73 (s, 1H), 2.25-2.32 (m, 6 h). Insufficient amounts for ^{13}C NMR or HRMS.

1,3,5-Trimethyl-2-(1-phenyl-3-(4-(trifluoromethyl)phenyl)prop-2-yn-1-yl)benzene (2g). Propargyl alcohol **1g** (20 mg, 0.072 mmol), mesitylene (13.1 mg, 0.109 mmol) and $AuBr_3$ (1.6 mg, 0.004 mmol) were stirred in $MeNO_2$ at r.t. for 15 min. H_2O (5 mL) was added and the solution extracted into diethyl ether (3x10 mL), dried over Na_2SO_4 and solvent removed *in vacuo*. Through flash column chromatography (petroleum ether) a 3:1 mixture of alkyne **2e** and allene **3e** were collected (total 11.7 mg). See SI for the combined (**2e** + **3e**) ^{1}H NMR spectrum.

(3-(4-Methoxyphenyl)prop-1-yne-1,3-diyl)dibenzene (2l). Propargyl alcohol **1a** (48.2 mg, 0.231 mmol) was stirred with anisole (146.3 mg, 1.353 mmol) in F_3-EtOH. $AuBr_3$ (4.8 mg, 0.011 mmol) was added, and the mixture heated to 85 °C and stirred for 1.5 h before H_2O (5 mL) was added. The solution was extracted with diethyl ether (3x10 mL) and dried over Na_2SO_4. Removal of the solvent *in vacuo* and purification by flash column chromatography (1:24 EtOAc:pentane) gave alkyne **2i** (53.1 mg, 77%) as a yellow oil. ^{1}H NMR (600 MHz,

$CDCl_3$) δ (ppm): 7.44-7.48 (m, 2H), 7.40-7.44 (m, 2H), 7.25-7.36 (m, 7H), 7.18-7.24 (m, 2H), 6.82-6.87 (m, 2H), 5.16 (s, 1H), 3.75 (s, 3H, OMe). ^{1}H NMR was in accordance with literature.[6]

(3-(2,4,6-Trimethoxyphenyl)prop-1-yne-1,3-diyl)dibenzene (2m). Propargyl alcohol **1a** (9.5 mg, 0.046 mmol) was stirred with 1,3,5-trimethoxybenzene (41.8 mg, 0.275 mmol) in F_3-EtOH. $AuBr_3$ (1.1 mg, 0.002 mmol) was added, and the mixture stirred at r.t. for 15 min before H_2O (5 mL) was added. The solution was extracted with diethyl ether (3x10 mL) and dried over Na_2SO_4. Removal of the solvent *in vacuo* and purification by flash column chromatography (1:30 EtOAc:pentane) gave alkyne **2j** (8.5 mg, 52 %) as a yellow oil. ^{1}H NMR (600 MHz, $CDCl_3$) δ (ppm): 7.50 (d, *J* = 7.8, 2H), 7.43-7.48 (m, 2H), 7.22-7.48 (m, 5H), 7.15 (t, *J* = 7.3, 1H), 6.15 (s, 2H), 5.86 (s, 1H), 3.80 (s, 6 h, 2xOMe), 3.78 (s, 3H, OMe). ^{1}H NMR was in accordance with literature.[7]

(3-(2,2,2-Trifluoroethoxy)prop-1-yne-1,3-diyl)dibenzene (2a$^{CF3-EtO}$). Propargyl alcohol **1a** (23.9 mg, 0.115 mmol) was stirred with 1,3,5-triisopropylbenzene (140.4 mg, 0.687 mmol) and $AuBr_3$ (2.5 mg, 0.006 mmol) in F_3-EtOH (1 mL). The solution was stirred at r.t. for 15 min before addition of water (5 mL), extraction into diethyl ether (3x10 mL) and drying over Na_2SO_4. Removal of solvent *in vacuo* and purification by flash column chromatography (1:25 EtOAc:pentane) yielded alkyne **2a$^{CF3-EtO}$** (7.7 mg, 23 %). ^{1}H NMR (600 MHz, $CDCl_3$) δ (ppm): 7.60 (m, 2H), 7.50 (m, 2H), 7.42 (m, 2H), 7.38 (m, 1H), 7.36 (m, 1H), 7.34 (m, 2H), 5.65 (s, 1H), 4.06 (dq, *J* = 12.0, 8.6), 3.98 (dq, *J* = 12.0, 8.8, 1H); ^{13}C NMR (150 MHz, CDCl3) δ (ppm): 137.0, 131.9, 128.99, 128.98, 128.7, 128.4, 127.6, 124.05 (q, *J* = 279.0), 121.9, 89.2, 84.7, 72.8, 64.6 (q, *J* = 34.6). HRMS (ASAP) calcd for $C_{17}H_{13}OF_3$ $[M*]^+$ 290.0918, obsd 290.0920.

3-Ethoxyprop-1-yne-1,3-diyl)dibenzene (2a^{OEt}). Propargyl alcohol **1a** (39.5 mg, 0.190 mmol) was stirred with mesitylene (137 mg, 1.14 mmol) in EtOH (4 mL) and $AuBr_3$ (4.0 mg, 0.009 mmol) was added. The solution was heated to 60 °C and stirred for 1.5 h. Water (5 mL) was added and the solution extracted into diethyl ether (3x10 mL) and dried over Na_2SO_4. Removal of solvent *in vacuo* and purification by flash column chromatography (1:20 EtOAc:pentane) yielded alkyne **2a^{OEt}** (25.5 mg, 57 %) as a faint yellow oil. ^{1}H NMR (400 MHz, $CDCl_3$) δ (ppm): 7.58 (d, *J* = 7.6, 2H), 7.47 (m, 2H), 7.39 (t, *J* = 7.6, 2H), 7.27-7.36 (m, 4H), 5.39 (s, 1H), 3.80 (dq, *J* = 8.8, 7.1, 1H), 3.63 (dq, *J* = 8.9, 7.1, 1H), 1.29 (t, *J* = 7.0, 3H). ^{1}H NMR was in accordance with literature.[8]

1-Methoxy-4-(3-phenyl-1-(2,2,2-trifluoroethoxy)prop-2-yn-1-yl)benzene (2e$^{CF3-EtO}$). Propargyl alcohol **1e** (16.5 mg, 0.069 mmol) was stirred with mesitylene (49.5 mg, 0.412 mmol) in F_3-EtOH. $AuBr_3$ (1.5 mg, 0.003 mmol) was added, and the mixture stirred at r.t. for 15 min before H_2O (5 mL) was added. The solution was extracted with diethyl ether (3x10 mL) and dried over Na_2SO_4. Removal of the solvent *in vacuo* gave a complex product mixture, including product **2e$^{CF3-EtO}$**. Purification by flash column chromatography (1:100 EtOAc:pentane) gave alkyne **2e$^{CF3-EtO}$** (6.2 mg, 28 %) as a yellow oil. ^{1}H NMR (600 MHz, $CDCl_3$) δ (ppm): 7.47-7.54 (m, 4H), 7.31-7.39 (m, 3H), 6.94 (d, *J* = 8.7, 2H), 5.61 (s, 1H), 4.03 (dq, *J* = 12.0, 8.6, 1H), 3.93 (dq, *J* =11.8, 8.9, 1H, CH_2), 3.83 (s, 3H, OMe); ^{13}C NMR (150 MHz, $CDCl_3$) δ (ppm): 160.2, 131.9, 129.15, 129.12, 128.9, 128.4, 124.1 (q, *J* = 279.5, CF_3), 122.0, 114.0, 89.0, 84.9, 72.4, 64.4 (q, *J* = 34.4, CH_2), 55.4 (OMe). HRMS (ASAP) calcd for $C_{18}H_{15}O_2F_3$ $[M*]^+$ 320.1024, obsd 320.1019.

Preparation of 1,1,3-triarylallenes (3); General procedure B: Propargyl alcohol **1** (1 eq.) and an aromatic nucleophile (1 - 6 eq.) were dissolved in either F_3-EtOH or $MeNO_2$ (1 mL). A solution of $AuBr_3$ (0.05 eq.) in the same solvent (1 mL) was added, and the solution stirred at r.t. for 15 min. H_2O (5 mL), a few drops of NEt_3 and diethyl ether (5 mL) were added and the layers separated. The aqueous layer was extracted with diethyl ether (3x10 mL), and the combined organic layers were dried over Na_2SO_4, followed by removal of solvent *in vacuo*. Purification by flash column chromatography (1:200 EtOAc:pentane) yielded allenes **3**.

(1-Mesitylpropa-1,2-diene-1,3-diyl)dibenzene (3a). Following general procedure B, propargyl alcohol **1a** (45.7 mg, 0.219 mmol) was reacted with mesitylene (121 mg, 1.006 mmol) in F_3-EtOH, in the presence of $AuBr_3$ (4.3 mg, 0.010 mmol). Workup and purification by crystallization from petroleum ether yielded allene **3a** (26.5 mg, 40 %) as a white solid. ^{1}H NMR (600 MHz, $CDCl_3$) δ (ppm): 7.38 (m, 2H), 7.16-7.34 (m, 8H), 6.94 (s, 2H), 6.59 (s, 1H, =C=C-*H*), 2.31 (s, 3H), 2.25 (s, 6 h). ^{1}H NMR was in accordance with literature.[9]

2-(3-(2,6-Dimethylphenyl)-1-phenylpropa-1,2-dien-1-yl)-1,3,5-trimethylbenzene (3b). Following general procedure B, propargyl alcohol **1b** (20.1 mg, 0.085 mmol) was reacted with mesitylene (15.3 mg, 0.127 mmol) in the presence of $AuBr_3$ (1.9 mg, 0.004 mmol) in $MeNO_2$. Workup and purification gave pure allene **3b** (9.9 mg, 34 %). ^{1}H NMR (600 MHz, $CDCl_3$) δ (ppm): 7.27 (dd, *J* = 7.6, 7.6, 2H), 7.23 (d, *J* = 8.3, 2H), 7.19 (t, *J* = 7.1, 1H), 6.99-7.06 (m, 3H), 6.92 (s, 2H), 6.71 (s, 1H, =C=C-*H*), 2.35 (s, 6 h, 2xMe), 2.31 (s, 3H, Me), 2.22 (s, 3H, Me), 2.07 (s, 3H, Me); ^{13}C NMR (150 MHz, $CDCl_3$) δ (ppm): 205.8 (=*C*=), 137.2, 137.1, 137.0, 136.6, 136.3, 131.7, 131.4, 128.51, 128.48, 128.1, 126.74, 126.70, 126.3, 106.4, 92.2, 21.3 (2xMe), 21.1 (Me), 20.3 (2xMe). HRMS (ASAP) calcd for $C_{26\,h27}$ $[M+H]^+$ 339.2113, obsd 339.2108.

2,2'-(1-Phenylpropa-1,2-diene-1,3-diyl)bis(1,3,5-trimethylbenzene) (3c). Following general procedure B, propargyl alcohol **1c** (20.2 mg, 0.080 mmol) was reacted with mesitylene (14.4 mg, 0.120 mmol) in the presence of $AuBr_3$ (1.7 mg, 0.004 mmol) in $MeNO_2$. Workup and purification gave allene **3c** (10.5 mg, 37 %). ^{1}H NMR (600 MHz, $CDCl_3$) δ (ppm): 7.27 (dd, *J* = 7.5, 7.5. 2H), 7.22 (d, *J* = 7.2, 2H), 7.18 (dd, *J* = 7.1, 7.1, 1H), 6.92 (bs, 2H), 6.85 (s, 2H), 6.70 (s, 1H, =C=C-*H*), 2.32 (s, 6 h, 2xMe), 2.31 (s, 3H, Me), 2.26 (s, 3H, Me), 2.21 (s, 3H, Me), 2.07 (s, 3H, Me); ^{13}C NMR (150 MHz, $CDCl_3$) δ (ppm): 205.8 (=*C*=), 137.2, 137.0, 136.9, 136.5, 136.3, 131.9, 129.0, 128.49, 128.45, 128.3, 126.6, 126.3, 106.2, 92.1, 21.2 (2xMe), 21.1 (Me), 20.9 (Me), 20.3 (2xMe). HRMS (ASAP) calcd for $C_{27}H_{29}$ $[M+H]^+$ 353.2269, obsd 353.2263.

1,3,5-Trimethyl-2-(1-phenyl-3-(4-(trifluoromethyl)phenyl)propa-1,2-dien-1-yl)benzene (3d). Following general procedure B, propargyl alcohol **1d** (19.2 mg, 0.069 mmol) was reacted with mesitylene (49.5 mg, 0.412 mmol) in the presence of $AuBr_3$ (1.5 mg, 0.003 mmol) in F_3-EtOH. Workup and purification gave pure allene **3d** (4.9 mg, 19 %). ^{1}H NMR (600 MHz, $CDCl_3$) δ (ppm): 7.55 (d, *J* = 8.2, 2H), 7.46 (d, *J* = 8.1, 2H), 7.29 (m, 2H), 7.20-7.25 (m, 3H), 6.95 (s, 2H) 6.61 (s, 1H, =C=C-*H*), 2.32 (s, 3H, Me), 2.25 (s, 6 h, 2xMe); ^{13}C NMR (150 MHz, $CDCl_3$) δ (ppm): 206.8 (=*C*=), 138.2, 137.4, 136.8, 134.9, 131.1, 126.0 (q, *J* = 32.2, *C*- CF_3), 128.8, 128.6, 127.5, 127.2, 126.3, 125.7 (q, *J* = 3.7, C3'''), 124.2 (q, *J* = 271.8, CF_3), 110.2, 96.1, 21.1 (Me), 20.5 (2xMe). HRMS (ESI) calcd for $C_{25}H_{22}F_3$ $[M+H]^+$ 379.1674, obsd 379.1667.

1,3,5-Trimethyl-2-(3-phenyl-1-(4-(trifluoromethyl)phenyl)propa-1,2-dien-1-yl)benzene (3g). Following general procedure B, propargyl alcohol **1g** (20.1 mg, 0.072 mmol) was reacted with mesitylene (13.1 mg, 0.109 mmol) in the presence of $AuBr_3$ (1.7 mg, 0.004 mmol) in $MeNO_2$. Workup and flash column chromatography gave a 1:4 mixture of alkyne **2g** and allene **3g** (8.7 mg total). See SI for the combined (**2e** + **3e**) ^{1}H NMR spectrum.

2,2'-(1-(4-(Trifluoromethyl)phenyl)propa-1,2-diene-1,3-diyl)bis(1,3,5-trimethylbenzene) (3i). Following general procedure B, propargyl alcohol **1i** (15.0 mg, 0.047 mmol) was reacted with mesitylene (8.5 mg, 0.071 mmol) in the presence of $AuBr_3$ (1.1 mg, 0.002 mmol) in $MeNO_2$. Workup and purification gave pure allene **3i** (8.0 mg, 40 %) as an orange oil. ^{1}H NMR (600 MHz, $CDCl_3$) δ (ppm): 7.51 (d, *J* = 8.2, 2H), 7.32 (d, *J* = 8.2, 2H, H), 6.94 (s, 2H, H), 6.86 (s, 2H), 6.76 (s, 1H, , =C=C-*H*), 2.32 (s, 3H, Me), 2.31 (s, 6 h, 2xMe), 2.27 (s, 3H, Me), 2.20 (s, 3H, Me), 2.04 (s, 3H, Me); ^{13}C NMR (150 MHz, $CDCl_3$) δ (ppm): 206.5 (=C=), 140.5, 137.4, 137.3, 136.9, 136.7, 136.5, 131.1, 129.1, 128.7, 128.6 (*J* = 32.3, *C*- CF_3),127.7, 126.4, 125.4 (*J* = 3.7), 124.3 (*J* = 271.8, CF_3), 105.6, 92.5, 21.2 (2xMe), 21.1 (Me), 20.9 (Me), 20.3 (2xMe). HRMS (ASAP) calcd for $C_{28}H_{28}F_3$ $[M+H]^+$ 421.2143, obsd 421.2137.

(1-(2,3,4,5,6-Pentamethylphenyl)propa-1,2-diene-1,3-diyl)dibenzene (3j). Following general procedure B, propargyl alcohol **1j** (14.5 mg, 0.070 mmol) was reacted with pentamethylbenzene (15.6 mg, 0.105 mmol) in the presence of $AuBr_3$ (1.7 mg, 0.004 mmol) in F_3-EtOH. Extraction into diethyl ether (3 x 10 mL) gave pure allene **3j** (23.3 mg, 99 %) without the need for further purification. ^{1}H NMR (400 MHz, $CDCl_3$) δ (ppm): 7.36 (d, *J* = 7.6, 2H), 7.23-7.31 (m, 6 h), 7.19 (m, 2H), 6.56 (s, 1H, =C=C-*H*), 2.31 (s, 3H, Me), 2.27 (s, 3H, Me), 2.25 (s, 3H, Me), 2.22 (s, 6 h, 2xMe); ^{13}C NMR (150 MHz, $CDCl_3$) δ (ppm): 206.1 (=*C*=), 136.1, 134.51, 134.45, 133.2, 132.75, 132.74, 132.5, 132.3, 132.2, 128.63, 128.60, 127.2, 127.1, 127.0, 126.4, 111.2, 96.9, 18.8, 17.8, 16.8, 16.7, 16.6. HRMS (ASAP) calcd for $C_{26\,h26}F_3$ $[M^*]^+$ 338.2035 , obsd 338.2034.

Preparation of diarylindenes (4); General procedure C: Propargyl alcohol **1** (1 eq.) and aromatic nucleophile (1 - 6 eq.) were dissolved in either F_3-EtOH or $MeNO_2$ (1 mL). A solution of $AuBr_3$ (0.05 eq.) in the same solvent (1 mL) was added, and the solution stirred at 80 °C for 1.5 h. H_2O (5 mL), a few drops of NEt_3 and diethyl ether (5 mL) were added, and the layers separated. The aqueous layer was extracted with diethyl ether (3x5 mL), and the combined organic layers were dried over Na_2SO_4, followed by removal of solvent *in vacuo*. Purification by flash column chromatography (1:200 EtOAc:pentane) yielded indenes **4**.

3-Mesityl-1-phenyl-1H-indene (4a). Following general procedure C, propargyl alcohol **1a** (52.9 mg, 0.254 mmol) was reacted with mesitylene (183.2 mg, 1.524 mmol) in the presence of catalytic $AuBr_3$ (5.6 mg, 0.013 mmol) in F_3-EtOH. Workup and purification gave indene **4a** (72.7 mg, 92 %) as a colourless solid. ^{1}H NMR (600 MHz, $CDCl_3$) δ (ppm): 7.28-7.32 (m, 3H), 7.24 (m, 1H), 7.18-7.22 (m, 3H), 7.17 (m, 1H), 6.96-7.00 (m, 2H), 6.93 (d, *J* = 7.4, 1H), 6.37 (d, *J* = 2.0, 1H), 4.78 (d, *J* = 1.9, 1H), 2.35 (s, 3H, Me), 2.21 (s, 3H, Me), 2.13 (s, 3H, Me). ^{1}H NMR was in accordance with literature. [9]

1-(2,6-Dimethylphenyl)-3-mesityl-1H-indene (4b). Following general procedure C, propargyl alcohol **1b** (19.8 mg, 0.085 mmol) was reacted with mesitylene (15,3 mg, 0.127 mmo) in the presence of catalytic $AuBr_3$ (2.0 mg, 0.005 mmol) in $MeNO_2$. Workup and purification gave pure indene **4b** (12.8 mg, 45 %) as a colourless solid. ^{1}H NMR (600 MHz, $CDCl_3$) δ (ppm): 7.23 (t, *J* = 7.3), 7.14-7.215 (m, 3H), 7.09 (t, *J* = 7.5, 1H), 7.01 (d, *J* = 7.5, 1H), 6.97 (s, 2H), 6.89 (d, *J* = 7.4, 1H), 6.39 (d, *J* = 2, 1H), 5.17 (s, 1H), 2.66 (s, 3H, Me), 2.35 (s, 3H, Me), 2.18

(s, 3H, Me), 2.11 (s, 3H, Me), 1.65 (s, 3H, Me); ^{13}C NMR (150 MHz, $CDCl_3$) δ (ppm): 147.1, 144.4, 142.9, 138.2, 137.6, 137.2, 137.0, 136.9, 135.8, 135.5 132.0, 129.2, 128.3 (2xC) 128.2, 126.7, 126.3, 125.2, 122.9, 120.6, 52.3, 21.9 (Me), 21.1 (Me), 20.7 (Me), 20.4 (Me), 19.0 (Me). HRMS (ASAP) calcd for $C_{26\ h26}$ [M*]$^+$ 338.2035, obsd 338.2034.

1,3-Dimesityl-1H-indene (4c). Following general procedure C, propargyl alcohol **1c** (20.3 mg, 0.080 mmol) was reacted with mesitylene (14.4 mg, 0.120 mmol) in the presence of catalytic $AuBr_3$ (1.7 mg, 0.004 mmol) in $MeNO_2$. Workup and purification gave the pure indene **4c** (14.2 mg, 50 %) as a colourless solid. ^{1}H NMR (400 MHz, $CDCl_3$) δ (ppm): 7.12-7.24 (m, 3H), 6.94-7.03 (m, 4H), 6.73 (s, 1H), 6.37 (d, J = 2.0, 1H), 5.13 (s, 1H), 2.62 (s, 3H, Me), 2.35 (s, 3H, Me), 2.28 (s, 3H, Me), 2.17 (s, 3H, Me), 2.11 (s, 3H, Me), 1.61 (s, 3H, Me). ^{1}H NMR was in accordance with literature.[10]

3-Mesityl-1-(4-(trifluoromethyl)phenyl)-1H-indene (4d). Following general procedure C, propargyl alcohol **1d** (52.4 mg, 0.190 mmol) was reacted with mesitylene (114 mg, 0.948 mmol) in the presence of $AuBr_3$ (4.1 mg, 0.010 mmol) in F_3-EtOH. After 90 min reaction time, an additional 4.1 mg $AuBr_3$ was added and the reaction stirred at 80 °C for a further 3.5 h. Flash column chromatography (40:1 pentane:DCM) gave **4d** in a 2:1 mix of **4d + 4d-Br** (29 +14% yield; 33.0 mg mix, 20.6 mg **4d** + 12.4 mg **4d-Br**). ^{1}H NMR (600 MHz, $CDCl_3$) δ (ppm): 7.58 (d, J = 8.0 Hz, 2H), 7.33 (d, J) 8.0 Hz, 2H), 7.30-7.31 (m, 1H), 7.19-7.23 (m, 2H), 7.02 (s, 1H), 7.01 (s, 1H), 6.98 (dt, J 7.4, 0.9 Hz, 1H), 6.37 (d, J = 2.1 Hz, 1H), 4.85 (d, J = 2.1 Hz, 1H), 2.37 (s, 3H), 2.22 (s, 3H), 2.15 (s, 3H). ^{13}C NMR (150 MHz, $CDCl_3$) δ (ppm): 147.9, 144.7, 144.6, 144.4, 137.2, 136.7, 136.6, 136.1, 131.7, 129.3 (q, J = 32.5 Hz), 129.0, 128.3, 128.3, 127.3, 127.1, 125.9, 125.9 (q, J = 3.8 Hz), 124.4 (q, J = 271.7), 124.1, 120.7, 55.7, 21.2, 20.7, 20.3. HRMS (ESI) calcd for $C_{25}H_{21}F_3$ [M*]$^+$ 378.1595, obsd 379.1601. HRMS (ASAP) calcd for $C_{25}H_{21}F_3$ [M]$^+$ 378.1595, obsd 378.1601.

3-(2,3,4,5,6-Pentamethylphenyl)-1-phenyl-1H-indene (4j). Following general procedure C, propargyl alcohol **1a** (56.8 mg, 0.273 mmol) was reacted with pentamethylbenzene (40.4 mg, 0.273 mmol) in the presence of catalytic $AuBr_3$ (6.0 mg, 0.014 mmol) in F_3-EtOH. Workup and purification by flash column chromatography (200:1 pentane:DCM) gave **4j** (51.7 mg, 56%) as a white solid. ^{1}H NMR (400 MHz, $CDCl_3$) δ (ppm): 7.17-7.33 (m, 8H), 6.92 (m, 1H), 6.34 (d, J = 2.1, 1H), 4.79 (d, J = 1.8, 1H), 2.32 (s, 3H), 2.28 (s, 3H), 2.28 (s, 3H), 2.19 (s, 3H), 2.10 (s, 3H). ^{1}H NMR was in accordance with literature.[9]

1-Phenyl-3-(2,4,6-triisopropylphenyl)-1H-indene (4k). Following general procedure C, propargyl alcohol **1a** (23.9 mg, 0.115 mmol) was reacted with 1,3,5-triisopropylbenzene (140.4 mg, 0.687 mmol) in the presence of catalytic $AuBr_3$ (2.5 mg, 0.006 mmol) in F_3-EtOH. Workup and purification gave indene **4k** (6.8 mg, 15 %) as a colourless solid. ^{1}H NMR (600 MHz, $CDCl_3$) δ (ppm): 7.27-7.33 (m, 3H), 7.18-7.25 (m, 4H), 7.16 (ddd, J = 7.4, 7.4, 1.1, 1H), 7.10 (m, 2H), 6.96 (d, J = 7.3, 1H), 6.40 (d, J = 2.0, 1H), 4.78 (d, J = 1.5, 1H), 3.02 (hept, J = 6.9, 1H), 2.96 (hept, J = 6.9, 1H), 2.85 (hept, J = 6.9, 1H), 1.32 (d, J = 6.9, 6 h), 1.15 (d, J = 6.8, 9H), 1.09 (d, J = 6.9, 3H). ^{1}H NMR was in accordance with literature.[9]

Preparation of 2-halo-indenes (4-X); General procedure D: Propargyl alcohol **1a** (1 eq) and aromatic nucleophile (1-6 eq) were dissolved in 1 mL F_3-EtOH and $AuBr_3$ (0.05 eq) added. The resulting mixture was stirred for 15 min at room temperature. After conversion to the allene **3a/j** was complete, NXS (1.1 eq) was added, and the reaction was stirred for 15-120 min. After completion of the reaction, diethyl ether was added and the organic phase washed with sat. $NaHCO_3$, followed by brine and drying over anhydrous Na_2SO_4 and evaporation of the solvent *in vacuo*. The crude products were then purified by column chromatography.

2-Bromo-3-mesityl-1-phenyl-1H-indene (4a-Br). Following the general procedure D propargyl alcohol **1a** (46.7 mg, 0.224 mmol) was reacted with mesitylene (135 mg, 1.12 mmol) in the presence of $AuBr_3$ (4.9 mg, 0.011 mmol) for 120 min. NBS (43.9 mg, 0.247 mmol) was then added and the mixture stirred for a further 120 min. Purification by column chromatography (pentane) gave product **4a-Br** as a yellow oil in 56% yield (49.3 mg). ^{1}H NMR (600 MHz, $CDCl_3$) δ (ppm): 7.30-7.33 (m, 2H), 7.25-7.29 (m, 1H), 7.11-7.20 (m, 5H), 6.97 (s, 2H), 6.87 (dt, J =7.2, 0.6 Hz, 1H), 4.76 (s, 1H), 2.34 (s, 3H), 2.16 (s, 3H), 2.11 (s, 3H). ^{13}C NMR (150 MHz, $CDCl_3$) δ (ppm): 147.3, 143.5, 143.4, 138.3, 137.8, 136.7, 136.5, 129.0, 128.6, 128.4, 127.6, 127.4, 125.9, 124.1, 120.0, 60.8, 21.4, 20.2, 19.9. HRMS (ASAP) calcd for $C_{24\ h22}Br$ [M+H]$^+$ 389.0905, obsd 389.0907.

2-bromo-3-mesityl-1-(4-(trifluoromethyl)phenyl)-1H-indene (4d-Br). Following the general procedure D propargyl alcohol **1d** (37.3 mg, 0.135 mmol) was reacted with mesitylene (81.1 mg, 0.675 mmol) in the presence of $AuBr_3$ (3.0 mg, 0.0068 mmol) for 15 min. NBS (26.4 mg, 0.149 mmol) was then added and the mixture stirred for a further 3 h. Aqueous workup resulted in a 1:1 mixture of **2d** and **4d-Br** that could not be separated chromatographically. A sample was acquired for analysis by addition of 2 eq of *m*-CPBA to a 3:1 mixture of **4d** and **4d-Br** for 3 h. Purification by column chromatography (pentane) resulted in a pure sample of **4d-Br** for

characterization. ^{1}H NMR (600 MHz, $CDCl_3$) δ (ppm): 7.61 (d, J = 8.0 Hz, 2H), 7.28 (d, J = 8.0 Hz, 2H), 7.22 (td, J = 7.7, 2.7 Hz, 1H), 7.17-7.20 (m, 2H), 7.00 (s, 2H), 6.91 (dt, J = 7.4, 0.9 Hz, 1H), 4.83 (s, 1H), 2.36 (s, 3H), 2.16 (s, 3H), 2.12 (s, 3H). ^{13}C NMR (150 MHz, $CDCl_3$) d(ppm) 146.5, 144.2, 143.5, 142.7, 138.0, 136.6, 136.4, 129.9 (q, J = 32.2 Hz), 129.5, 129.0, 128.5, 128.4, 127.8, 126.0, 126.0 (q, J = 3.7 Hz), 124.3 (q, J = 271.7 Hz), 124.0, 120.3, 60.3, 21.4, 20.1, 19.9. HRMS (ASAP) calcd for $C_{25}H_{20}BrF_3$ $[M*]^+$ 456.0700 obsd 427.0715

2-Iodo-3-mesityl-1-phenyl-1H-indene (4a-I). Following the general procedure D propargyl alcohol **1a** (58.7 mg, 0.282 mmol) was reacted with mesitylene (203 mg, 1.69 mmol) in the presence of $AuBr_3$ (6.2 mg, 0.014 mmol) for 15 min. NIS (69.8 mg, 0.321 mmol) was then added and the mixture stirred for a further 15 min. Purification by column chromatography (200:1 pentane:Et_2O) gave product **4a-I** in 68% yield (84.1 mg). ^{1}H NMR (600 MHz, $CDCl_3$) δ (ppm): 7.32-7.35 (m, 2H), 7.27-7.31 (m, 2H), 7.22-7.23 (m, 1H), 7.15-7.17 (m, 1H), 7.11-7.14 (m, 3H), 6.99 (s, 2H), 6.88-6.89 (m, 1H), 4.74 (s, 1H), 2.36 (s, 3H), 2.16 (s, 3H), 2.11 (s, 3H). ^{13}C NMR (150 MHz, $CDCl_3$) δ (ppm): 150.5, 149.3, 143.8, 139.0, 137.8, 136.4, 136.2, 131.8, 129.0, 128.8, 128.4, 128.4, 127.5, 127.3, 127.1, 125.8, 124.2, 119.9, 107.5, 63.5, 21.4, 20.2, 20.0. HRMS (ASAP) calcd for $C_{24}\ _{h22}I$ $[M+H]^+$ 437.0770, obsd 437.0766.

2-Iodo-3-(2,3,4,5,6-pentamethylphenyl)-1-phenyl-1H-indene (4j-I). Following the general procedure D propargyl alcohol **1a** (55.1 mg, 0.265 mmol) was reacted with pentamethylbenzene (39.2 mg, 0.265 mmol) in the presence of $AuBr_3$ (5.8 mg, 0.013 mmol) for 15 min. NIS (65.5 mg, 0.292 mmol) was then added and the mixture stirred for a further 15 min. Purification by column chromatography (250:1 pentane:Et_2O) gave product **4j-I** as a white solid in 82% yield (101 mg). ^{1}H NMR (600 MHz, $CDCl_3$) δ (ppm): 7.35-7.37 (m, 2H), 7.32 (tt, J = 7.2, 1.5 Hz, 1H), 7.24-7.25 (m, 1H), 7.15-7.18 (m, 3H), 7.13 (td, J = 7.4, 1.5 Hz, 1H), 6.88-6.89 (m, 1H), 4.77 (s, 1H), 2.33 (s, 3H), 2.29 (s, 6 h), 2.13 (s, 3H), 2.07 (s, 3H). ^{13}C NMR (150 MHz, $CDCl_3$) d(ppm) 152.2, 149.2, 144.5, 139.2, 135.0, 132.6, 132.6, 132.3, 131.7, 131.4, 128.9, 128.9, 127.5, 127.2, 125.7, 124.1, 120.3, 107.7, 63.5, 17.8, 17.6, 17.1, 16.8, 16.8. HRMS (ASAP) calcd for $C_{26\ h26}I$ $[M+H]^+$ 465.1079, obsd 465.1088.

Preparation of tetra-arylallyl compounds (5); General procedure E: Propargyl alcohol **1a** (1 eq) and mesitylene (6 eq) were dissolved in 1 mL F_3-EtOH and $AuBr_3$ (0.05 eq) added. The resulting mixture was stirred for 15 min at room temperature. After conversion to the allene **3a** was complete, the aryl nucleophile (1-10 eq) was added, and the reaction mixture was stirred at appropriate temperature and time. After completion of the reaction, diethyl ether was added and the organic phase washed with water, followed by brine and drying over anhydrous Na_2SO_4 and evaporation of the solvent *in vacuo*. The crude products were then purified by column chromatography.

3-(3-Mesityl-1,3-diphenylallyl)-1H-indole (5a). Following the general procedure E, propargyl alcohol **1a** (78.1 mg, 0.375 mmol) was reacted with mesitylene (270 mg, 2.25 mmol) in the presence of $AuBr_3$ (8.2 mg, 0.019 mmol) for 15 min. Indole (43.9 mg, 0.375 mmol) was added and the reaction was complete after 6 h at 80 °C. Purification by column chromatography (30:1 pentane:Et_2O) gave product **5a** as white solid in 37% yield (57.9 mg) as both the pure *E* isomer and a 10:1 mixture of *E/Z* stereoisomers.

Main **5a** *E*-isomer (pure): ^{1}H NMR (600 MHz, $CDCl_3$) δ (ppm) 8.02 (s, 1H), 7.38 (dt, J = 8.1, 0.9 Hz, 1H), 7.21-7.27 (m, 9H), 7.16-7.20 (m, 3H), 7.11 (dd, J = 2.3, 1.0 Hz, 1H), 6.87 (s, 1H), 6.82 (s, 1H), 5.97 (d, J = 10.3 Hz, 1H), 5.51 (d, J = 10.4 Hz, 1H), 2.27 (s, 3H), 2.10 (s, 3H), 2.03 (s, 3H). ^{13}C NMR (150 MHz, $CDCl_3$) δ (ppm) 144.5, 140.5, 139.3, 138.2, 136.9, 136.6, 136.5, 136.5, 133.8, 129.0, 128.5, 128.4, 128.4, 128.2, 128.2, 127.1, 126.8, 126.3, 122.2, 120.2, 119.5, 119.4, 111.2, 42.8, 21.1, 20.7, 20.6. HRMS (ASAP) calcd for $C_{32}H_{29}N$ $[M*]^+$ 427.2300 obsd 427.2299

Minor **5a** *Z*-isomer (analyzed in a 10:1 mixture of *E/Z* isomers): ^{1}H NMR (600 MHz, $CDCl_3$) δ (ppm) 7.91 (s, 1H), 7.18-7.33 (m, 9H), 7.13-7.17 (m, 3H), 7.07 (dd, J = 2.4, 0.9 Hz, 1H), 6.97 (s, 1H), 6.95 (dd, J = 7.1, 0.8 Hz, 1H), 6.94 (d, J = 10.2 Hz, 1H), 6.91 (s, 1H), 4.73 (d, J = 10.2 Hz, 1H), 2.39 (s, 3H), 1.96 (s, 3H), 1.94 (s, 3H). ^{13}C NMR (150 MHz, $CDCl_3$) δ (ppm) 144.2, 140.3, 138.7, 136.8, 136.7, 136.6, 135.3, 130.6, 128.3, 128.3, 127.1, 126.7, 126.1, 126.0, 121.9, 121.7, 119.2, 119.0, 111.0, 43.2, 21.2, 20.2, 20.1. HRMS (ASAP) calcd for $C_{32}H_{29}N$ $[M*]^+$ 427.2300 obsd 427.2299

2-(3-Mesityl-1,3-diphenylallyl)thiophene (5b). Following the general procedure E, propargyl alcohol **1a** (59.0 mg, 0.283 mmol) was reacted with mesitylene (204 mg, 1.70 mmol) in the presence of AuBr3 (6.2 mg, 0.014 mmol) for 15 min. Thiophene (119.0 mg, 1.42 mmol) was added and the reaction was complete after 24 h at room temperature. Purification by column chromatography (40:1 pentane:DCM) gave product **5b** as a yellow solid in 27% yield (30.2 mg) as both the pure *E* isomer and a 3:1 mixture of *E/Z* stereoisomers.

Main **5b** *E*-isomer (pure): 1H NMR (600 MHz, CDCl3) δ (ppm) 7.26-7.33 (m, 7H), 7.20-7.24 (m, 2H), 7.15-7.17 (m, 3H), 6.94 (s, 1H), 6.91 (dd, *J* = 5.3, 3.7 Hz, 1H), 6.90 (s, 1H), 6.83 (d, *J* = 10.2 Hz, 1H), 6.71-6.72 (m, 1H), 4.64 (d, *J* = 10.2 Hz, 1H), 2.36 (s, 3H), 2.05 (s, 3H), 1.76 (s, 3H). 13C NMR (150 MHz, CDCl3) δ (ppm) 148.8, 143.8, 139.9, 139.9, 136.8, 136.8, 136.7, 134.9, 129.9, 128.6, 128.6, 128.5, 128.4, 128.1, 127.5, 126.8, 126.7, 126.3, 124.5, 124.1, 46.7, 21.3, 20.1, 19.9. HRMS (ASAP) calcd for $C_{28}H_{26}S$ $[M^*]^+$ 394.1755, obsd 394.1762.

Minor **5b** *Z*-isomer (analyzed in a 3:1 mixture of *E/Z*-isomers): ^1H NMR (600 MHz, CDCl3) δ(ppm) 7.20-7.32 (m, 11H), 6.98 (dd, *J* = 5.1, 3.4 Hz, 1H), 6.88 (s, 1H), 6.86 (s, 1H), 6.84 (dt, *J* = 3.4, 1.1 Hz, 1H), 6.00 (d, *J* =10.5 Hz, 1H), 5.45 (d, *J* = 10.5 Hz, 1H), 2.29 (s, 3H), 2.20 (s, 3H), 2.05 (s, 3H). 13C NMR (150 MHz, CDCl3) δ (ppm) 148.9, 144.4, 140.1, 139.5, 138.9, 136.7, 136.5, 136.4, 132.8, 129.0, 128.8, 128.3, 128.0, 127.3, 126.9, 126.8, 124.8, 124.2, 46.1, 21.1, 20.7, 20.5. HRMS (ASAP) calcd for $C_{28}H_{26}S$ $[M^*]^+$ 394.1755, obsd 394.1762.

2,5-bis(3-Mesityl-1,3-diphenylallyl)thiophene (5b-bis). Following the general procedure E, propargyl alcohol **1a** (107 mg, 0.514 mmol) was reacted with mesitylene (371 mg, 3.08 mmol) in the presence of $AuBr_3$ (11.2 mg, 0.026 mmol) for 15 min. Thiophene (21.6 mg, 0.257 mmol) was added and the reaction was complete after 24 h at room temperature. Purification by column chromatography (20:1 pentane:DCM) gave product **5b-bis** as a white, waxy solid in 37% yield (65.4 mg). ^1H NMR (600 MHz $(CD_3)_2SO$) d (ppm) 7.24-7.29 (m, 8H), 7.20-7.23 (m, 6 h), 7.17-7.19 (m, 2H), 7.11-7.13 (m, 4H), 6.93 (s, 2H), 6.92 (d, *J* = 10.4 Hz, 2H), 6.89 (s, 2H), 4.35 (d, *J* = 10.4 Hz, 2H), 2.28 (s, 3H), 2.27 (s, 3H), 1.89 (s, 6 h), 1.60 (s, 3H), 1.60 (s, 3H). ^{13}C NMR (150 MHz, $O{=}SC(CD_3)_2$ d (ppm) 146.2, 146.1, 143.1, 138.9, 138.6, 136.2, 135.7, 135.6, 134.3, 129.1, 128.5, 128.2, 128.2, 127.5, 126.6, 125.6, 123.8, 123.8, 46.4, 20.7, 19.4, 19.4, 19.2. HRMS (ASAP) $C_{52}H_{48}S$ [M*]+ 704.3477, obsd 704.3474.

2,5-bis(3-Mesityl-1,3-diphenylallyl)furan (5c-bis). The reaction was performed in pressure tube to prevent evaporation of furan. Following the general procedure D, propargyl alcohol **1a** (55.0 mg, 0.264 mmol) was reacted with mesitylene (190 mg, 1.58 mmol) in the presence of $AuBr_3$ (5.8 mg, 0.013 mmol) for 15 min. Furan (18.0 mg, 0.264 mmol) was added and the reaction was complete after 1.5 h at 80 °C. Purification by column chromatography (250:1 pentane:Et_2O) gave product **5c-bis** as an orange oil in 20% yield (18.0 mg). ^1H NMR (600 MHz, $CDCl_3$) d (ppm) 7.05-7.24 (m, 20 H), 7.08-7.10 (m, 2H), 7.05-7.07 (m, 2H), 6.66 (dd, *J* = 12.6, 10.2 Hz, 2H), 5.83 (d, *J* = 15.2 Hz, 2 H) 4.38 (d, *J* = 10.3 Hz, 2H), 2.33 (s, 3H), 2.34 (s, 3H), 1.94 (s, 3H), 1.96 (s, 3H), 1.77 (s, 6 h). ^{13}C NMR (150 MHz, $CDCl_3$) d (ppm) 155.8, 155.8, 141.9, 141.8, 140.0, 140.0, 139.8, 139.8, 136.9, 136.8, 136.7, 136.7, 136.6, 128.5, 128.5, 128.4, 128.4, 128.4, 128.4, 128.4, 128.1, 128.1, 128.1, 127.3, 127.3, 126.6, 126.6, 126.2, 106.6, 106.5, 45.5, 45.5, 21.3, 21.3, 19.9, 19.9, 19.9. HRMS (ASAP) calcd for $C_{52}H_{48}O$ [M*]+ 688.3705, obsd 688.3705.

3-(3-Mesityl-1,3-diphenylallyl)benzofuran (**5d**). Following the general procedure E, propargyl alcohol **1a** (68.7 mg, 0.330 mmol) was reacted with mesitylene (238 mg, 1.98 mmol) in the presence of $AuBr_3$ (7.2 mg, 0.017 mmol) for 15 min. Benzofuran (39.0 mg, 0.330 mmol) was added and the reaction was complete after 24 h at room temperature. Purification by column chromatography (200:1 pentane:DCM) gave product **5d** as a white solid in 53% yield (77.4 mg). ^1H NMR (600 MHz, $CDCl_3$) d (ppm) 7.47 (dd, *J* = 7.5, 1.1 Hz, 1H), 7.35-7.37 (m, 1H), 7.24-7.30 (m, 6 h), 7.14-7.22 (m, 6 h), 6.95 (s, 1H), 6.91 (s, 1H), 6.83 (d, *J* = 10.2 Hz, 1H), 6.44 (s, 1H), 4.62 (d, *J* = 10.1 Hz, 1H), 2.35 (s, 3H), 2.08 (s, 3H), 1.80 (s, 3H). ^{13}C NMR (150 MHz, $CDCl_3$) d (ppm) 159.9, 155.1, 141.1, 140.9, 137.0, 136.8, 136.7, 134.9, 128.7, 128.7, 128.6, 128.5, 128.2, 127.6, 127.0, 126.8, 126.2, 123.6, 122.6, 120.7, 111.2, 103.0, 45.9, 21.3, 20.1, 20.0. HRMS (ASAP) calcd for $C_{32}H_{28}O$ $[M^*]^+$ 428.2140, obsd 428.2144.

2-(3-Mesityl-1,3-diphenylallyl)benzo[b]thiophene **(5e^2)** ***and 3-(3-Mesityl-1,3-diphenylallyl)benzo[b]thiophene*** **(5e^3).** Following the general procedure E, propargyl alcohol **1a** (75.2 mg, 0.361 mmol) was reacted with mesitylene (260 mg, 2.17 mmol) in the presence of $AuBr_3$ (7.9 mg, 0.018 mmol) for 15 min. Benzothiophene (48.5 mg, 0.361 mmol) was added and the reaction was complete after 24 h at room temperature. Purification by column chromatography (40:1 pentane:toluene) gave product **5e** as a colourless oil in 32% yield (50.8 mg) as mixtures of 2- and 3-regioisomers **5e^2** and **5e^3**.

C2-regioisomer **5e^2** (analyzed from a 3:2 mix of 2-/3-isomers): ^1H NMR (600 MHz, $CDCl_3$) d (ppm) 7.68 (dd, *J* = 7.9, 0.9 Hz, 1H), 7.60 (dt, *J* = 7.9, 0.9 Hz, 1H), 7.08-7.28 (m, 13H), 6.92 (dd, *J* = 0.8 Hz, 1H), 6.91 (s, 1H), 6.87 (s, 1H), 6.84 (d, *J* = 10.3 Hz, 1H), 4.67 (d, *J* = 10.3 Hz, 1H), 2.32 (s, 3H), 2.04 (s, 3H), 1.73 (s, 3H). ^{13}C NMR (150 MHz, $CDCl_3$) d (ppm) 149.7, 143.1, 140.5, 139.9, 139.8, 139.8, 136.9, 136.8, 136.7, 134.8, 129.1, 128.7, 128.6, 128.5, 128.2, 127.6, 127.0, 126.3, 124.2, 123.8, 123.3, 122.3, 121.0, 47.4, 21.3, 20.3, 19.9. HRMS (ASAP) calcd for $C_{32}H_{28}S$ $[M^*]^+$ 444.1912, obsd 444.1913.

C3-regioisomer **5e^3**: ^{1}H NMR (600 MHz, $CDCl_3$) d (ppm) 7.81 (d, *J* = 8.0 Hz, 1H), 7.12-7.30 (m, 14H), 6.95 (s, 1H), 6.92 (d, *J* = 10.3 Hz, 1H), 6.84 (s, 1H), 4.80 (d, *J* = 10.3 Hz, 1H), 2.34 (s, 3H), 1.99 (s, 3H), 1.79 (s, 3H). ^{13}C NMR (150 MHz, $CDCl_3$) d (ppm) 143.1, 140.8, 140.1, 139.8, 138.3, 138.1, 137.0, 136.8, 136.5, 135.1, 129.4, 128.8, 128.6, 128.5, 127.9, 127.4, 126.5, 126.2, 124.2, 123.8, 123.0, 122.8, 122.6, 45.8, 21.3, 20.3, 20.0. HRMS (ASAP) calcd for $C_{32}H_{28}S$ [M*]$^+$ 444.1912, obsd 444.1911.

3-(3-Mesityl-1,3-diphenylallyl)benzofuran **(5d)**. Following the general procedure E, propargyl alcohol **1a** (68.7 mg, 0.330 mmol) was reacted with mesitylene (238 mg, 1.98 mmol) in the presence of $AuBr_3$ (7.2 mg, 0.017 mmol) for 15 min. Benzofuran (39.0 mg, 0.330 mmol) was added and the reaction was complete after 24 h at room temperature. Purification by column chromatography (200:1 pentane:DCM) gave product **5d** as a white solid in 53% yield (77.4 mg). ^{1}H NMR (600 MHz, $CDCl_3$) d (ppm) 7.47 (dd, *J* = 7.5, 1.1 Hz, 1H), 7.35-7.37 (m, 1H), 7.24-7.30 (m, 6 h), 7.14-7.22 (m, 6 h), 6.95 (s, 1H), 6.91 (s, 1H), 6.83 (d, *J* = 10.2 Hz, 1H), 6.44 (s, 1H), 4.62 (d, *J* = 10.1 Hz, 1H), 2.35 (s, 3H), 2.08 (s, 3H), 1.80 (s, 3H). ^{13}C NMR (150 MHz, $CDCl_3$) d (ppm) 159.9, 155.1, 141.1, 140.9, 137.0, 136.8, 136.7, 134.9, 128.7, 128.7, 128.6, 128.5, 128.2, 127.6, 127.0, 126.8, 126.2, 123.6, 122.6, 120.7, 111.2, 103.0, 45.9, 21.3, 20.1, 20.0. HRMS (ASAP) calcd for $C_{32}H_{28}O$ [M*]$^+$ 428.2140, obsd 428.2144.

2-(3-Mesityl-1,3-diphenylallyl)benzo[b]thiophene **(5e^2)** ***and 3-(3-mesityl-1,3-diphenylallyl)benzo[b]thiophene*** **(5e^3).** Following the general procedure E, propargyl alcohol **1a** (75.2 mg, 0.361 mmol) was reacted with mesitylene (260 mg, 2.17 mmol) in the presence of $AuBr_3$ (7.9 mg, 0.018 mmol) for 15 min. Benzothiophene (48.5 mg, 0.361 mmol) was added and the reaction was complete after 24 h at room temperature. Purification by column chromatography (40:1 pentane:toluene) gave product **5e** as a colorless oil in 32% yield (50.8 mg) as mixtures of 2- and 3-regioisomers **5e^2** and **5e^3**.

C2-regioisomer **5e^2** (analyzed from a 3:2 mix of 2-/3-isomers): ^{1}H NMR (600 MHz, $CDCl_3$) d (ppm) 7.68 (dd, *J* = 7.9, 0.9 Hz, 1H), 7.60 (dt, *J* = 7.9, 0.9 Hz, 1H), 7.08-7.28 (m, 13H), 6.92 (dd, *J* = 0.8 Hz, 1H), 6.91 (s, 1H), 6.87 (s, 1H), 6.84 (d, *J* = 10.3 Hz, 1H), 4.67 (d, *J* = 10.3 Hz, 1H), 2.32 (s, 3H), 2.04 (s, 3H), 1.73 (s, 3H). ^{13}C NMR (150 MHz, $CDCl_3$) d (ppm) 149.7, 143.1, 140.5, 139.9, 139.8, 139.8, 136.9, 136.8, 136.7, 134.8, 129.1, 128.7, 128.6, 128.5, 128.2, 127.6, 127.0, 126.3, 124.2, 123.8, 123.3, 122.3, 121.0, 47.4, 21.3, 20.3, 19.9. HRMS (ASAP) calcd for $C_{32}H_{28}S$ [M*]$^+$ 444.1912, obsd 444.1913.

C3-regioisomer **5e^3**: ^{1}H NMR (600 MHz, $CDCl_3$) d (ppm) 7.81 (d, *J* = 8.0 Hz, 1H), 7.12-7.30 (m, 14H), 6.95 (s, 1H), 6.92 (d, *J* = 10.3 Hz, 1H), 6.84 (s, 1H), 4.80 (d, *J* = 10.3 Hz, 1H), 2.34 (s, 3H), 1.99 (s, 3H), 1.79 (s, 3H). ^{13}C NMR (150 MHz, $CDCl_3$) d (ppm) 143.1, 140.8, 140.1, 139.8, 138.3, 138.1, 137.0, 136.8, 136.5, 135.1, 129.4, 128.8, 128.6, 128.5, 127.9, 127.4, 126.5, 126.2, 124.2, 123.8, 123.0, 122.8, 122.6, 45.8, 21.3, 20.3, 20.0. HRMS (ASAP) calcd for $C_{32}H_{28}S$ [M*]$^+$ 444.1912, obsd 444.1911.

(1-Mesityl-3-(4-methoxyphenyl)prop-1-ene-1,3-diyl)dibenzene (5f). Following the general procedure E, propargyl alcohol **1a** (57.7 mg, 0.277 mmol) was reacted with mesitylene (200 mg, 1.66 mmol) in the presence of $AuBr_3$ (6.1 mg, 0.014 mmol) for 15 min. Anisole (30.0 mg, 0.277 mmol) was added and the reaction was complete after 48 h at room temperature. Purification by column chromatography (200:1 pentane:DCM) gave product **5f** as a colorless oil in 42% yield (46.0 mg). ^{1}H NMR (600 MHz, $CDCl_3$) d (ppm) 7.25-7.30 (m, 6 h), 7.22 (tt, *J* = 6.9, 1.7 Hz, 1H), 7.18 (tt, *J* = 7.3, 1.4 Hz, 1H), 7.12-7.14 (m, 2H), 7.04-7.06 (m, 2H), 6.92 (s, 2H), 6.85 (d, *J* = 10.3 Hz, 1H), 6.80-6.83 (m, 2H), 4.42 (d, *J* = 10.3 Hz, 1H), 3.79 (s, 3H), 2.36 (s, 3H), 1.90 (s, 3H), 1.89 (s, 3H). ^{13}C NMR (150 MHz, $CDCl_3$) d (ppm) 158.1, 144.8, 140.2, 139.3, 136.8, 136.7, 136.7, 135.3, 130.7, 129.3, 128.5, 128.5, 128.4, 128.4, 128.3, 127.2, 126.2, 126.1, 113.9, 55.3, 49.9, 21.3, 20.1, 20.1. HRMS (ASAP) calcd for $C_{31}H_{30}O$ [M*]$^+$ 418.2297, obsd 418.2304.

(1-Mesityl-3-(2,4,6-trimethoxyphenyl)prop-1-ene-1,3-diyl)dibenzene (5g). Following the general procedure E, propargyl alcohol **1a** (55.2 mg, 0.265 mmol) was reacted with mesitylene (191 mg, 1.59 mmol) in the presence of $AuBr_3$ (5.8 mg, 0.013 mmol) for 15 min. 1,3,5-Trimethoxybenzene (44.6 mg, 0.265 mmol) was added, and the reaction was complete after 24 h at room temperature. Purification by column chromatography (30:1 pentane:Et_2O) gave product **5g** as a 7:3 mixture of **5g** and 1,3,5-trimethoxybenzene as a white solid in 58% yield of **5g** (84.1 mg mixture, 73.5 mg). ^{1}H NMR (600 MHz, $CDCl_3$) d (ppm) 7.19-7.21 (m, 2H), 7.10-7.15 (m, 7H), 7.07 (tt, *J* = 6.9, 1.6 Hz, 1H), 6.99-7.03 (m, 1H), 6.80 (s, 1H), 6.67 (s, 1H), 5.95 (s, 2H), 4.97 (d, *J* = 9.4 Hz, 1H), 3.69 (s, 3H), 3.42 (s, 6 h), 2.21 (s, 3H), 2.06 (s, 3H), 1.42 (s, 3H). ^{13}C NMR (150 MHz, $CDCl_3$) d (ppm)) 159.9, 159.0, 145.2, 140.8, 138.8, 137.4, 136.2, 135.9, 135.9, 131.7, 128.4, 127.9, 127.9, 127.9, 127.3, 126.7, 126.0, 125.3, 112.7, 90.8, 55.6, 55.4, 39.4, 21.2, 20.2, 19.4. HRMS (ASAP) calcd for $C_{33}H_{35}O_3$ [M*]$^+$ 479.2586, obsd 479.2582.

References

[1] H.A. Stefani, R. Cella, F.A. Dörr, C.M.P. De Pereira, F.P. Gomes, G. Zeni, Tetrahedron Lett. 46 (2005) 2001–2003.

[2] E.E. Wilson, A.G. Oliver, R.P. Hughes, B.L. Ashfeld, Organometallics. 30 (2011) 5214–5221.

[3] K. Wadhwa, V.R. Chintareddy, J.G. Verkade, J. Org. Chem. 74 (2009) 6681–6690.

[4] X. Yao, C.-J. Li, Org. Lett. 7 (2005) 4395–4398.

[5] G. Aridoss, V.D. Sarca, J.F. Ponder, J. Crowe, K.K. Laali, Org. Biomol. Chem. 9 (2011) 2518–2529.

[6] G.M. Martins, D.F. Back, T.S. Kaufman, C.C. Silveira, J. Org. Chem. 83 (2018) 3252–3264.

[7] R. Sanz, A. Martínez, J.M. Álvarez-Gutiérrez, F. Rodríguez, Eur. J. Org. Chem. 2006 (2006) 1383–1386.

[8] S.S. Weng, K.Y. Hsieh, Z.J. Zeng, Tetrahedron. 71 (2015) 2549–2554.

[9] N. Morita, M. Miyamoto, A. Yoda, M. Yamamoto, S. Ban, Y. Hashimoto, O. Tamura, Tetrahedron Lett. 57 (2016) 4460–4463.

[10] D.W. Jones, A. Pomfret, J. Chem. Soc. Perkin Trans. 1. (1991) 249–253.

^{1}H NMR spectrum of **1a**

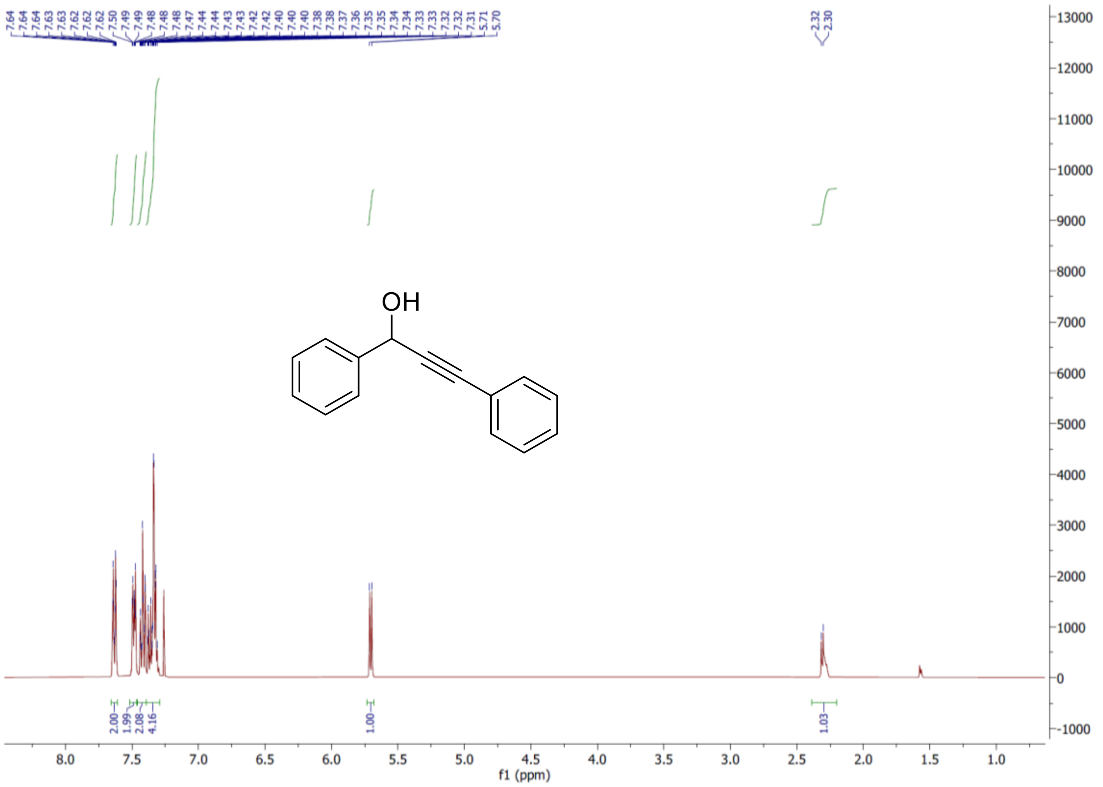

^{1}H NMR spectrum of **1b**

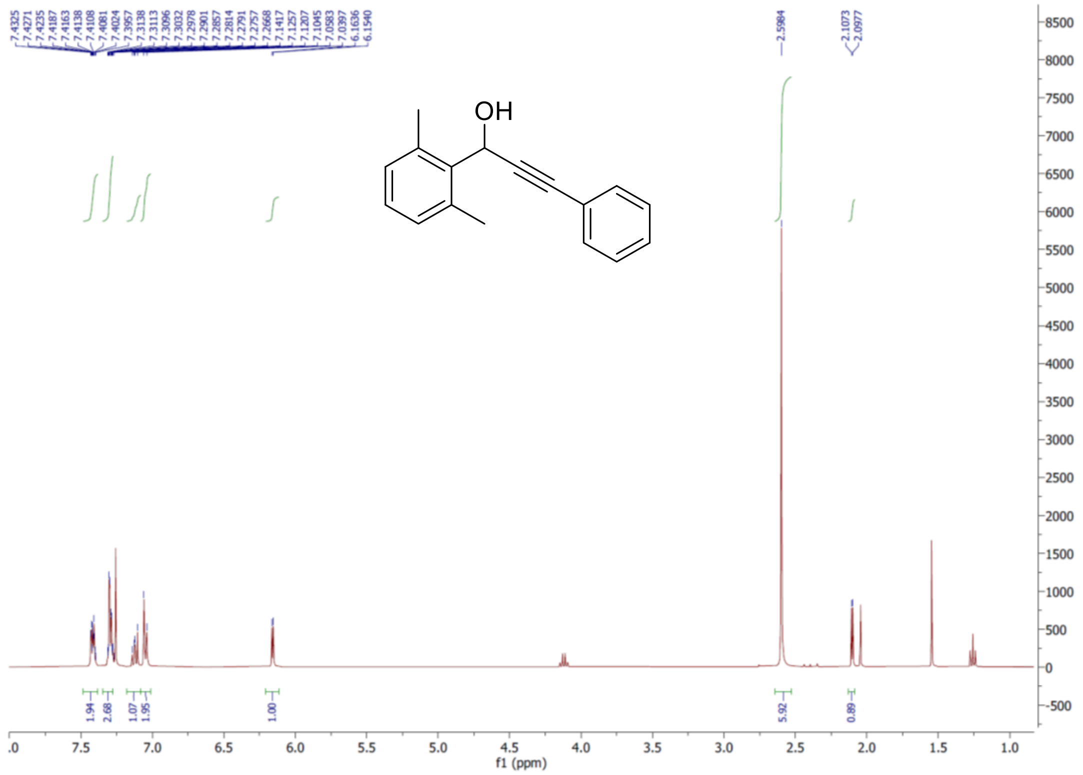

^{1}H NMR of **1c**

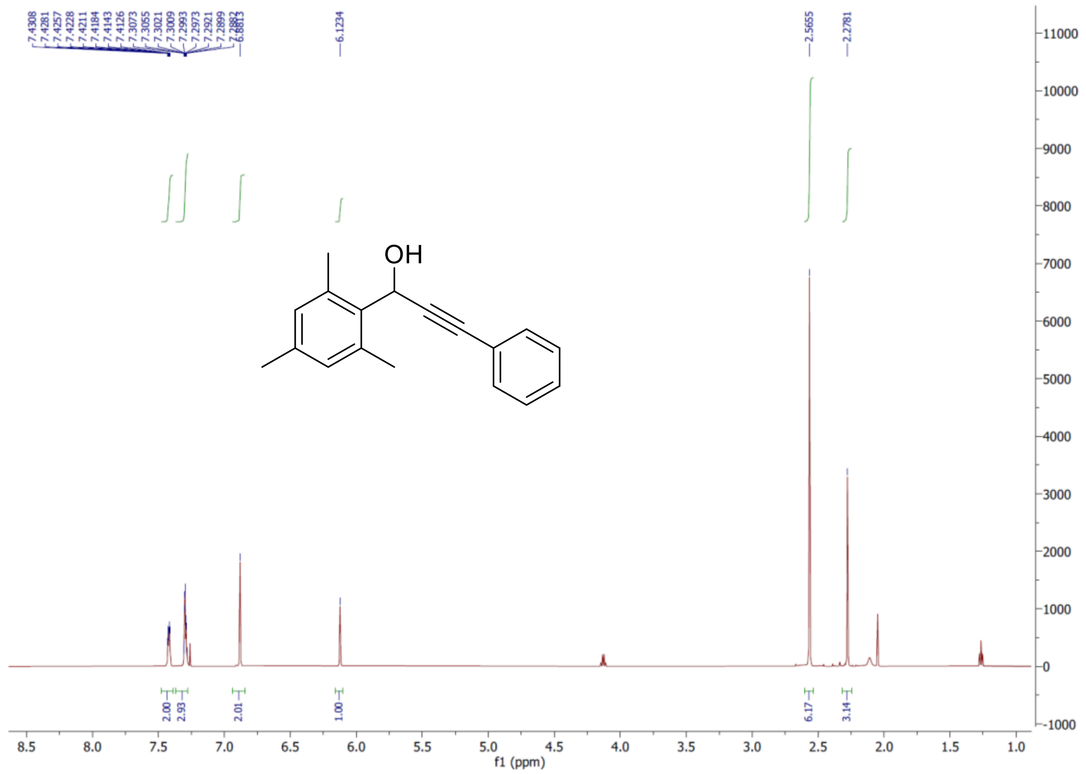

^{13}C NMR spectrum of **1c**

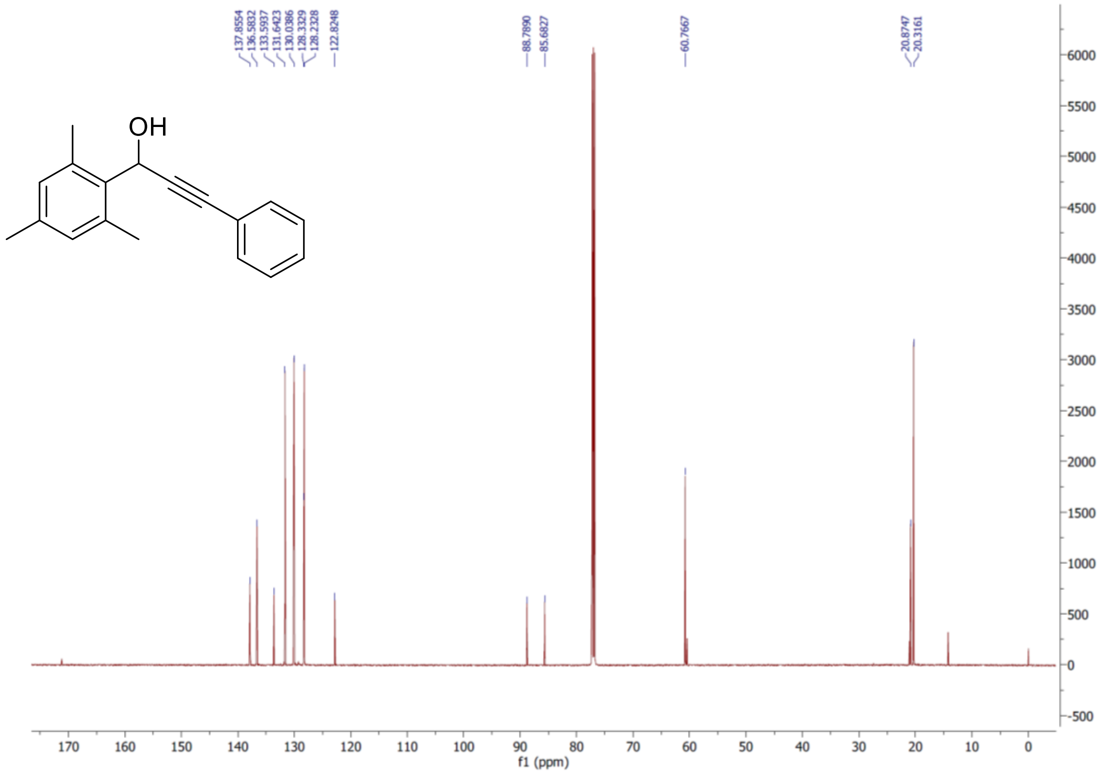

^{1}H NMR spectrum of **1d**

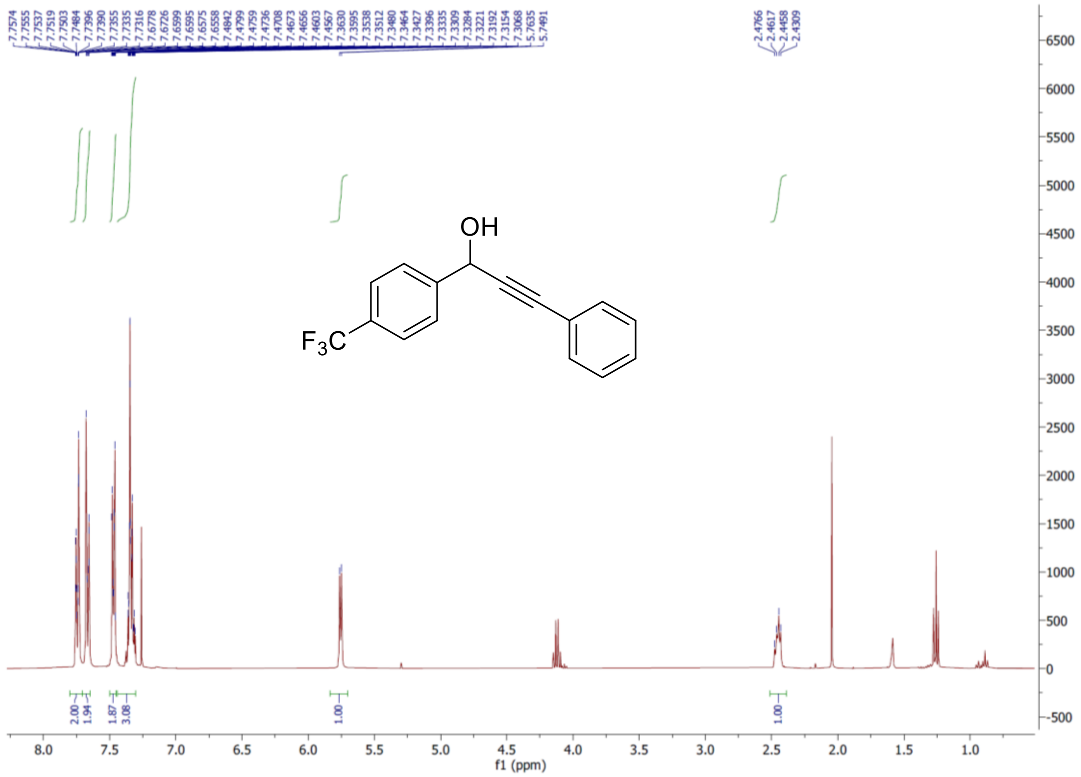

^{1}H NMR spectrum of **1e**

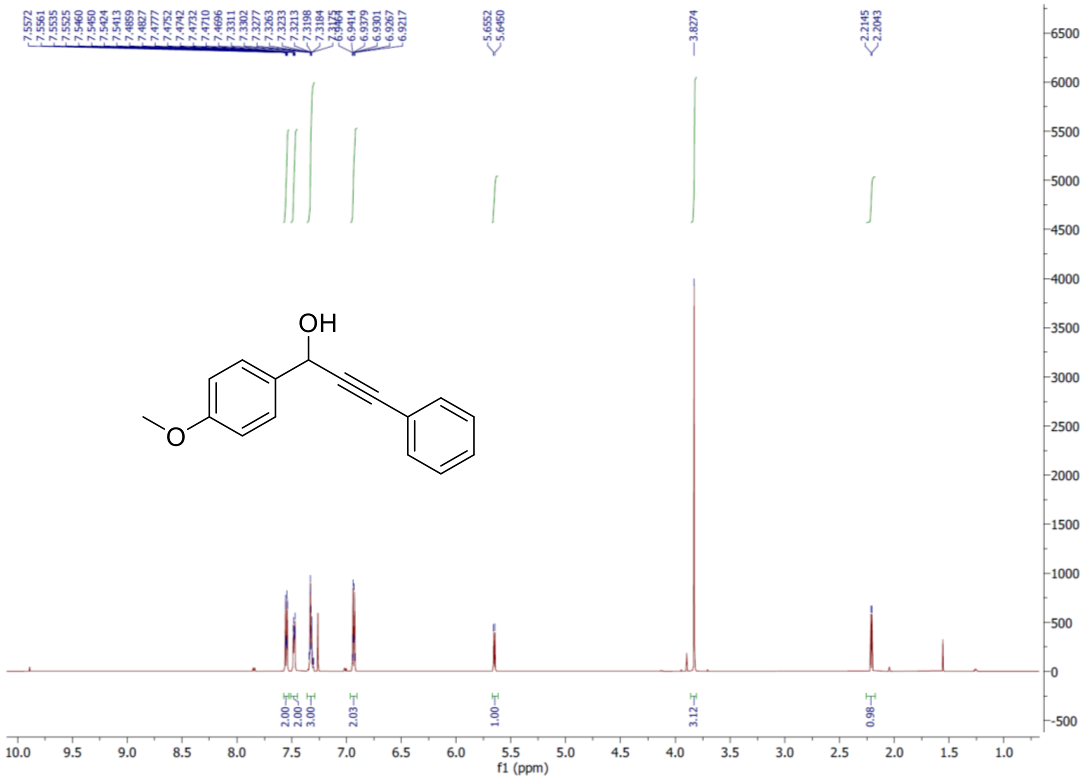

^{1}H NMR of **1f**

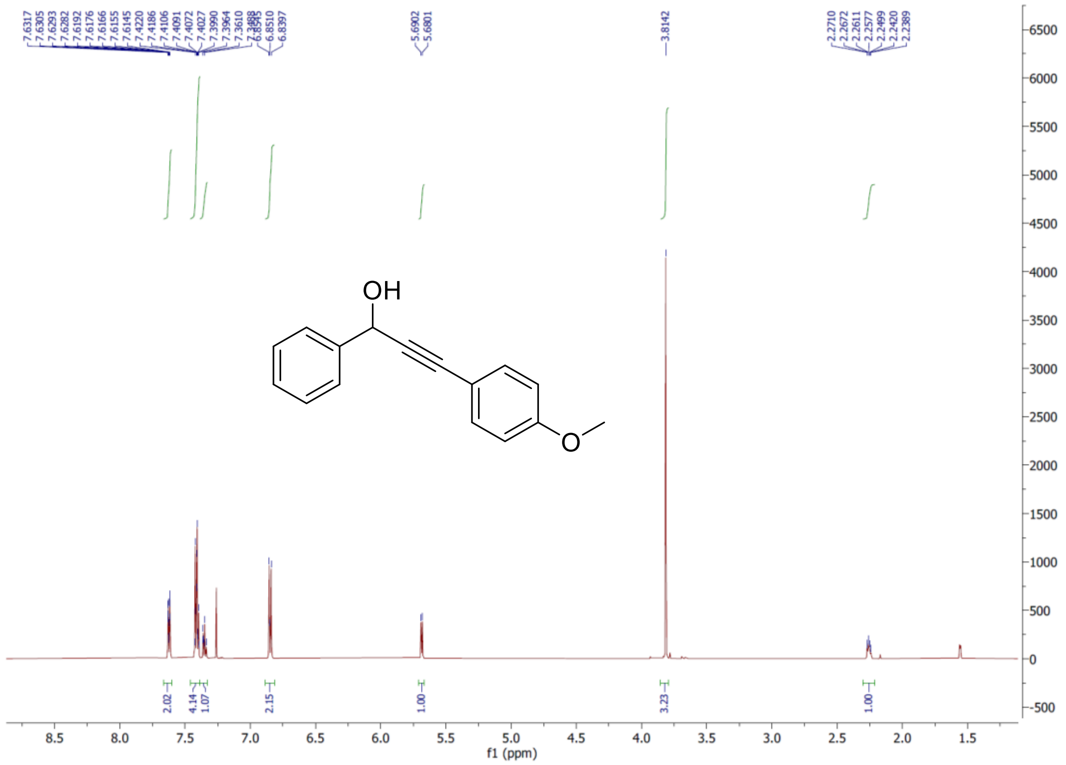

^{13}C NMR of **1f**

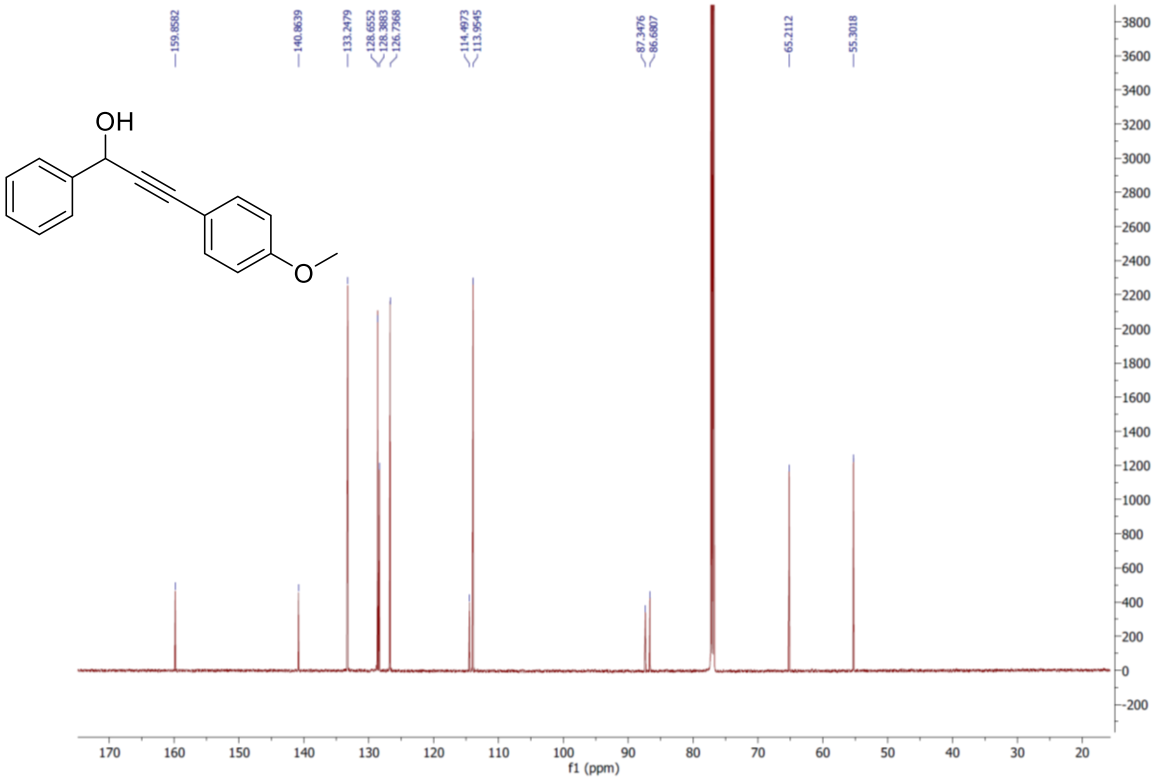

^{1}H NMR spectrum of **1g**

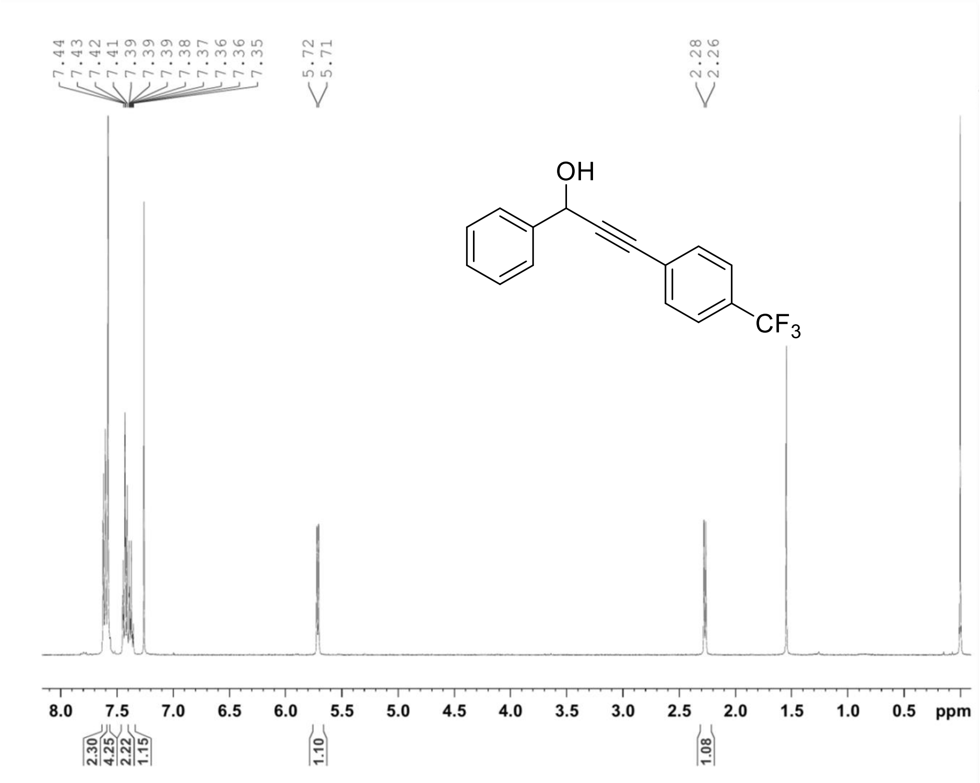

^{1}H NMR spectrum of **1h**

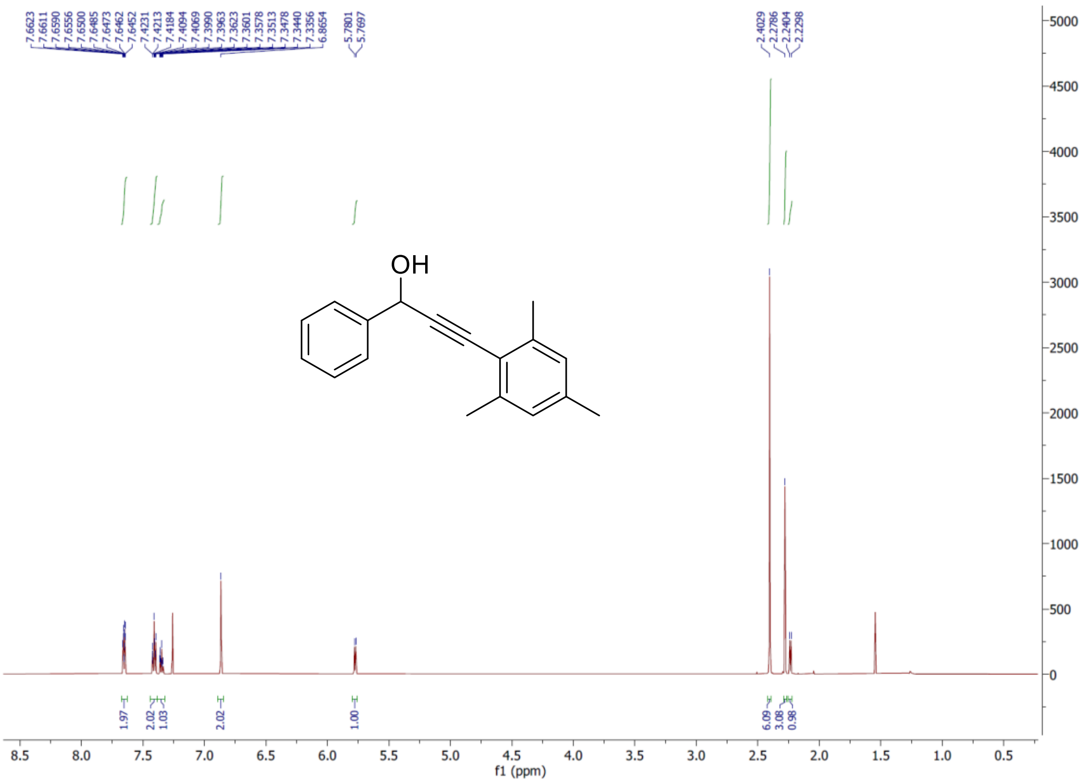

^{13}C spectrum NMR of **1h**

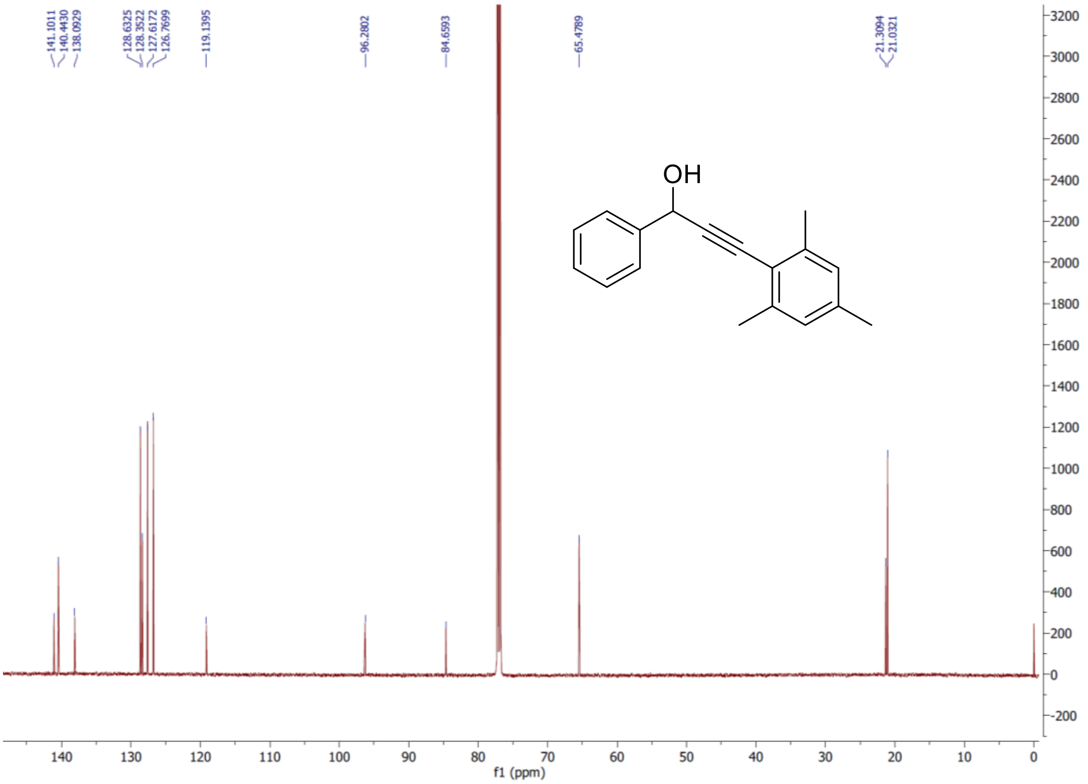

^{1}H NMR spectrum of **1i**

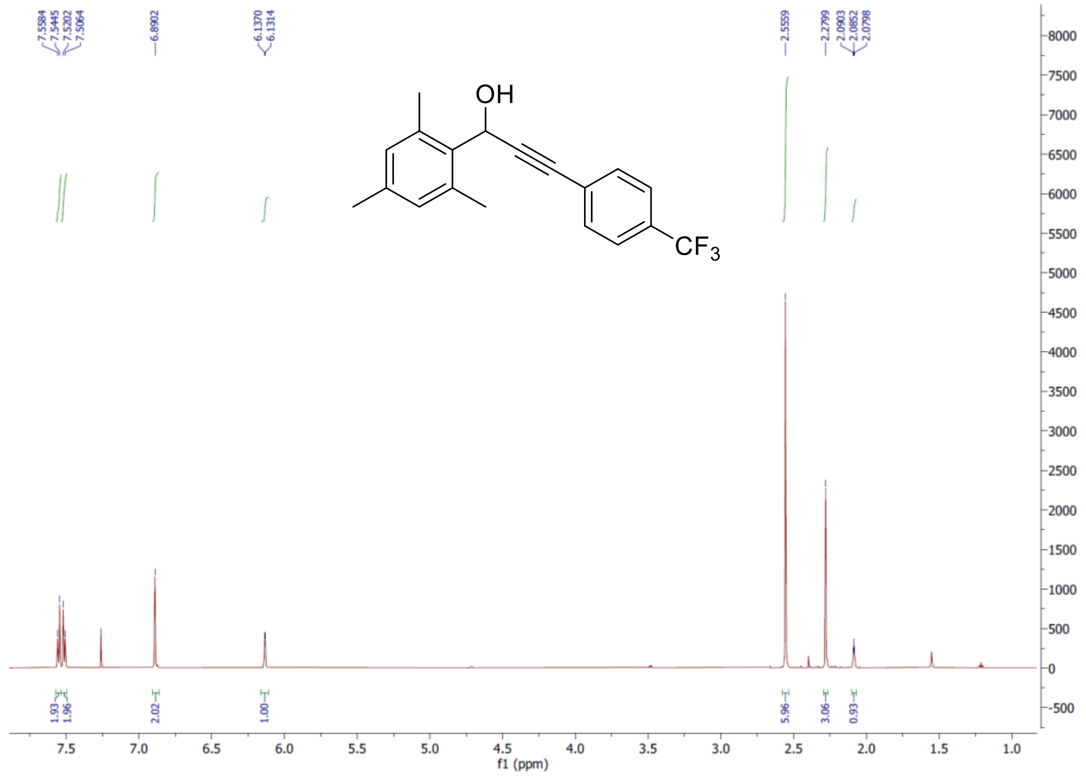

^{13}C NMR spectrum of **1i**

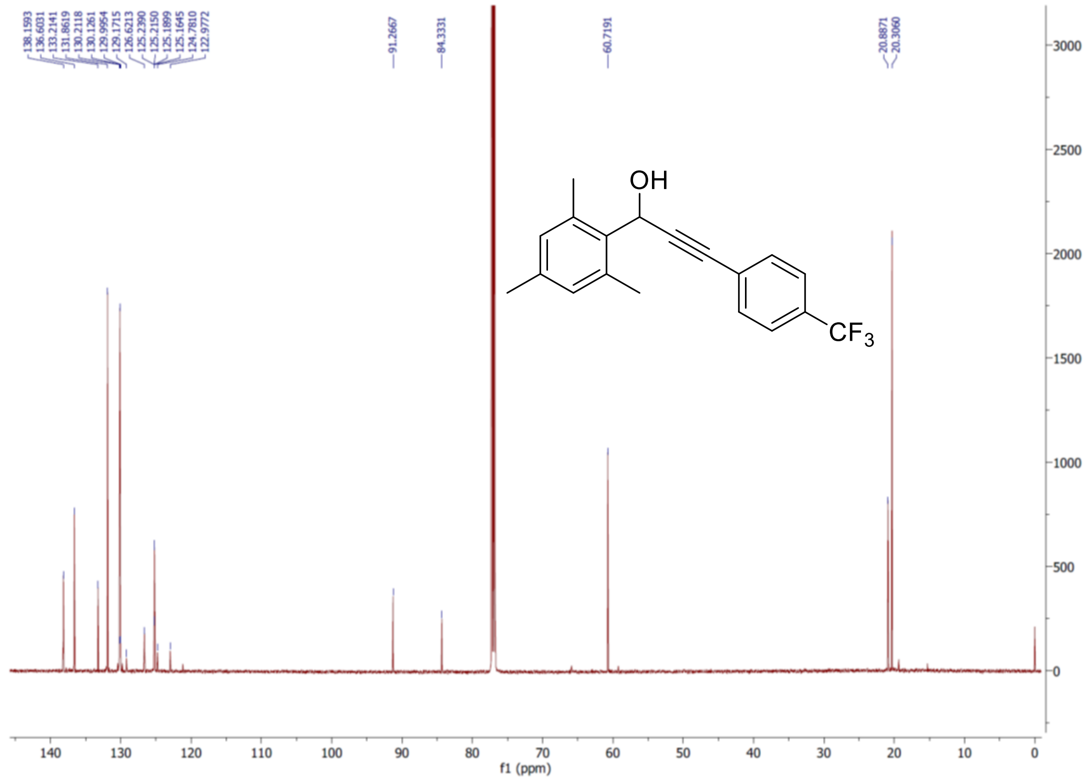

^{1}H NMR spectrum of **2a (**4:1mixture of **2a** + **4a**)

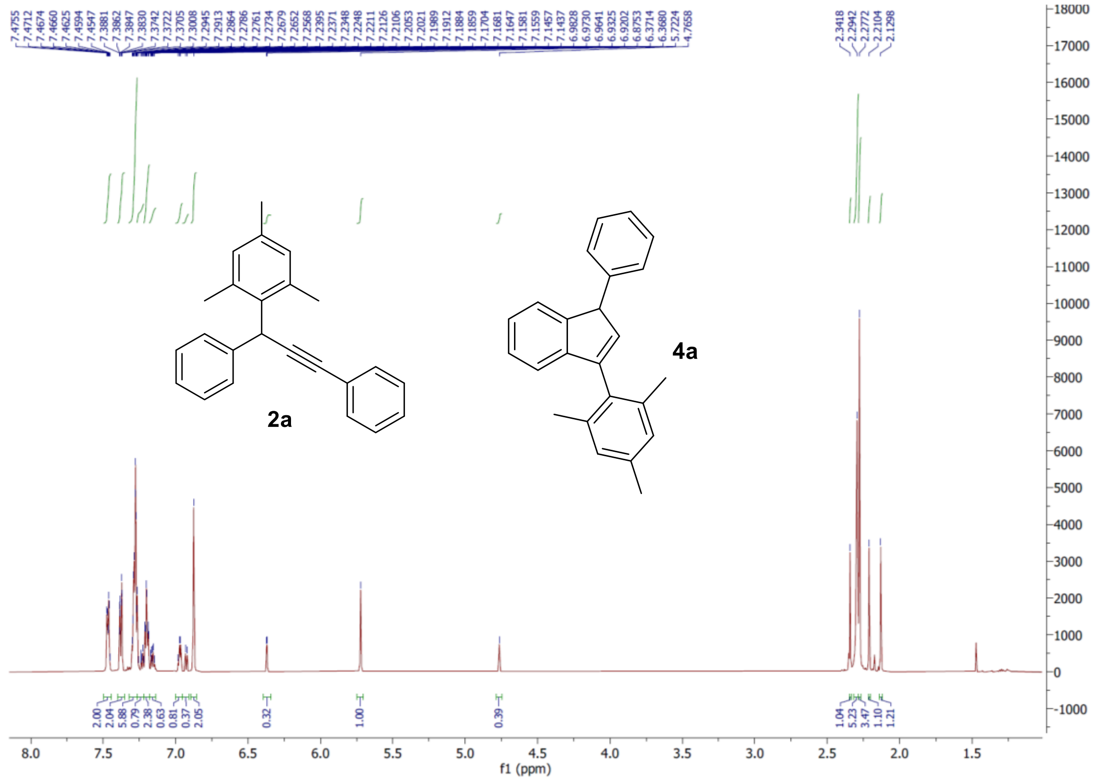

^{1}H NMR spectrum of **2d**

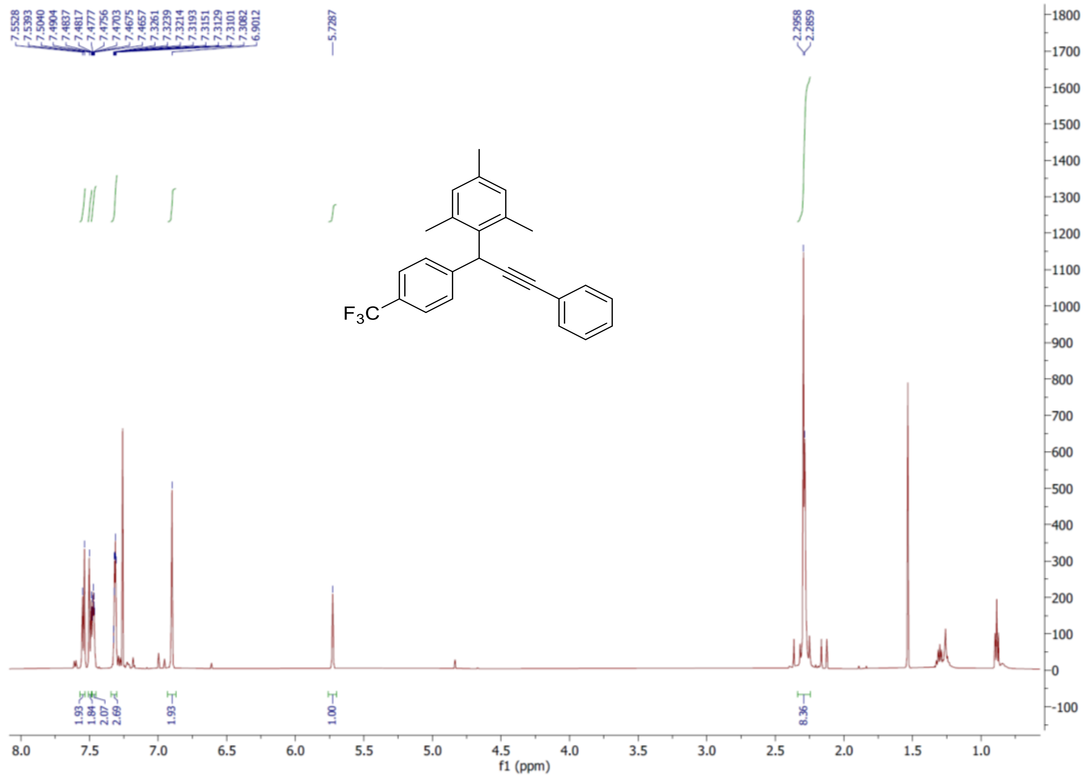

^{1}H NMR spectrum of **2g** (3:1 mixture of **2g** + **3g**)

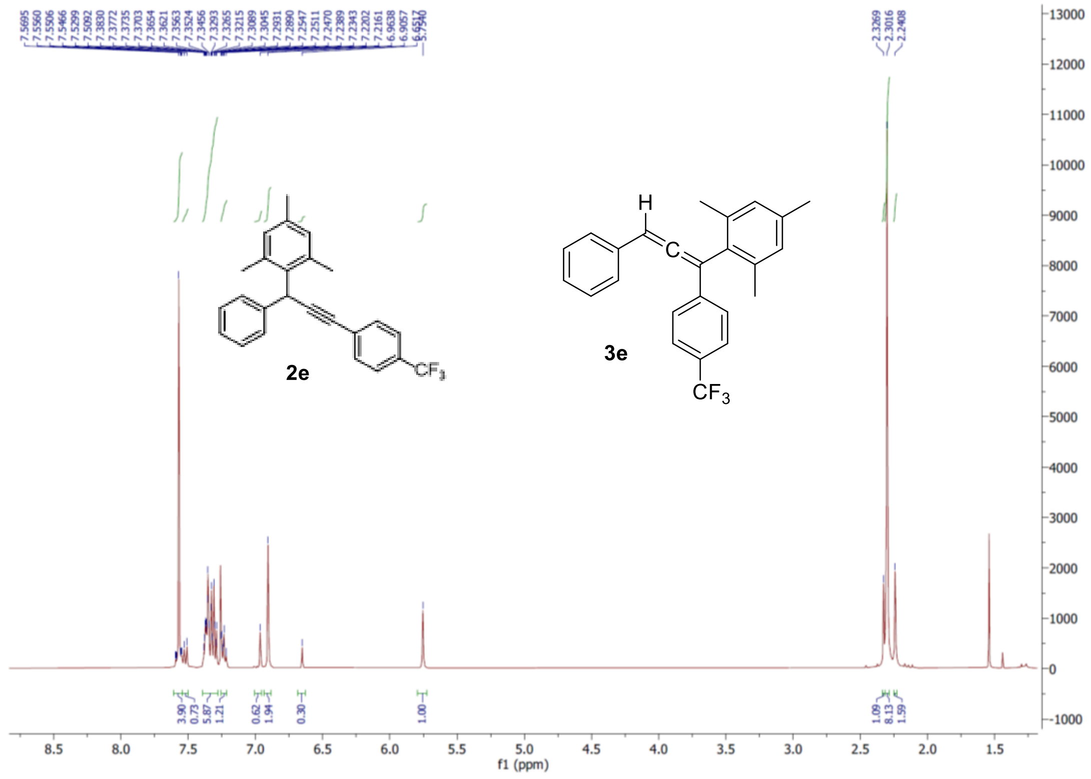

^{1}H NMR spectrum of **2l**

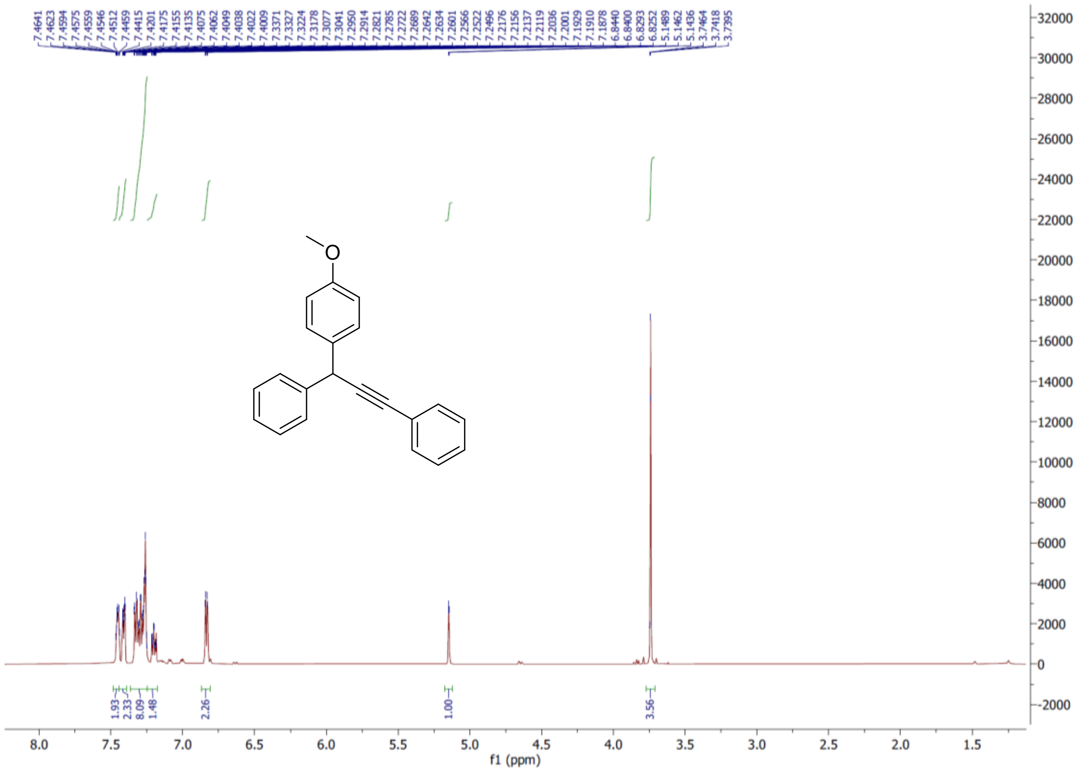

^{1}H NMR spectrum of **2m**

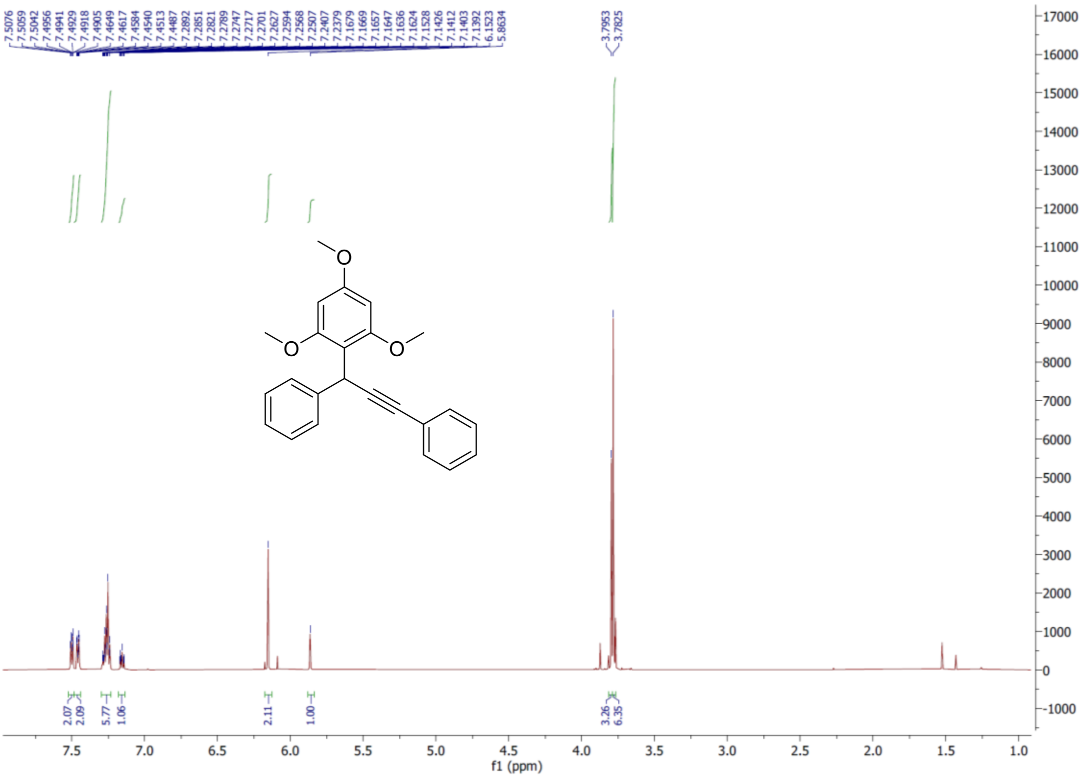

^{1}H NMR spectrum of **2a$^{\mathbf{OEt}}$**

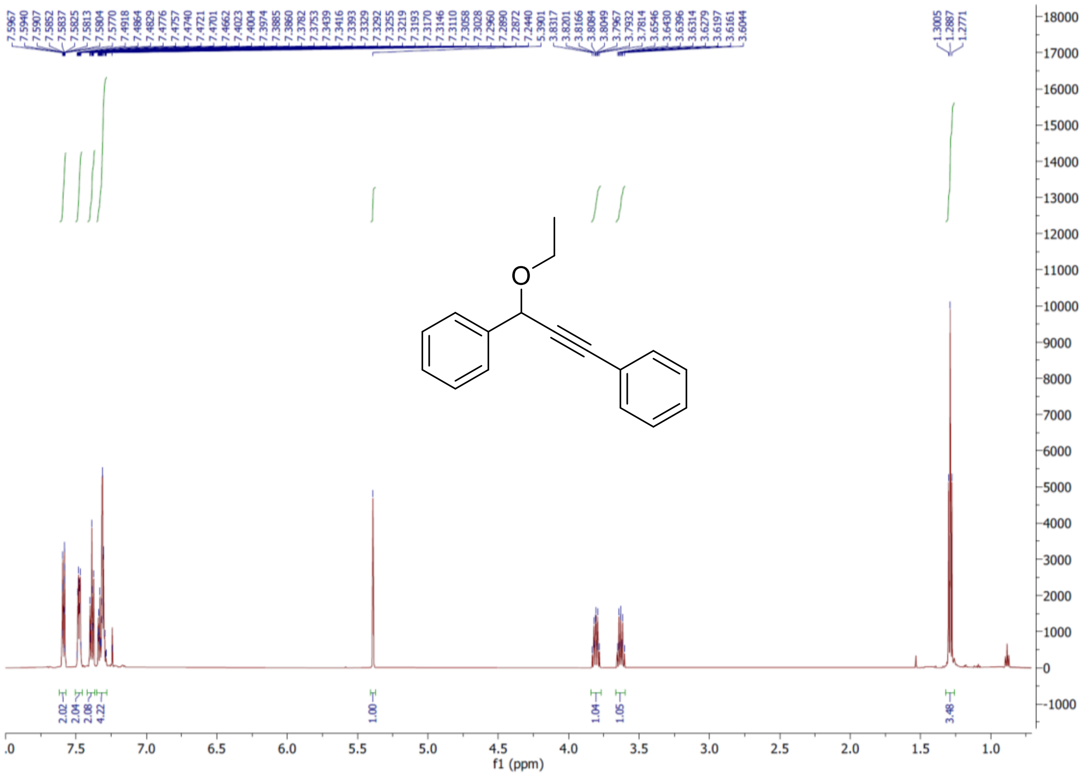

^{1}H NMR spectrum of **2a$^{CF3\text{-}EtO}$**

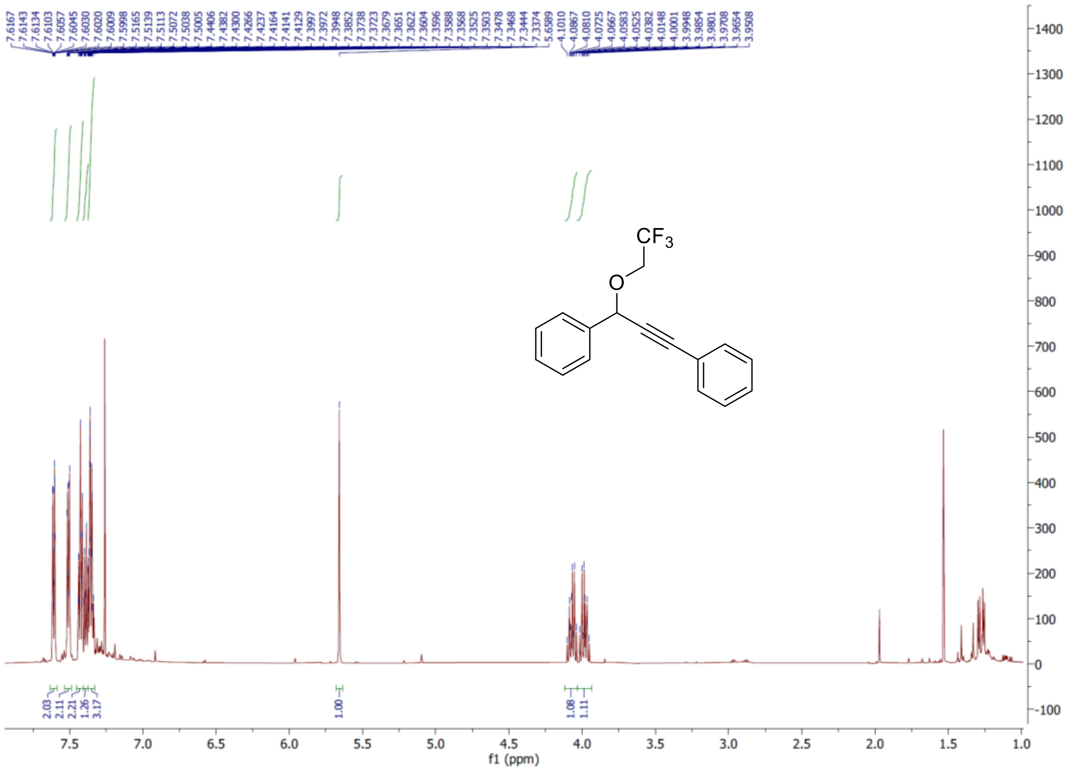

^{13}C NMR spectrum of **2a$^{CF3\text{-}EtO}$**

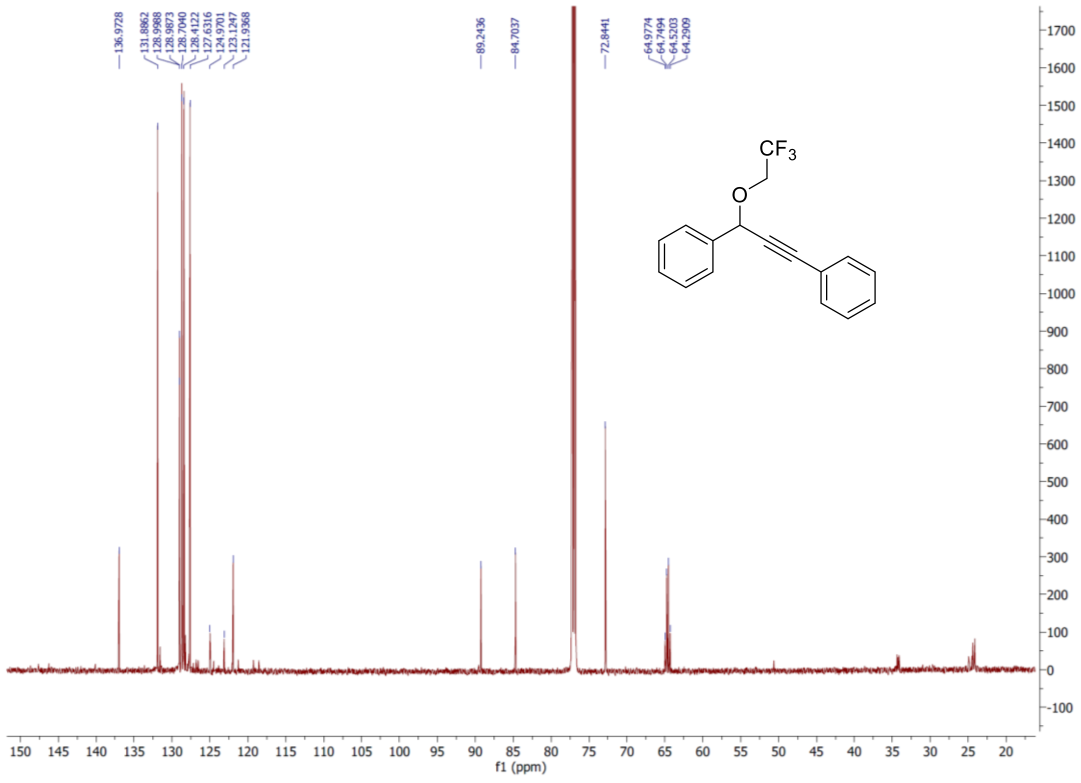

^{1}H NMR spectrum of **2e$^{CF3\text{-}EtO}$**

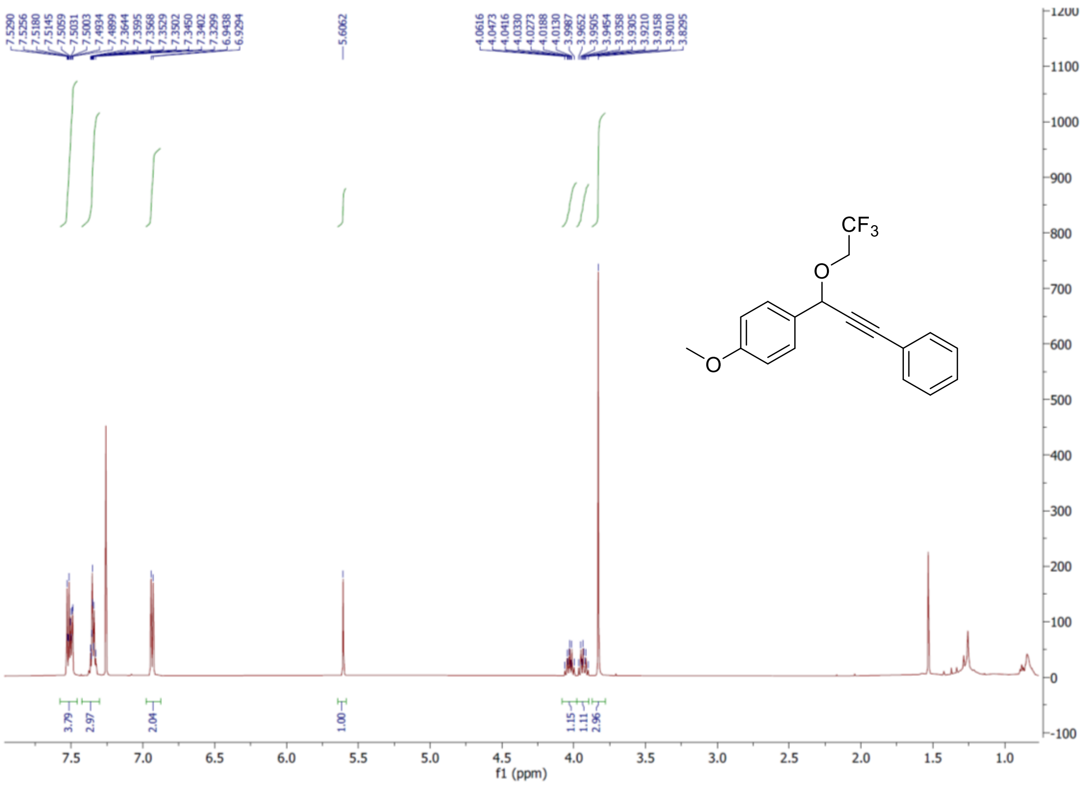

^{13}C NMR spectrum of **2e$^{CF3\text{-}EtO}$**

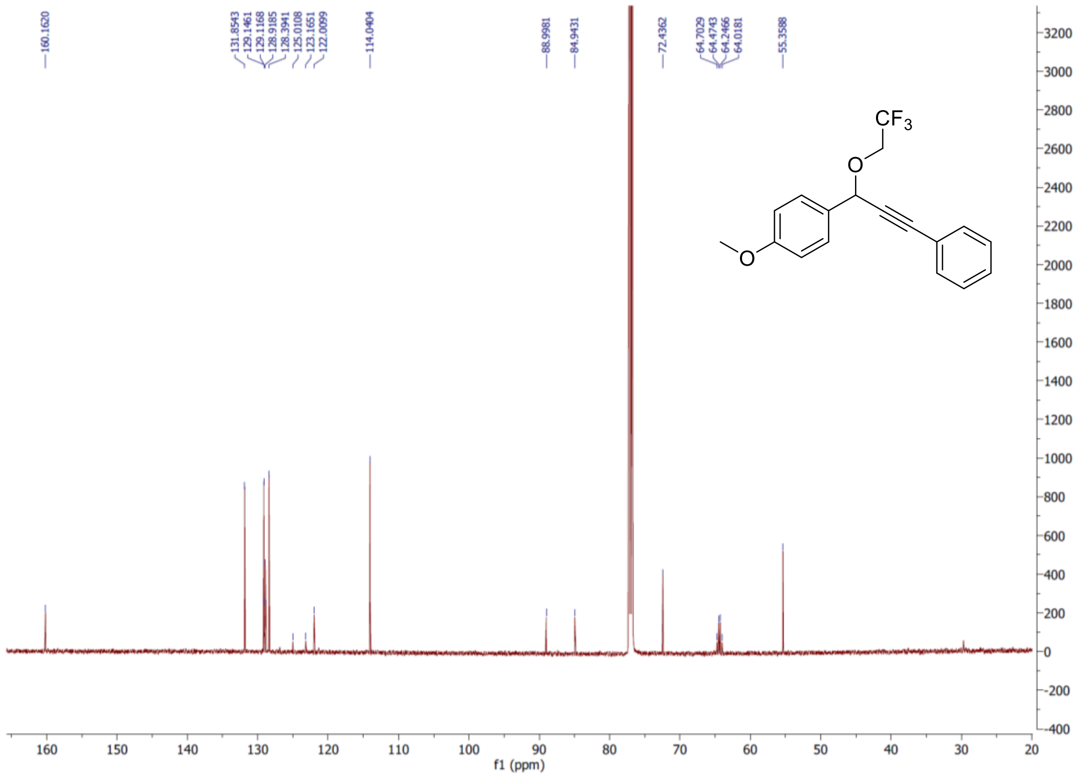

^{1}H NMR spectrum of **3a**

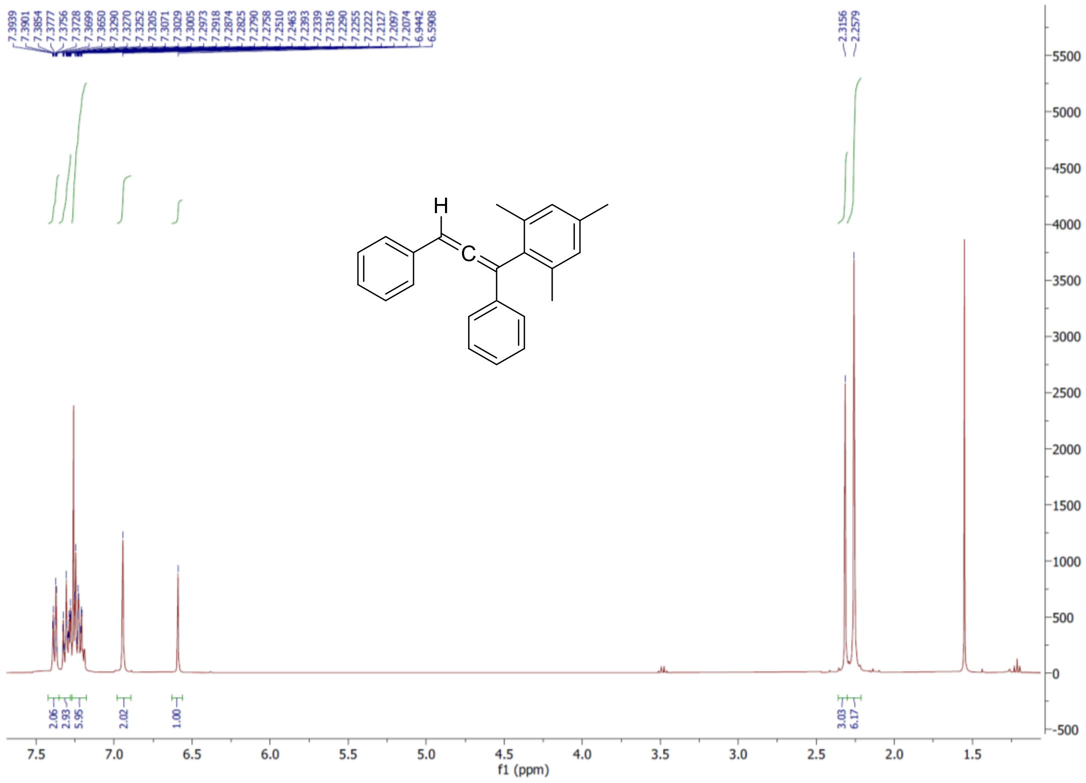

^{1}H NMR spectrum of **3b**

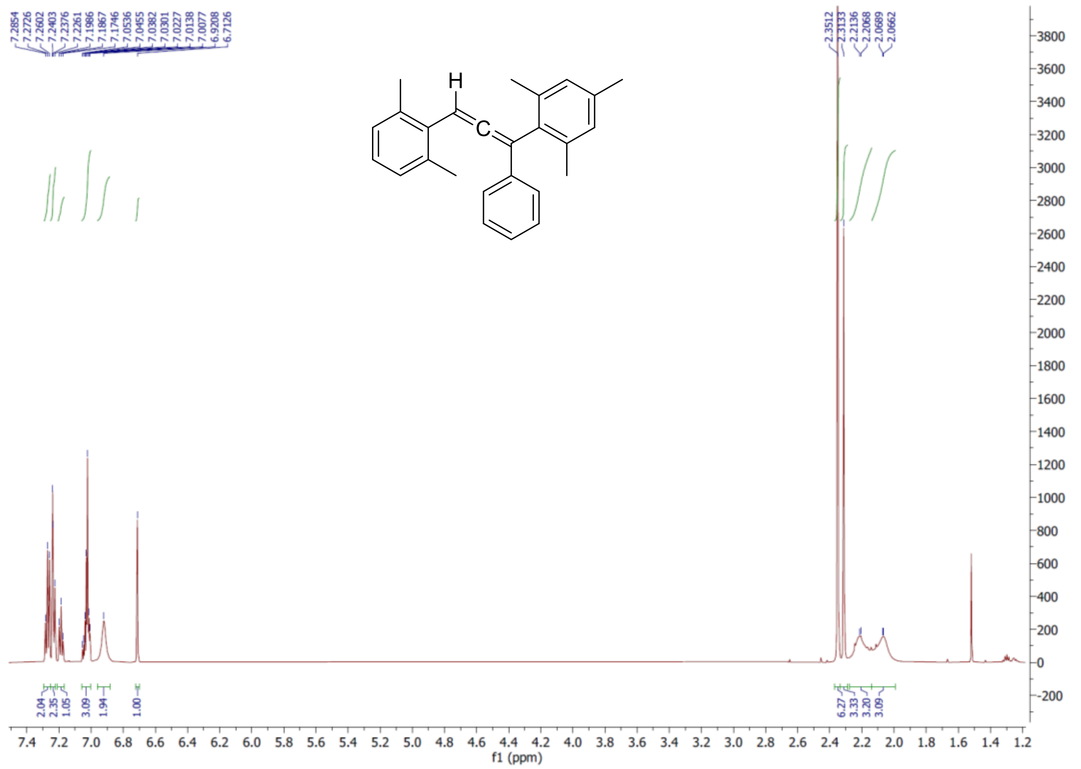

^{1}H NMR spectrum of **3c**

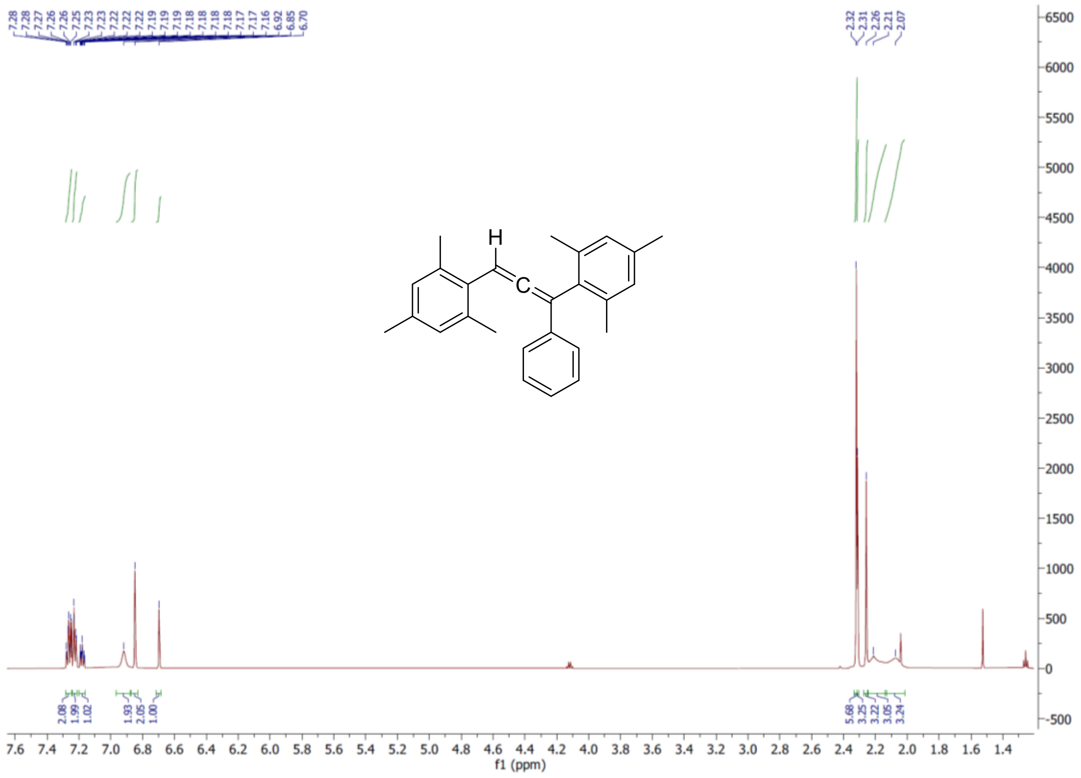

^{13}C NMR spectrum of **3c**

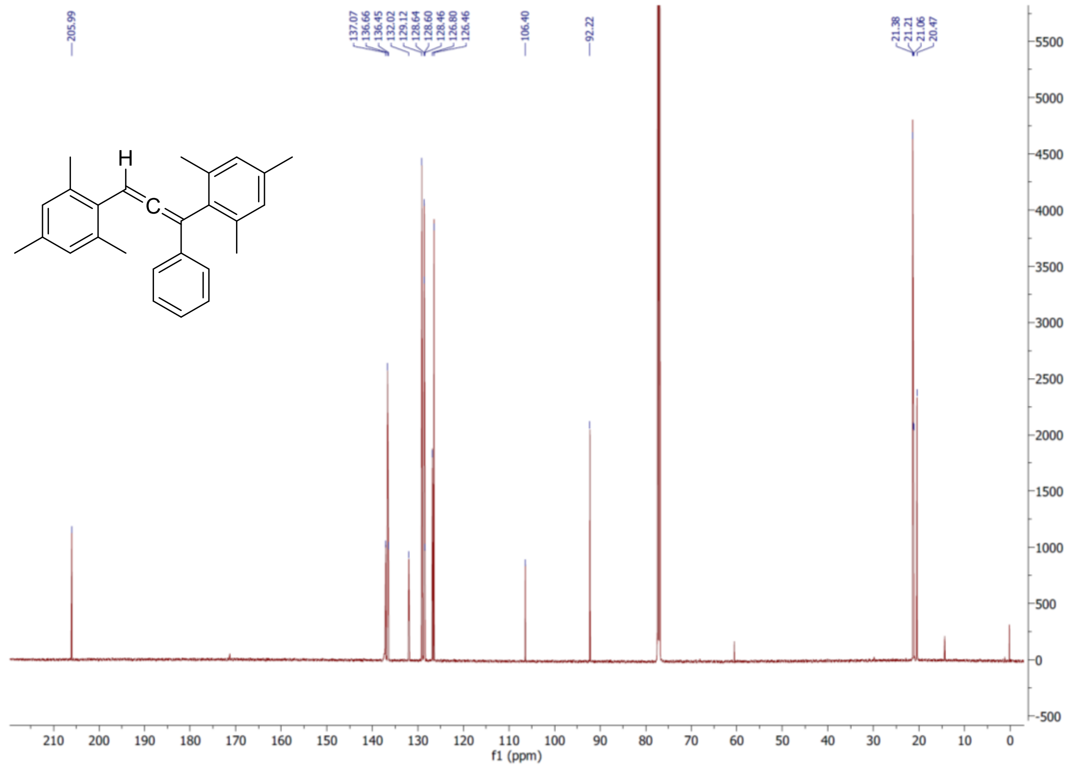

^{1}H NMR spectrum of **3d**

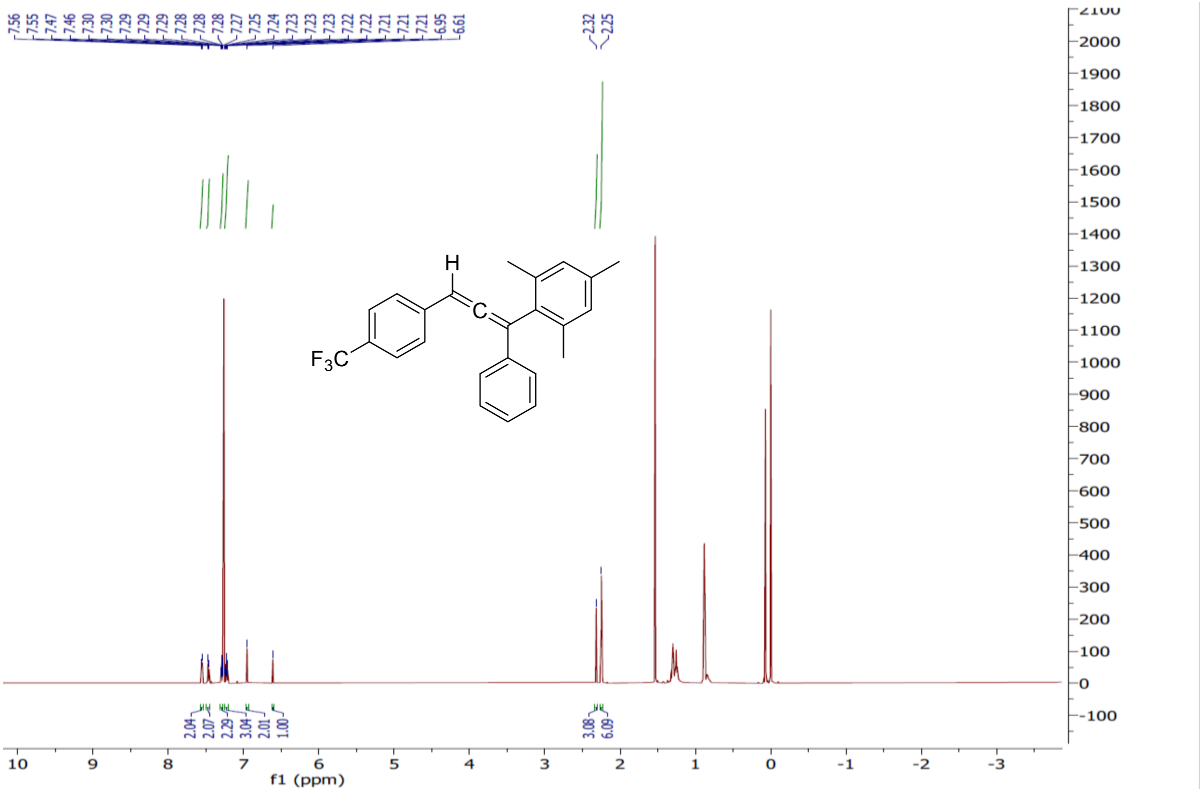

^{13}C NMR spectrum of **3d**

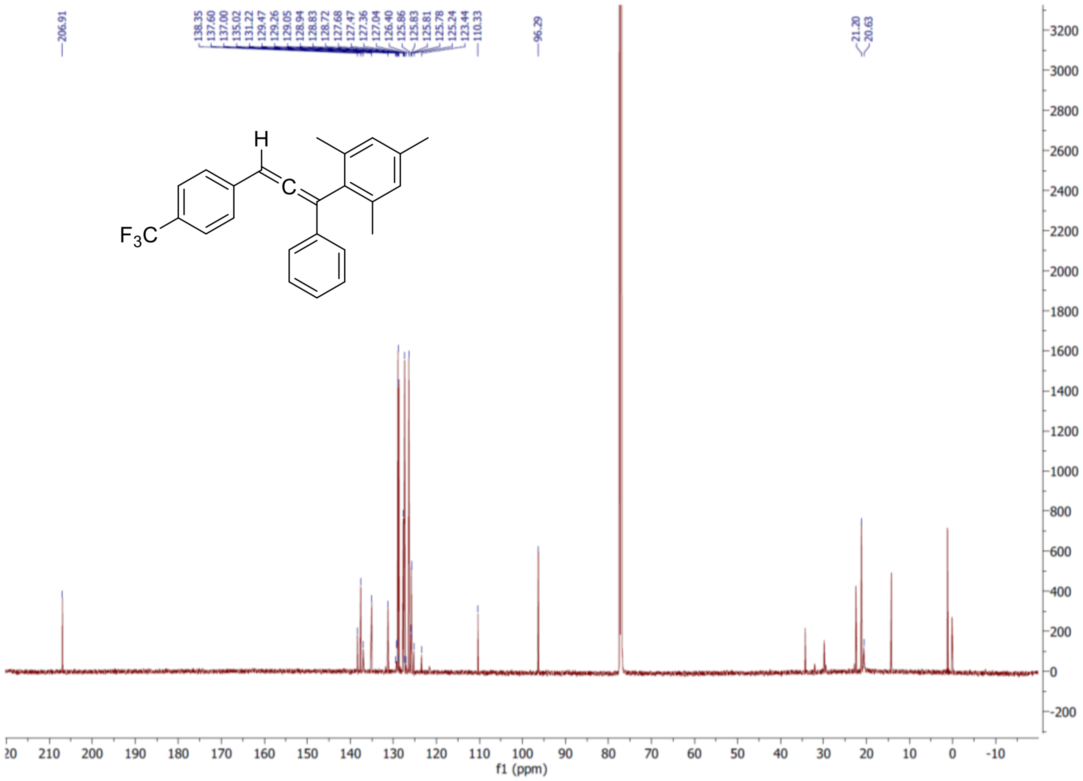

^{1}H NMR spectrum of **3g (**4:1mixture of **3g** + **2g)**

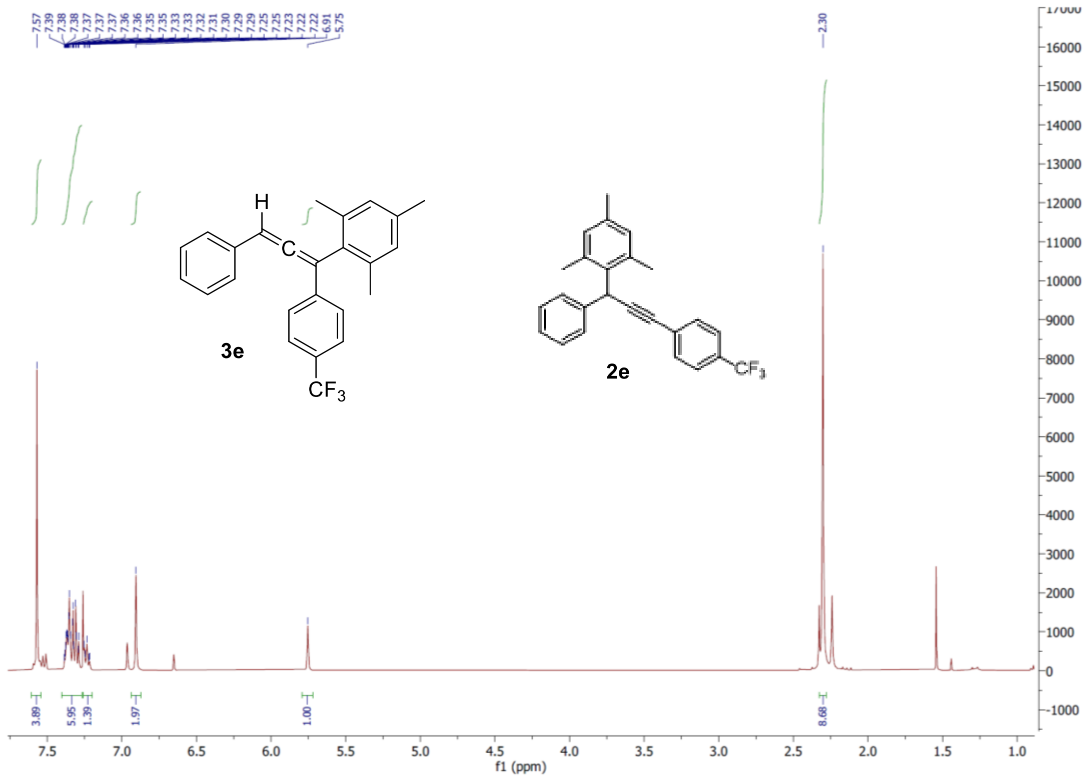

^{1}H NMR spectrum of **3i**

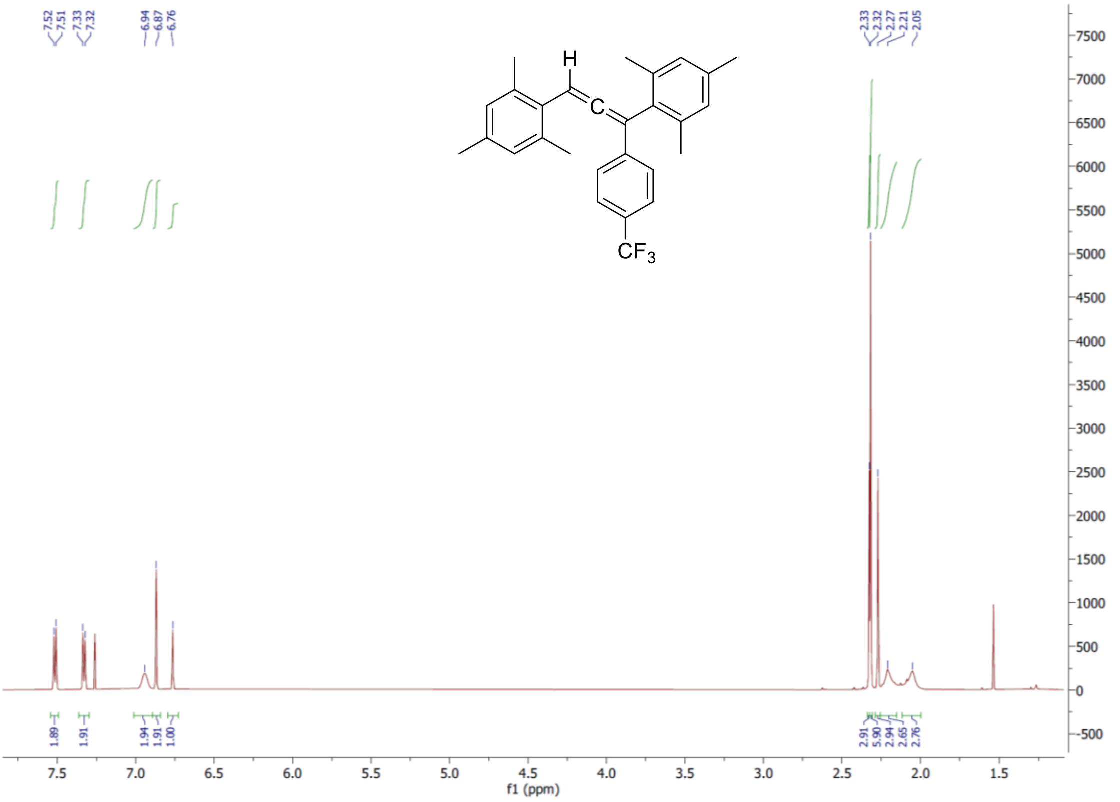

^{13}C NMR spectrum of **3i**

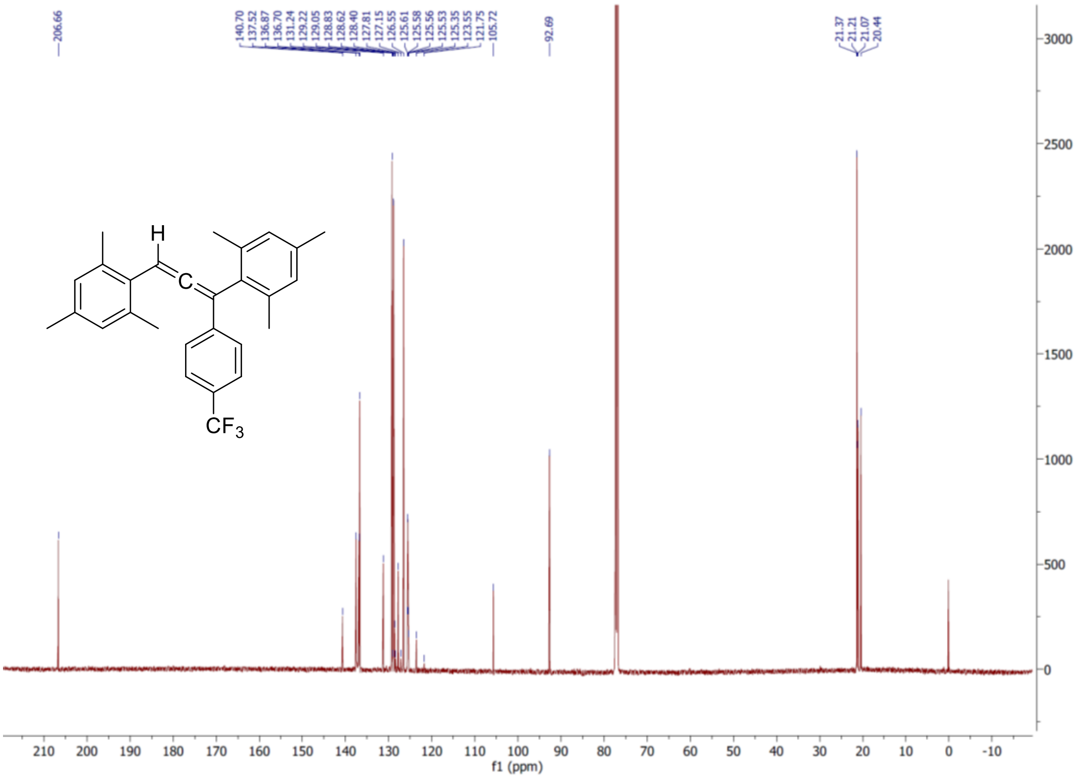

^{1}H NMR spectrum of **3j**

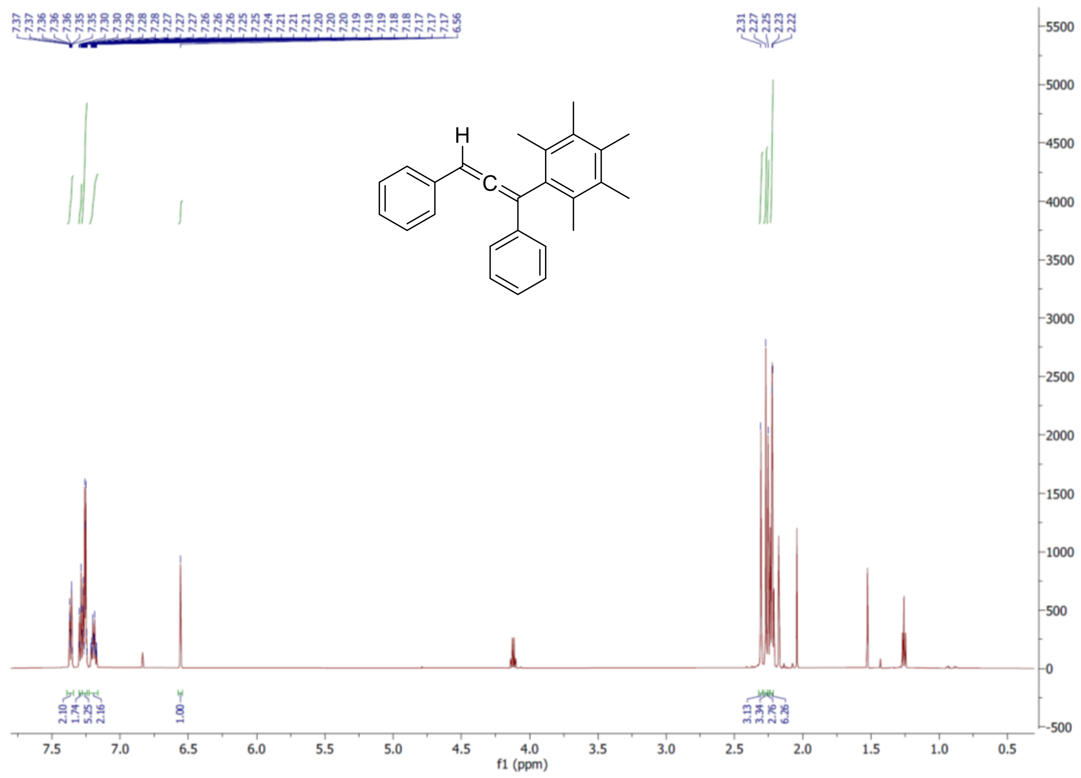

^{13}C NMR spectrum of **3j**

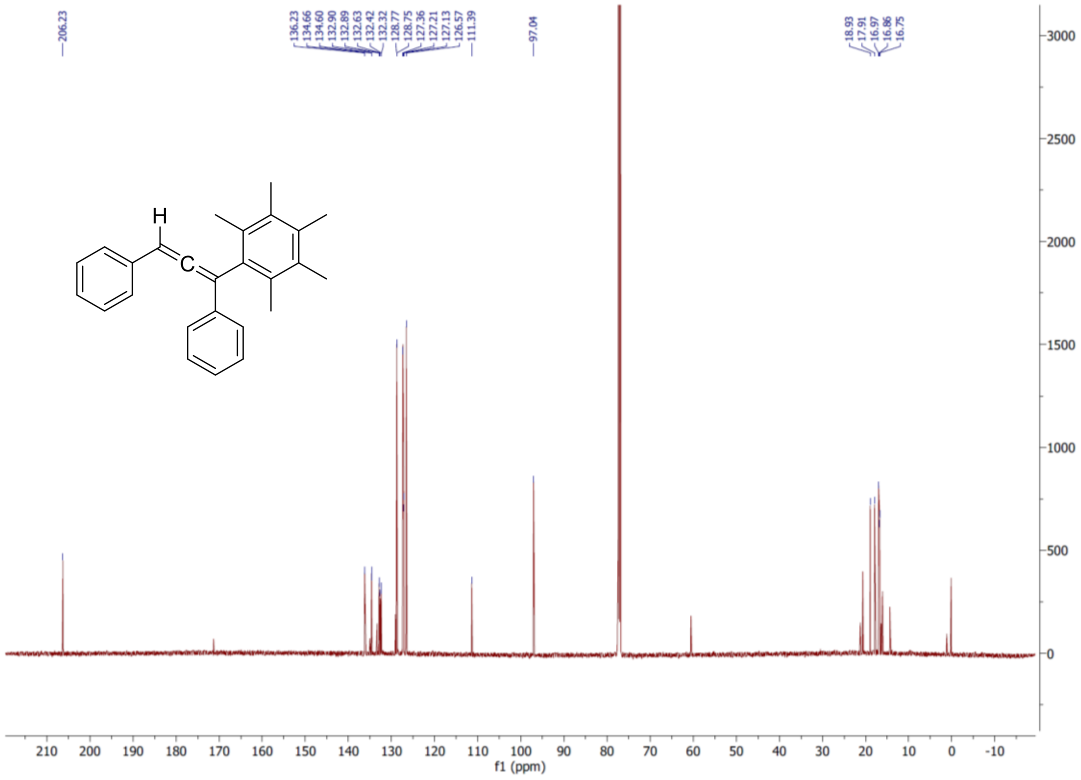

^{1}H NMR spectrum of **4a**

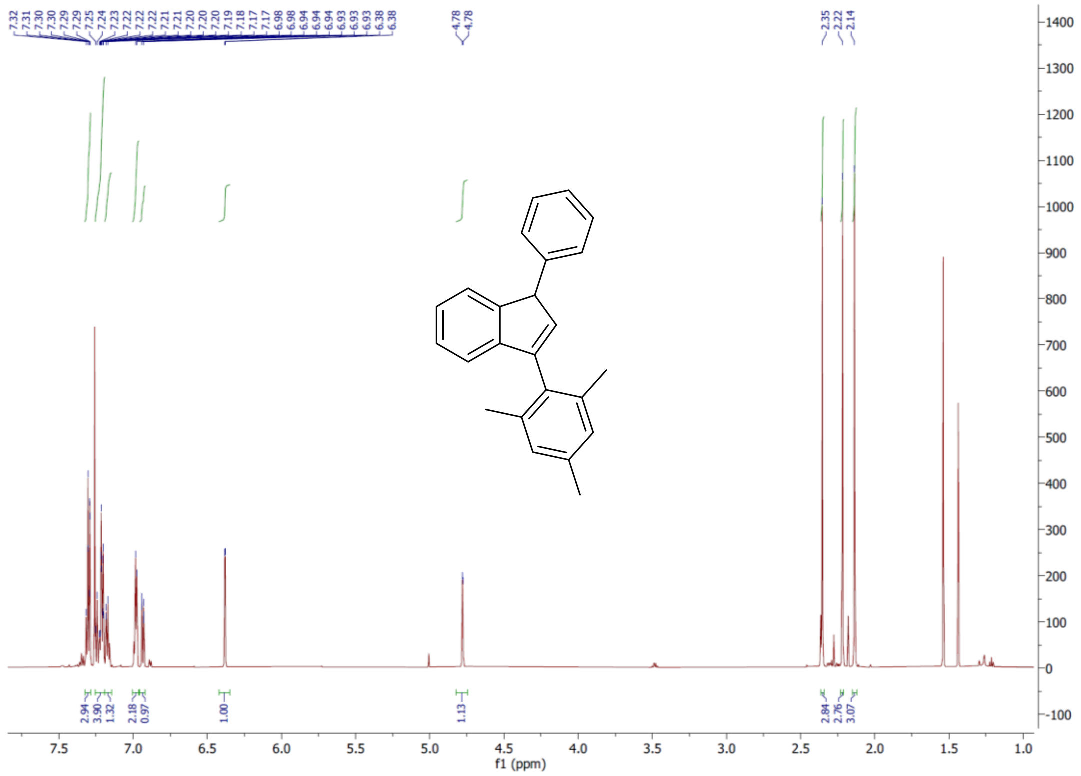

^{1}H NMR spectrum of **4b**

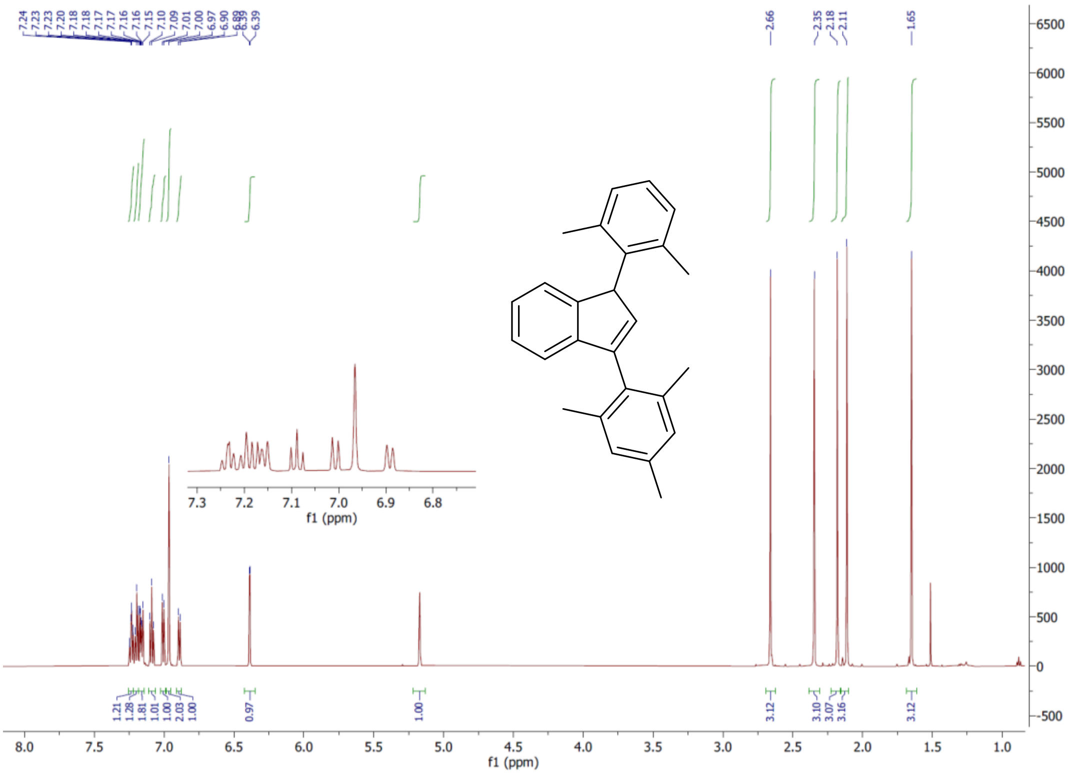

^{13}C NMR spectrum of **4b**

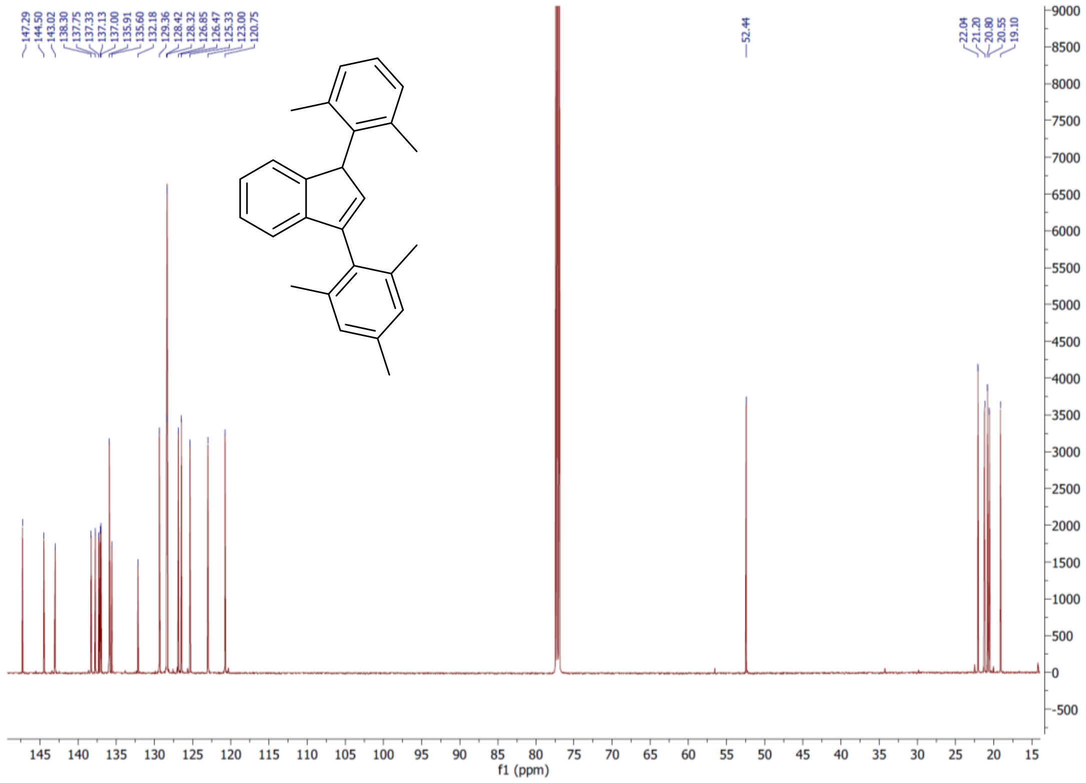

^{1}H NMR spectrum of **4c**

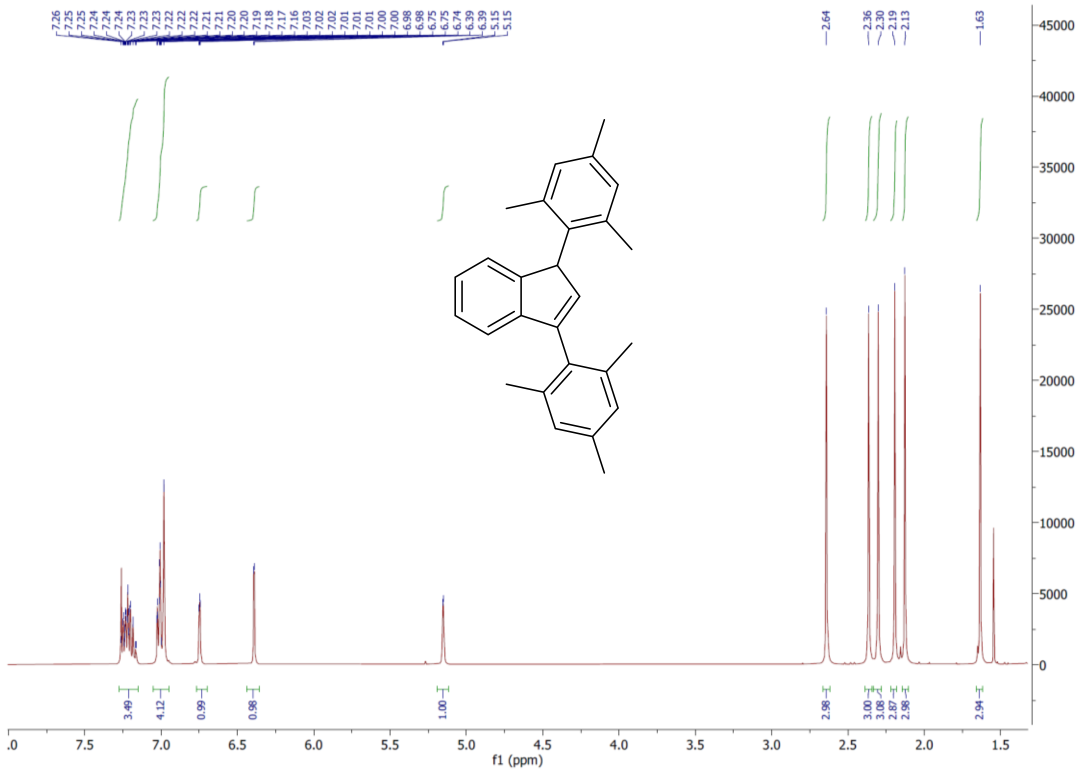

^{1}H NMR spectrum of **4d** + **2d** (≈ 1:1 mixture)

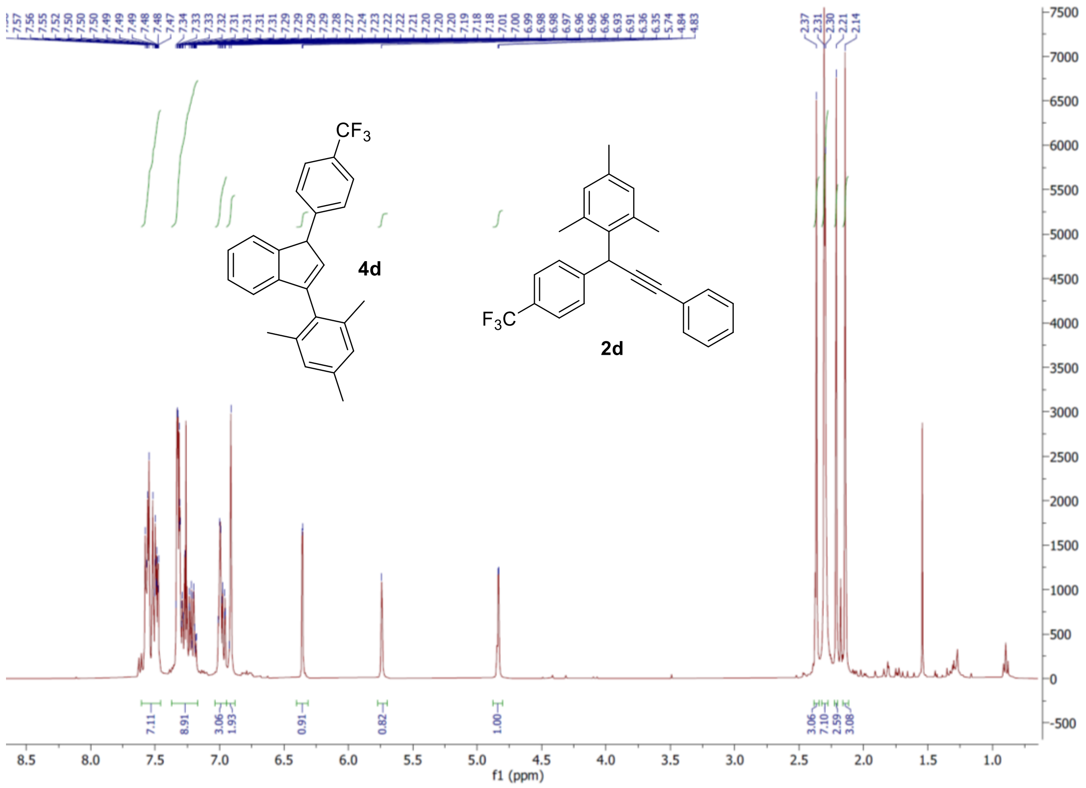

^{1}H NMR spectrum of **4j**

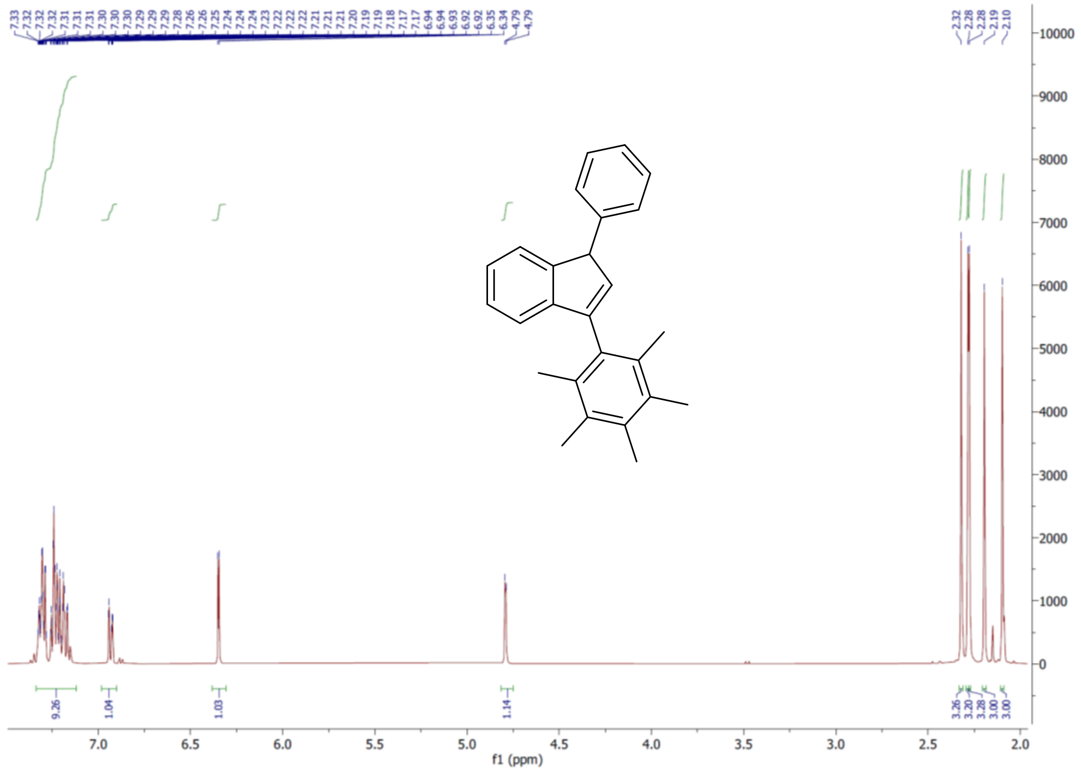

^{1}H NMR spectrum of **4k**

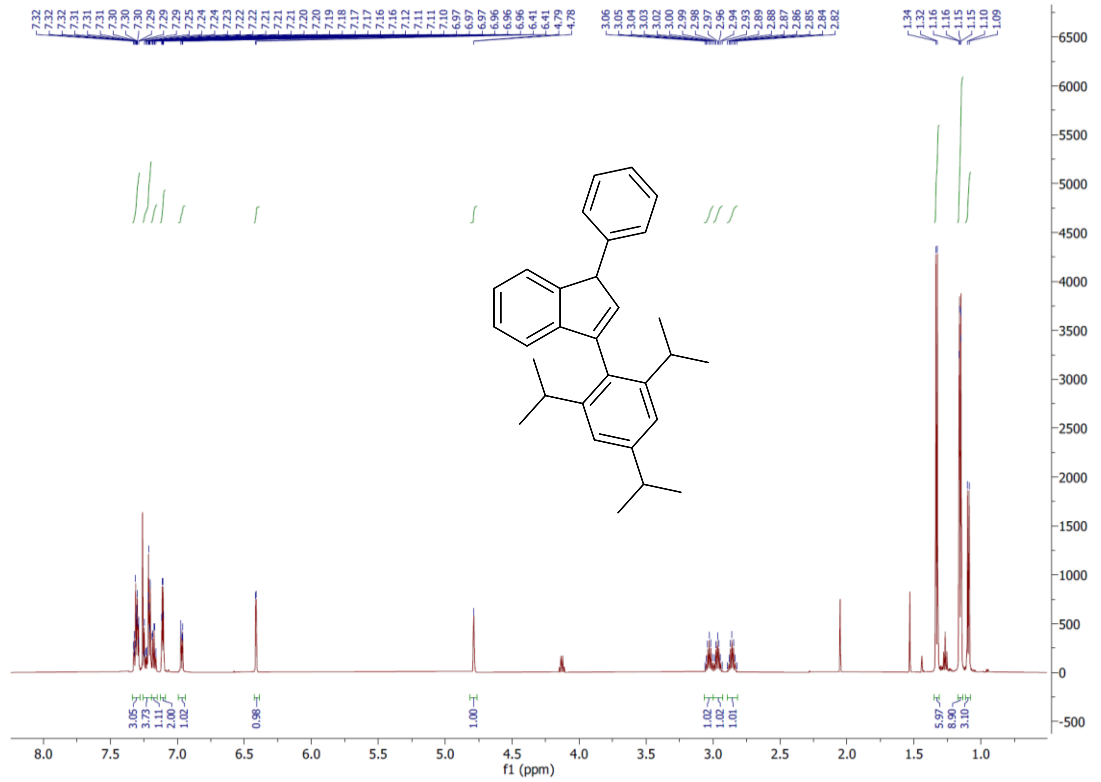

^{1}H NMR spectrum of **4a-Br**

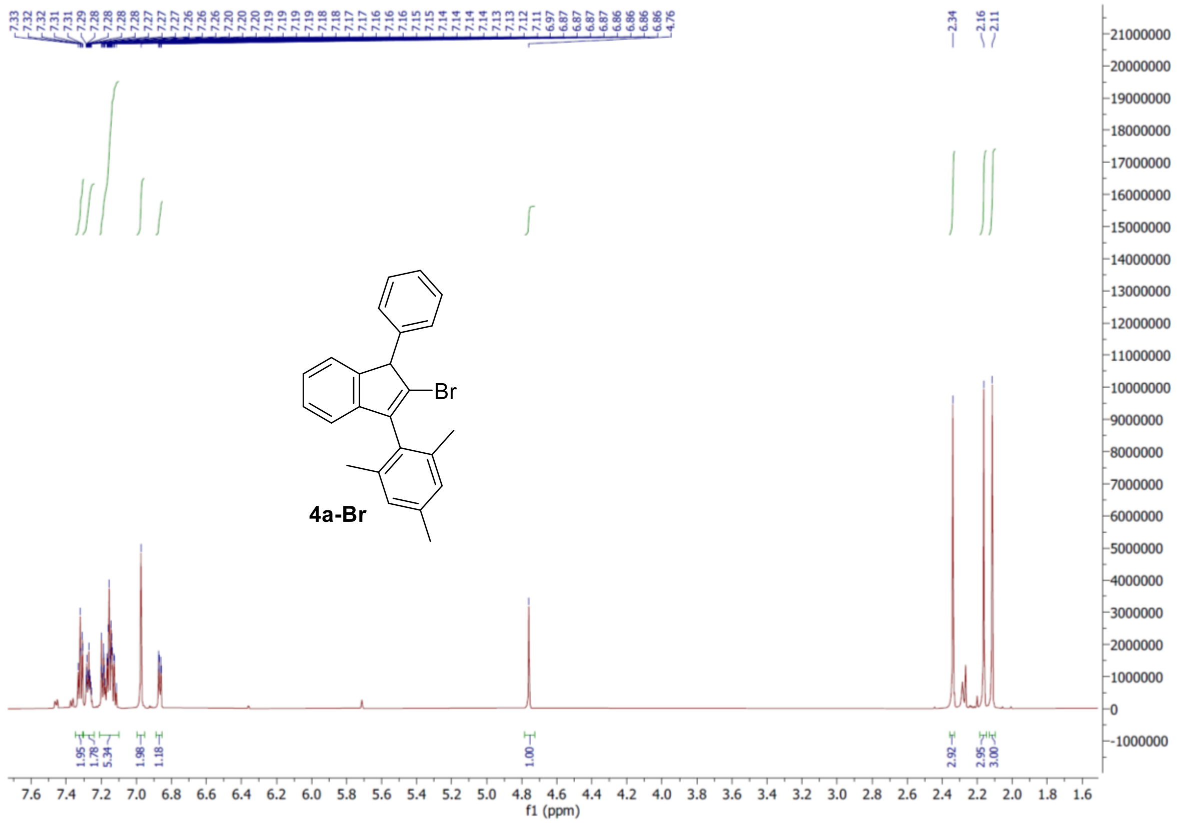

^{13}C NMR spectrum of **4a-Br**

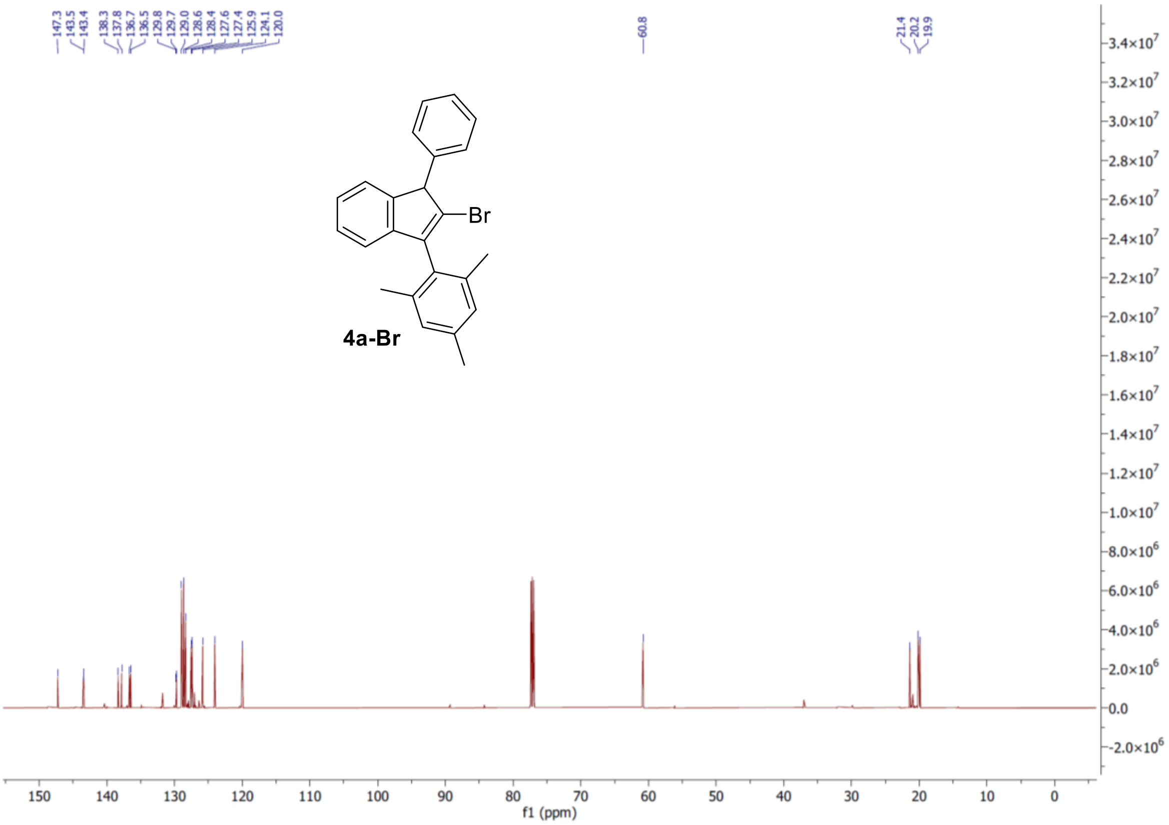

^{1}H NMR spectrum of **4d-Br**

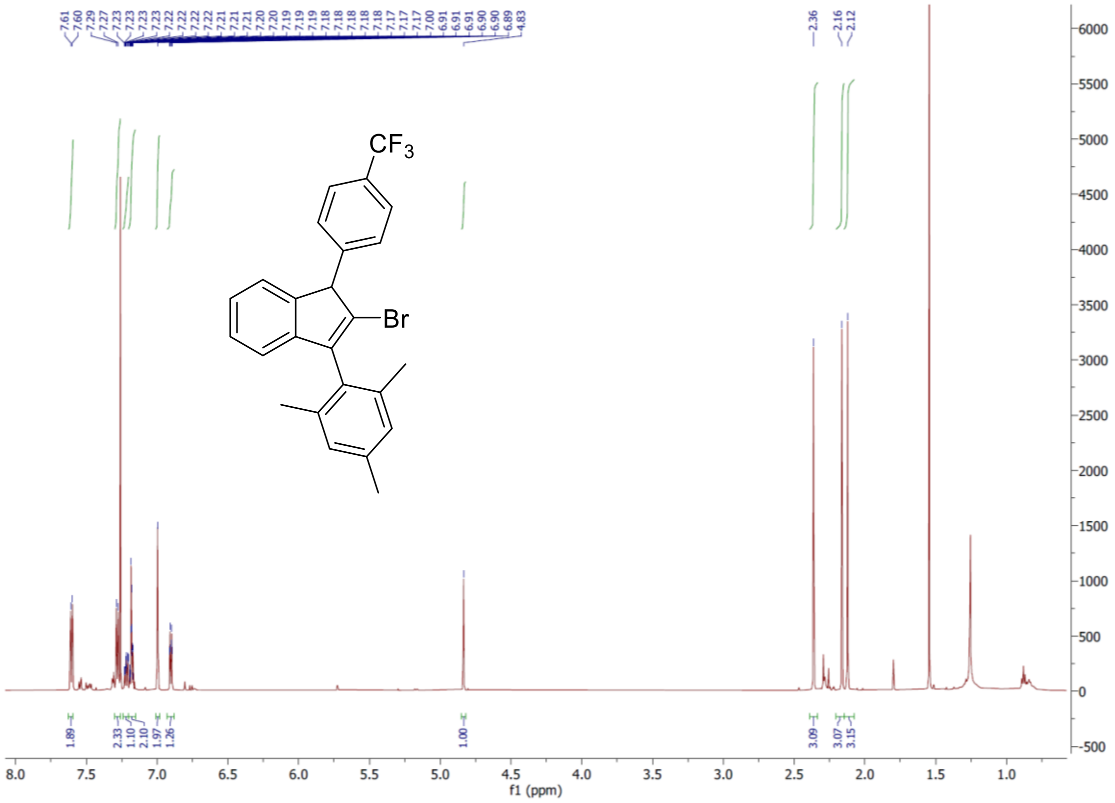

^{13}C NMR spectrum of **4d-Br**

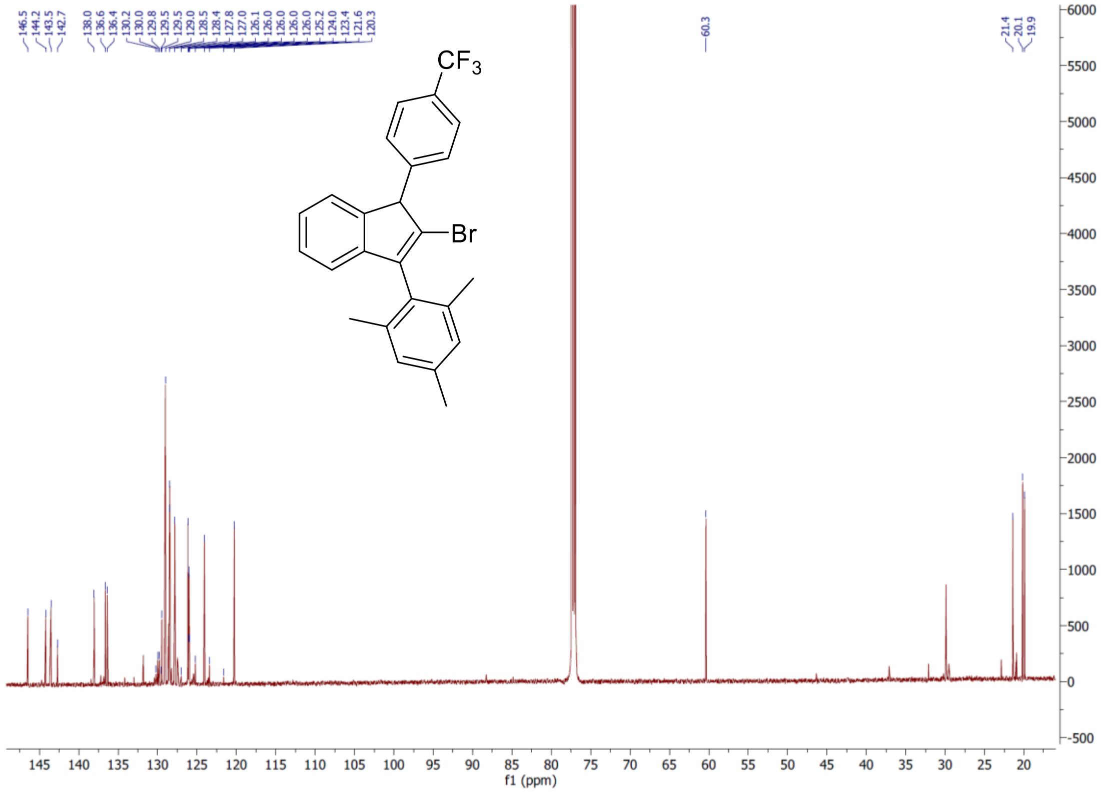

^{1}H NMR spectrum of **4a-I**

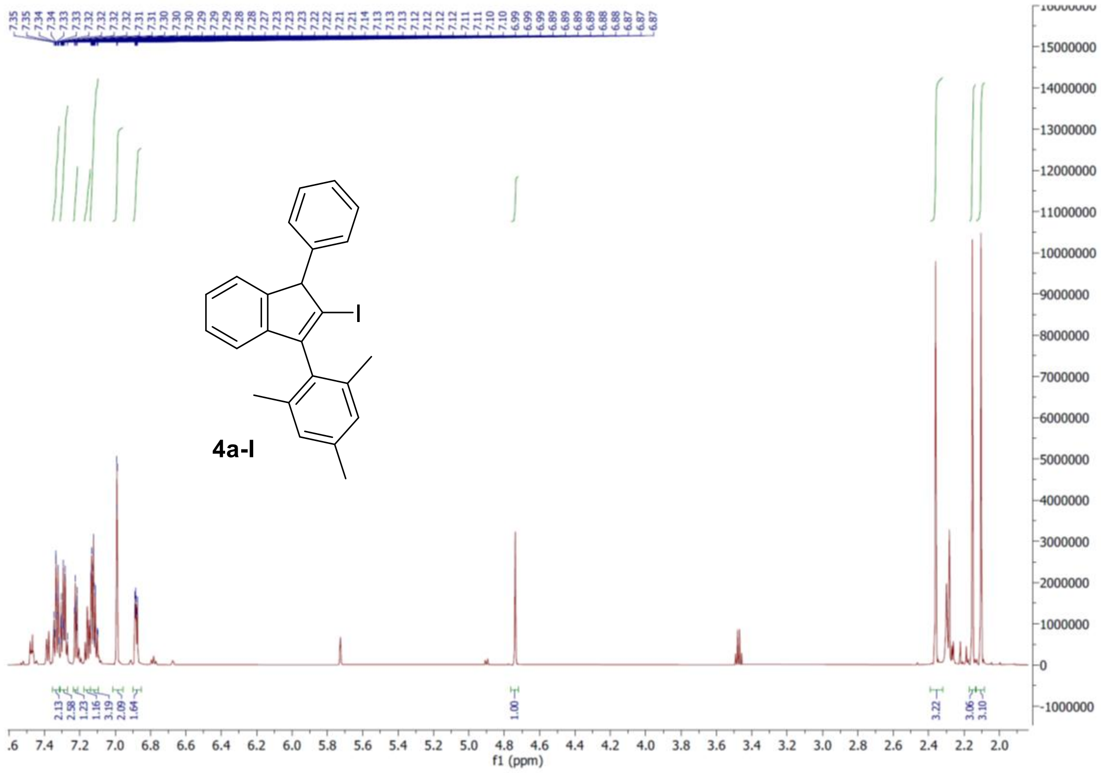

^{13}C NMR spectrum of **4a-I**

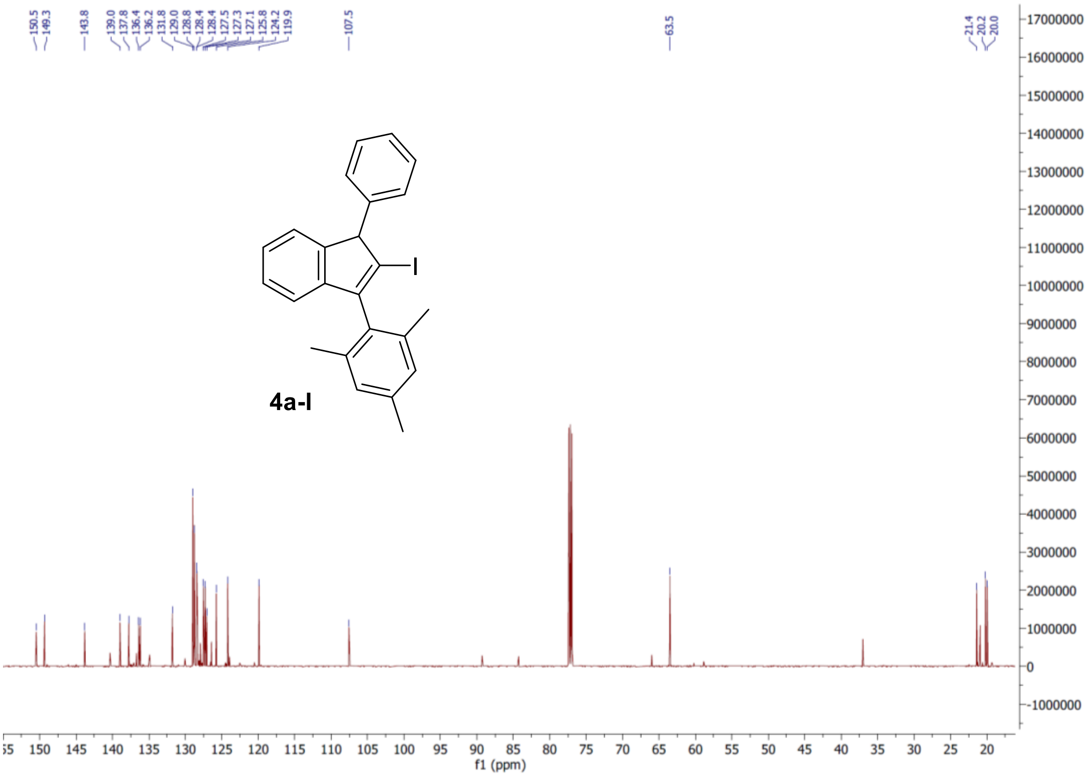

^{1}H NMR spectrum of **4j-I**

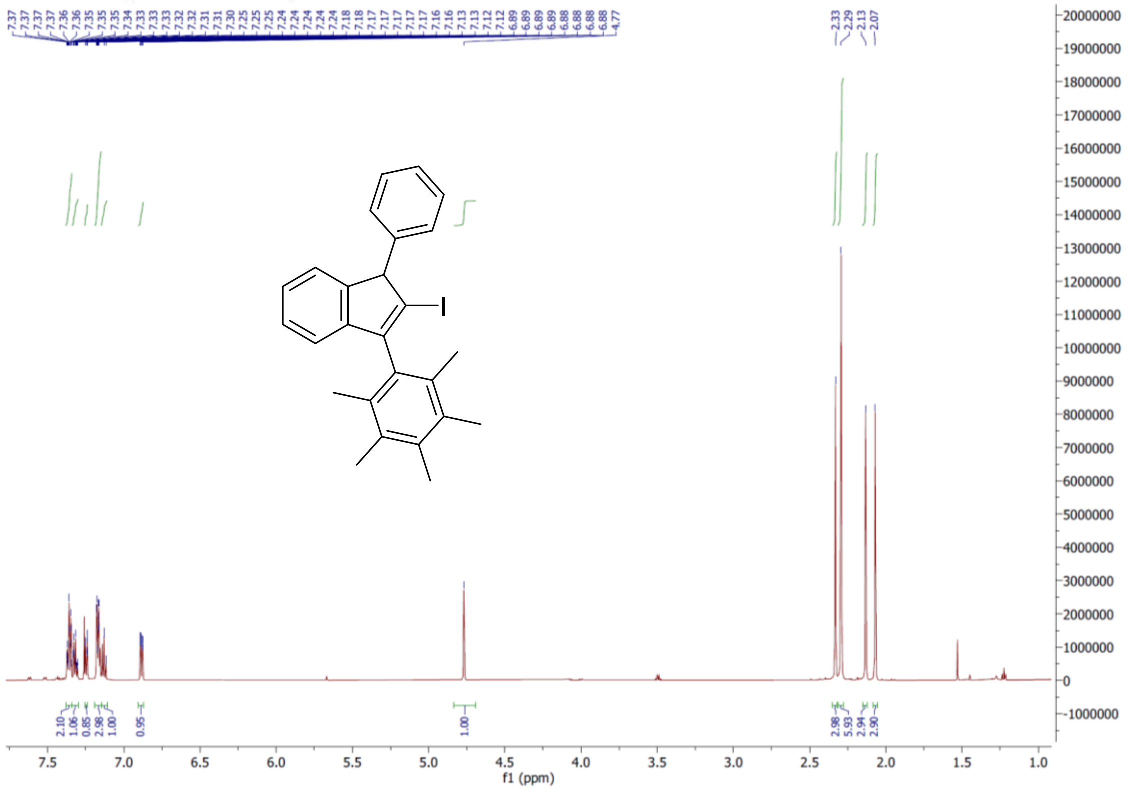

^{13}C NMR spectrum of **4j-I**

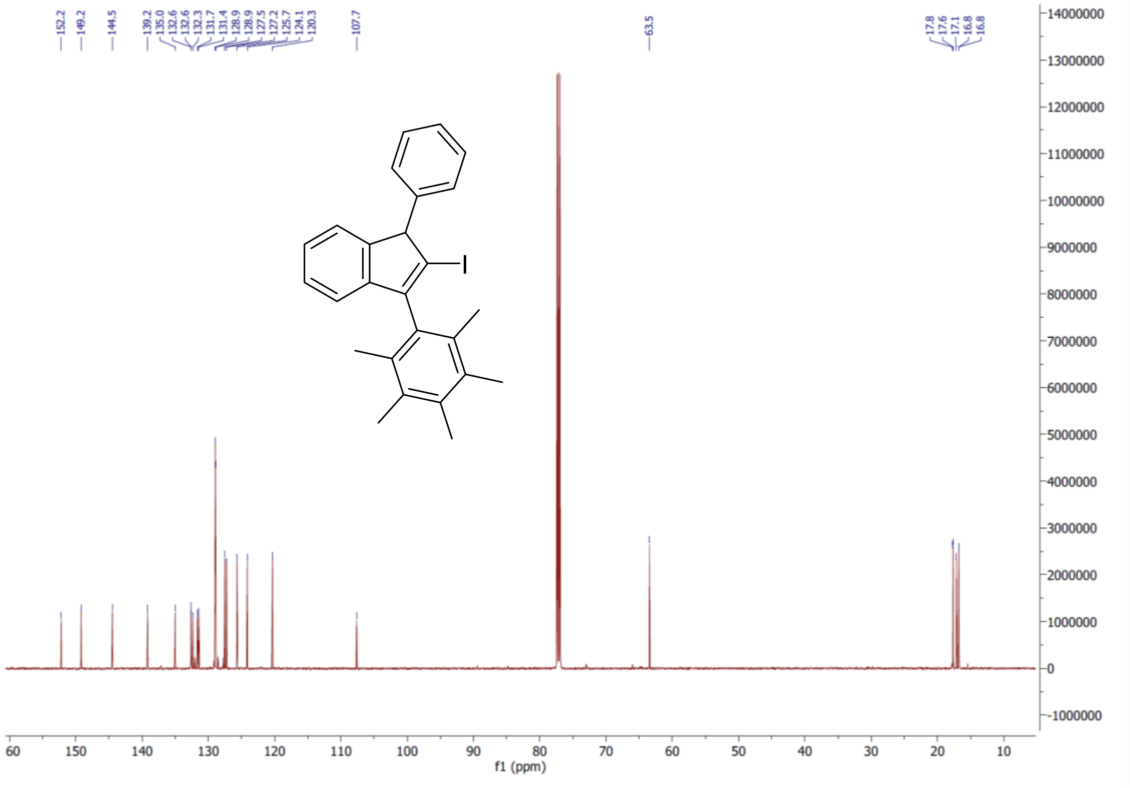

^{1}H NMR spectrum of **5a** (10:1 *E/Z* isomer mixture)

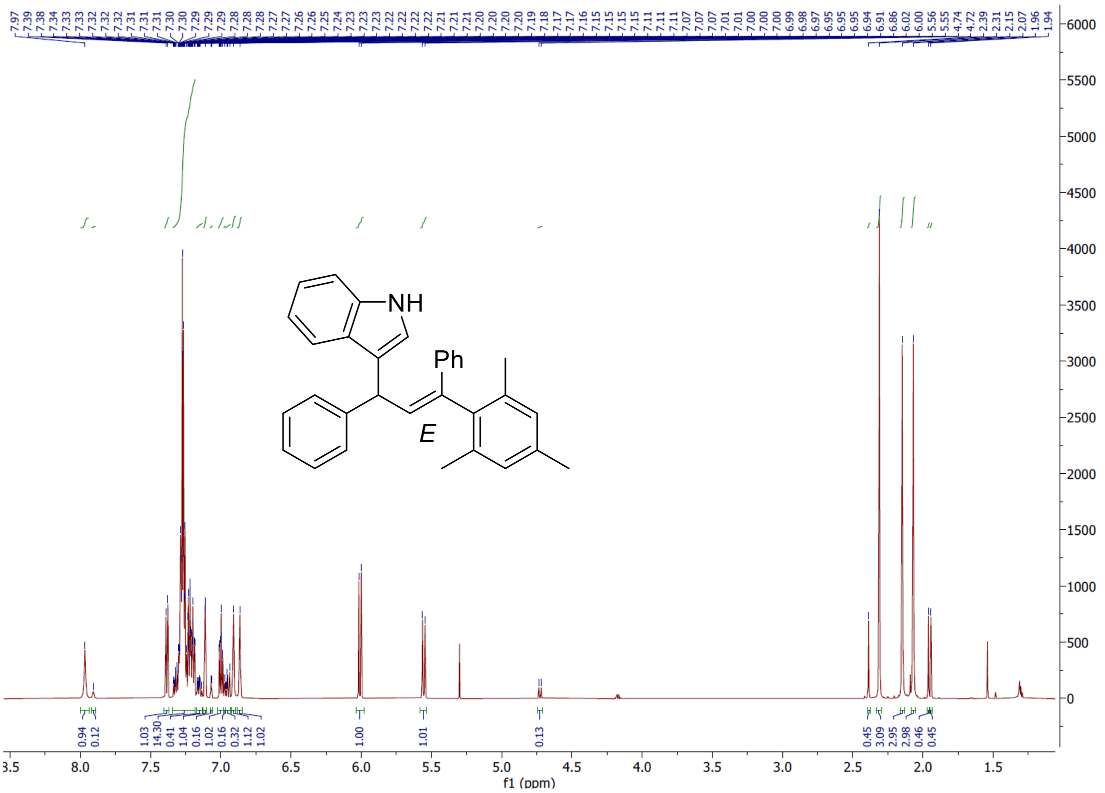

-

^{13}C NMR spectrum of **5a** (10:1 *E/Z* isomer mixture)

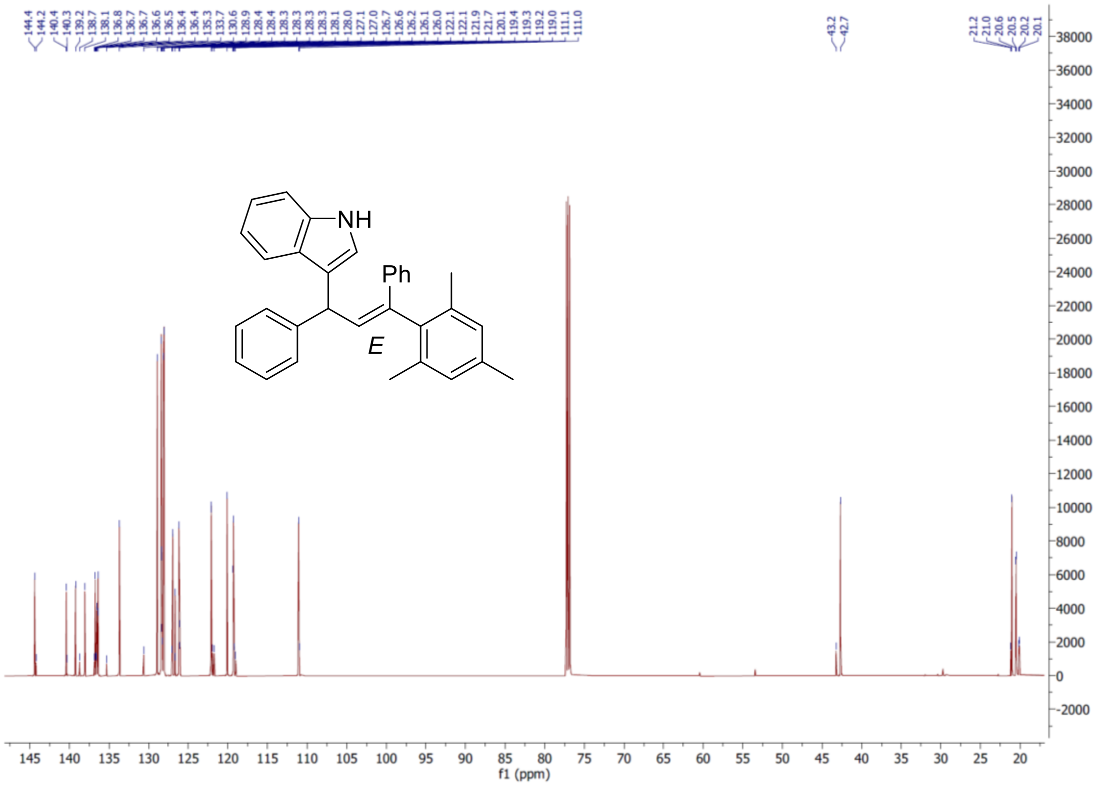

^{1}H NMR spectrum of **5b**; main *E*-isomer

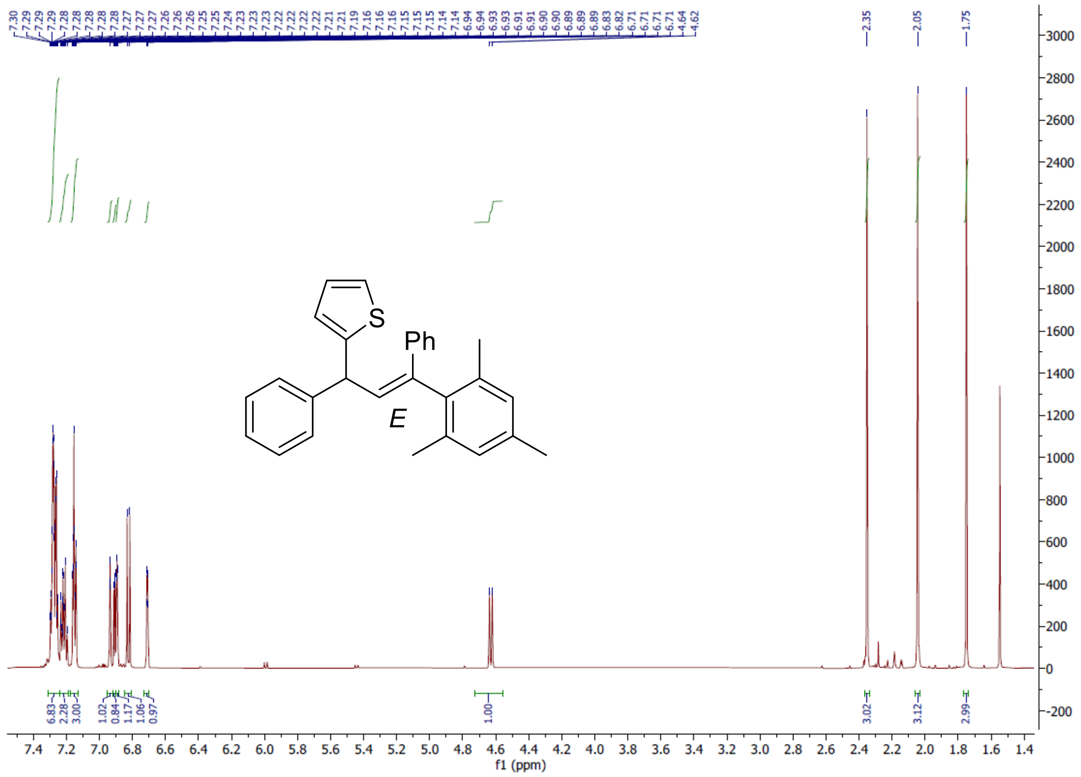

^{13}C NMR spectrum of **5b**; main *E*-isomer

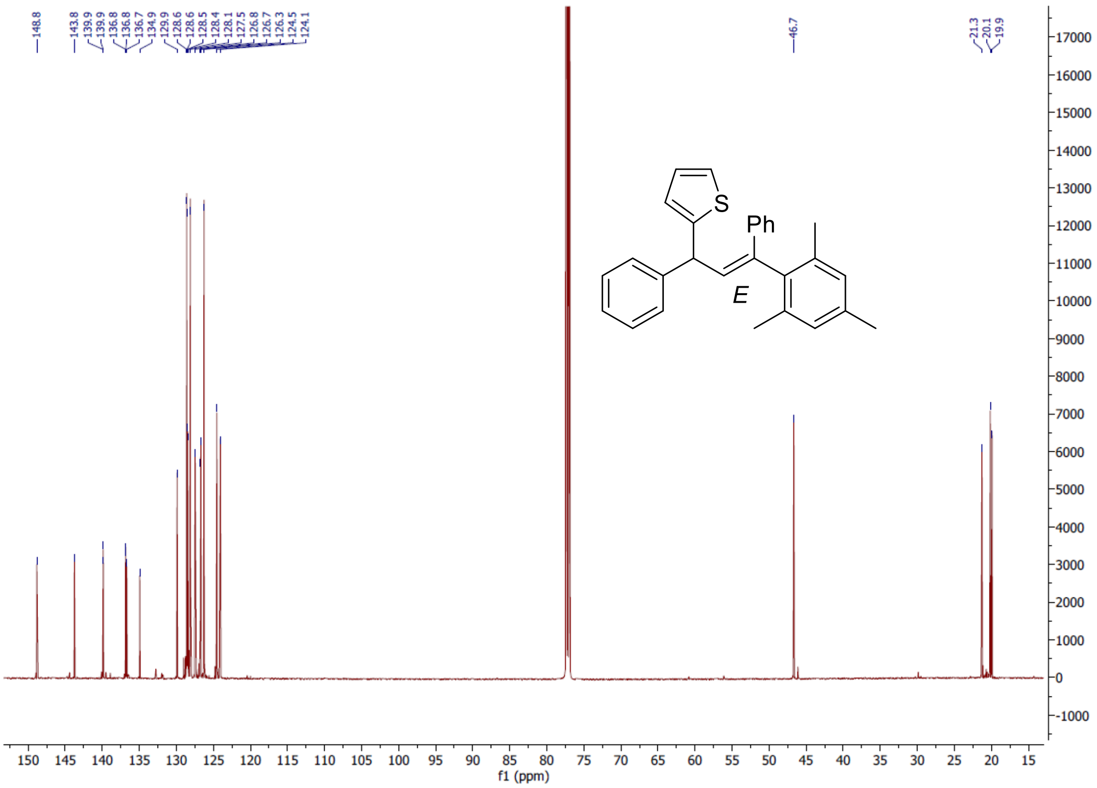

^{1}H NMR spectrum of **5b**; minor *Z*-isomer (in a 3:1 mixture of *E*/*Z* isomers)

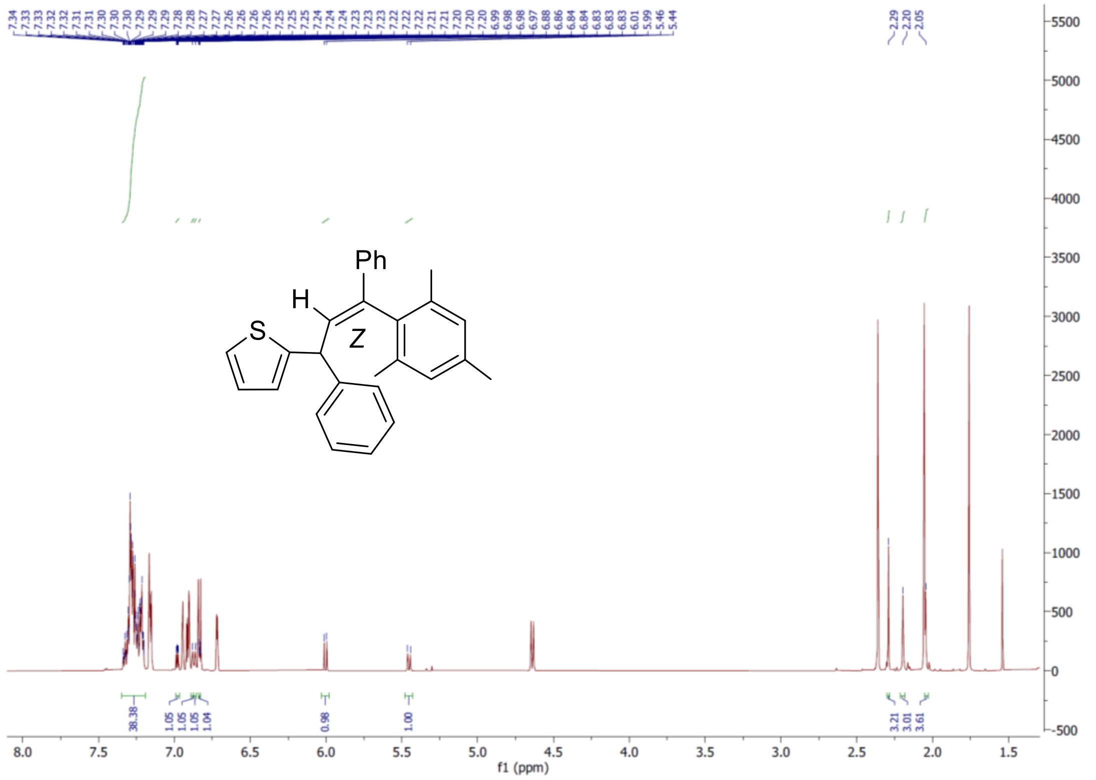

^{13}C NMR spectrum of **5b**; minor *Z*-isomer (in a 3:1 mixture of *E*/*Z* isomers)

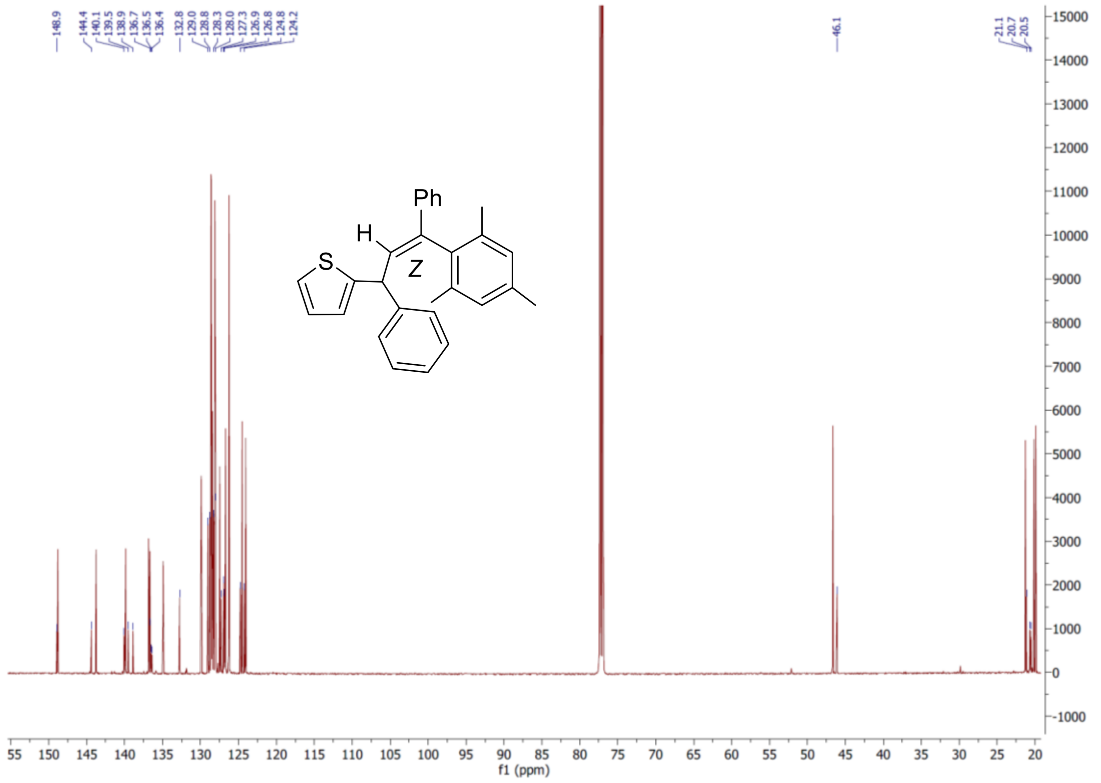

^{1}H NMR spectrum of **5b-bis**

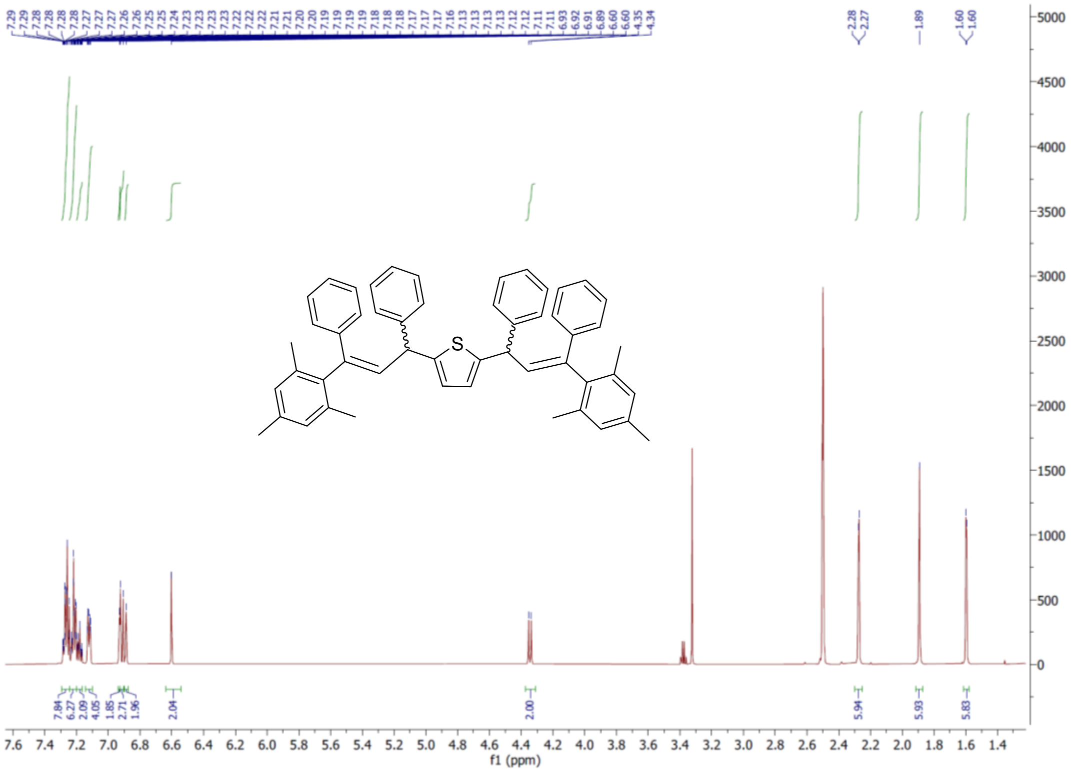

^{13}C NMR spectrum of **5b-bis**

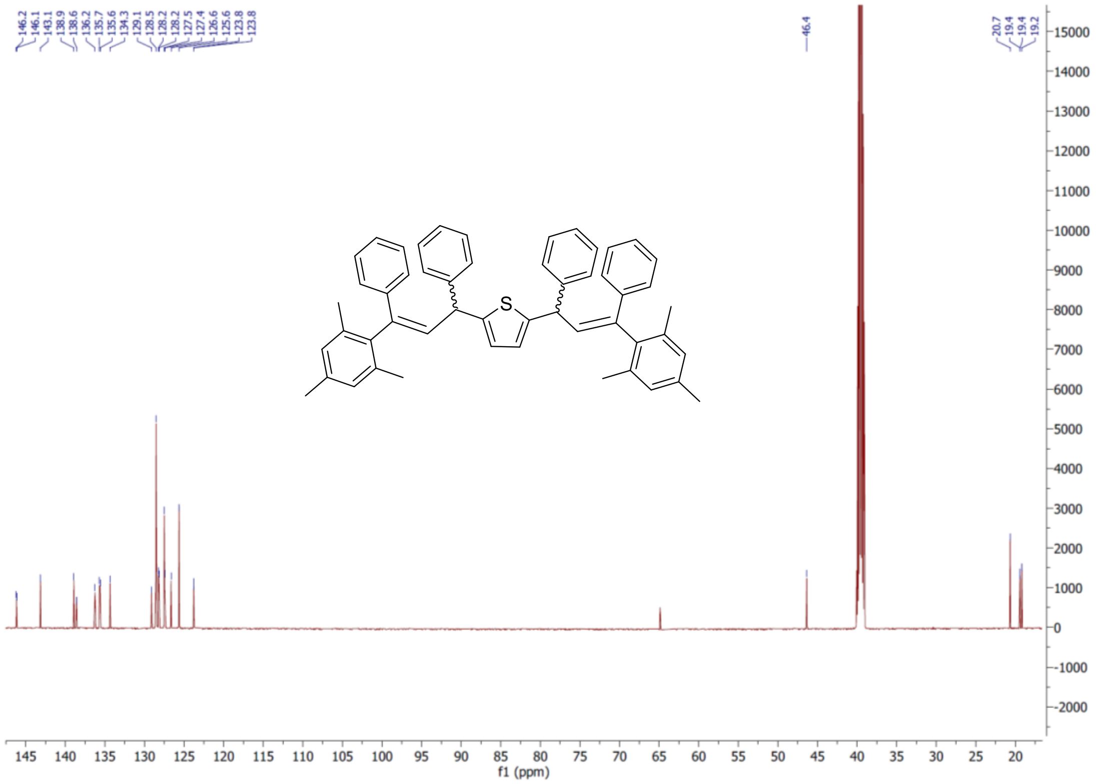

^{1}H NMR spectrum of **5c-bis**

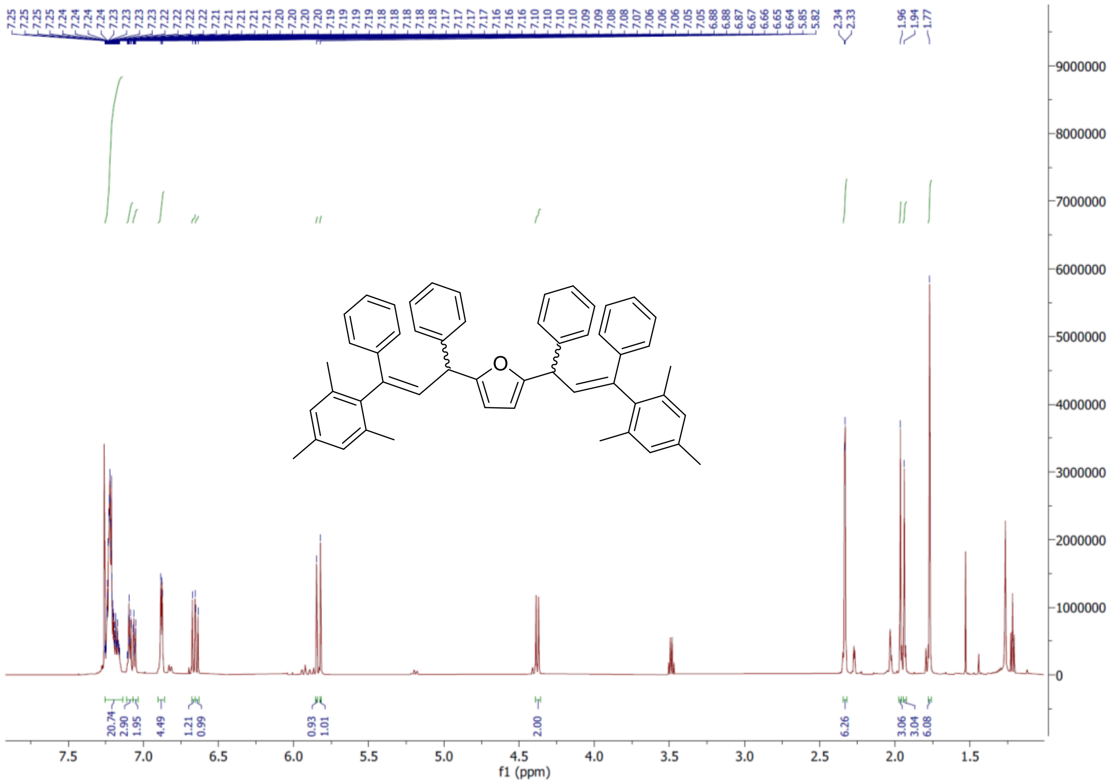

^{13}C NMR spectrum of **5c-bis**

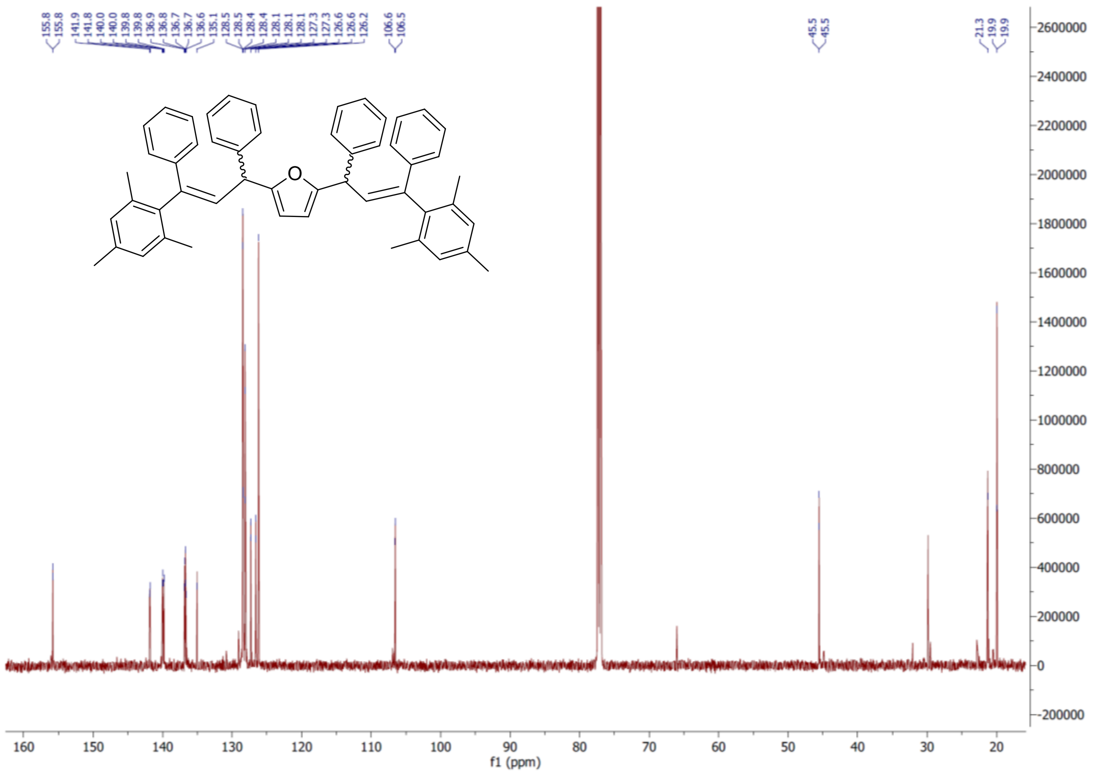

^{1}H NMR spectrum of **5d**

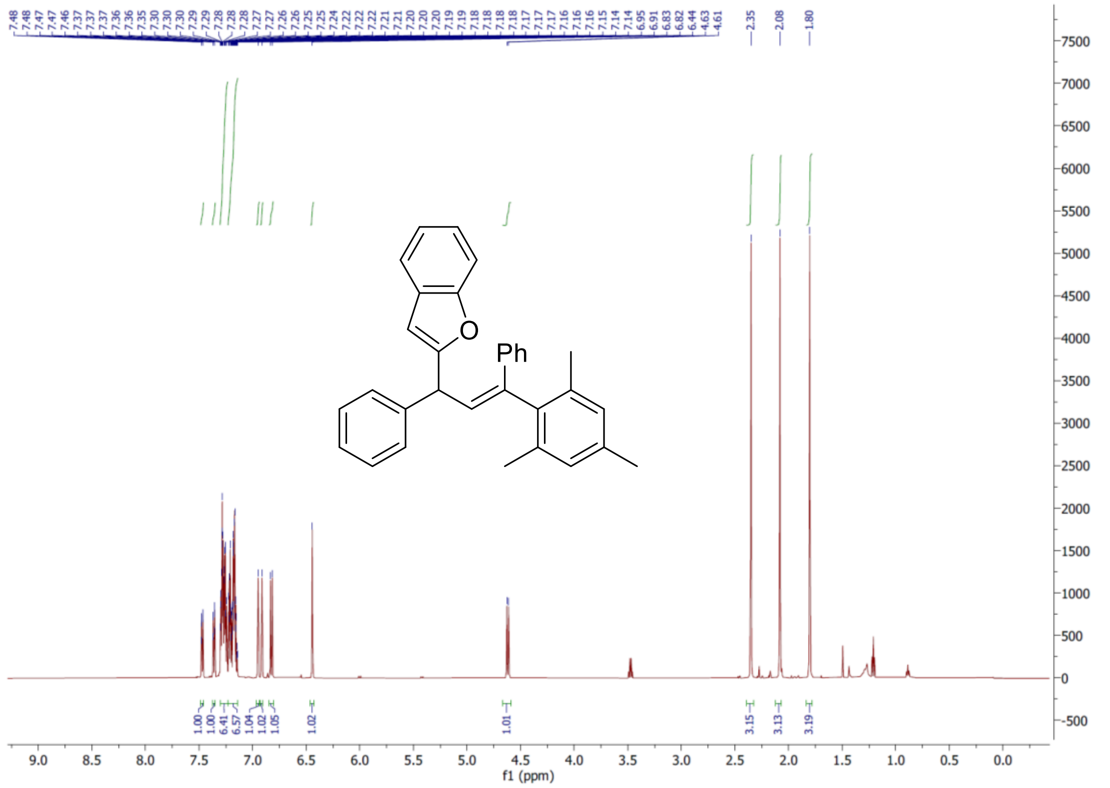

^{13}C NMR spectrum of **5d**

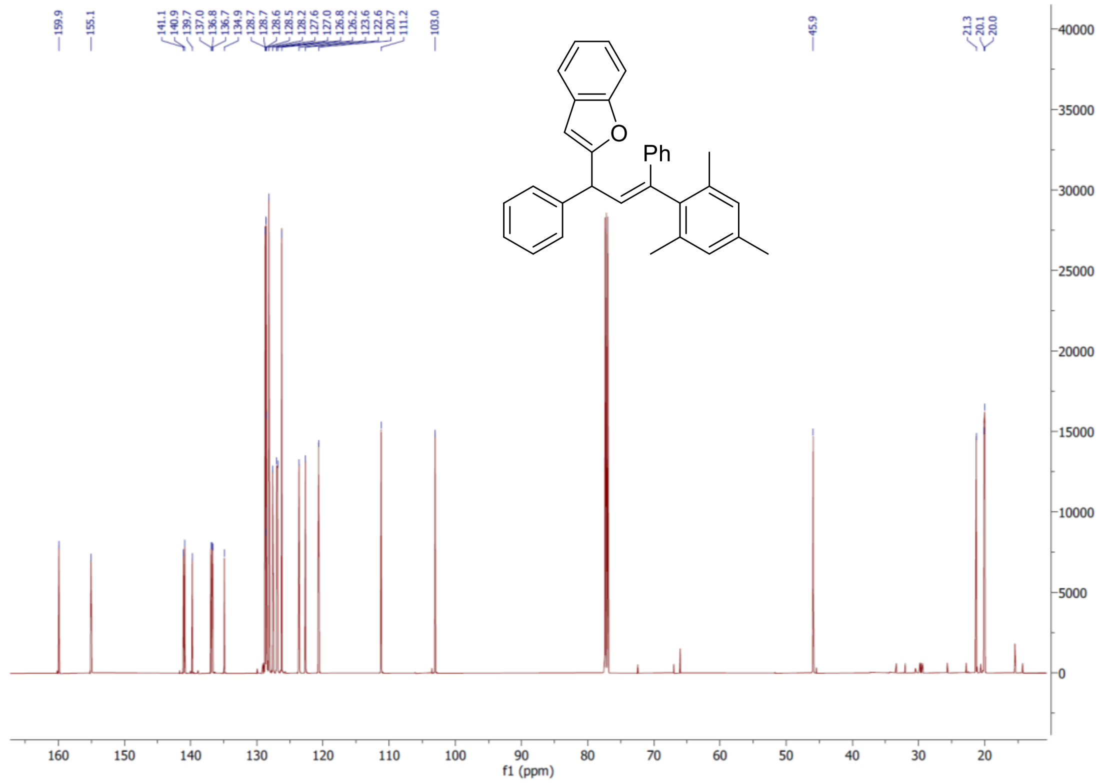

^{1}H NMR spectrum of **5e²** (3:2 mixture of 2-/3-regioisomers **5e²** / **5e³**)

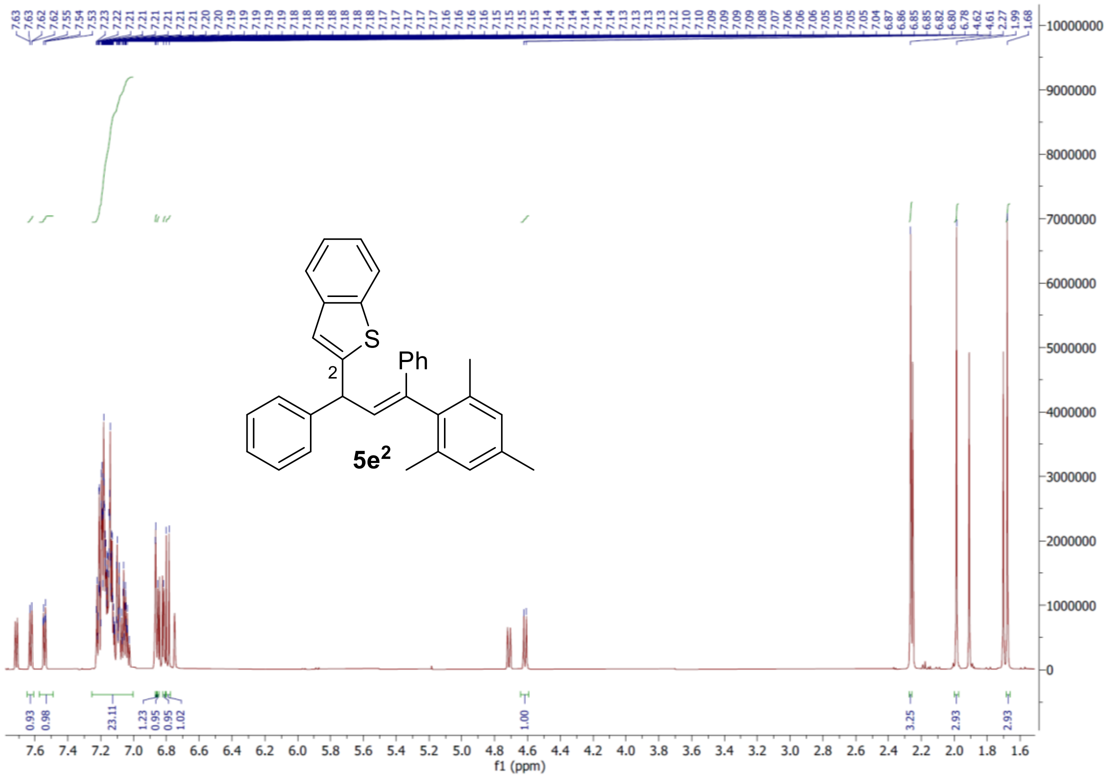

^{13}C NMR spectrum of **5e²** (3:2 mixture of 2-/3-regioisomers **5e²** / **5e³**)

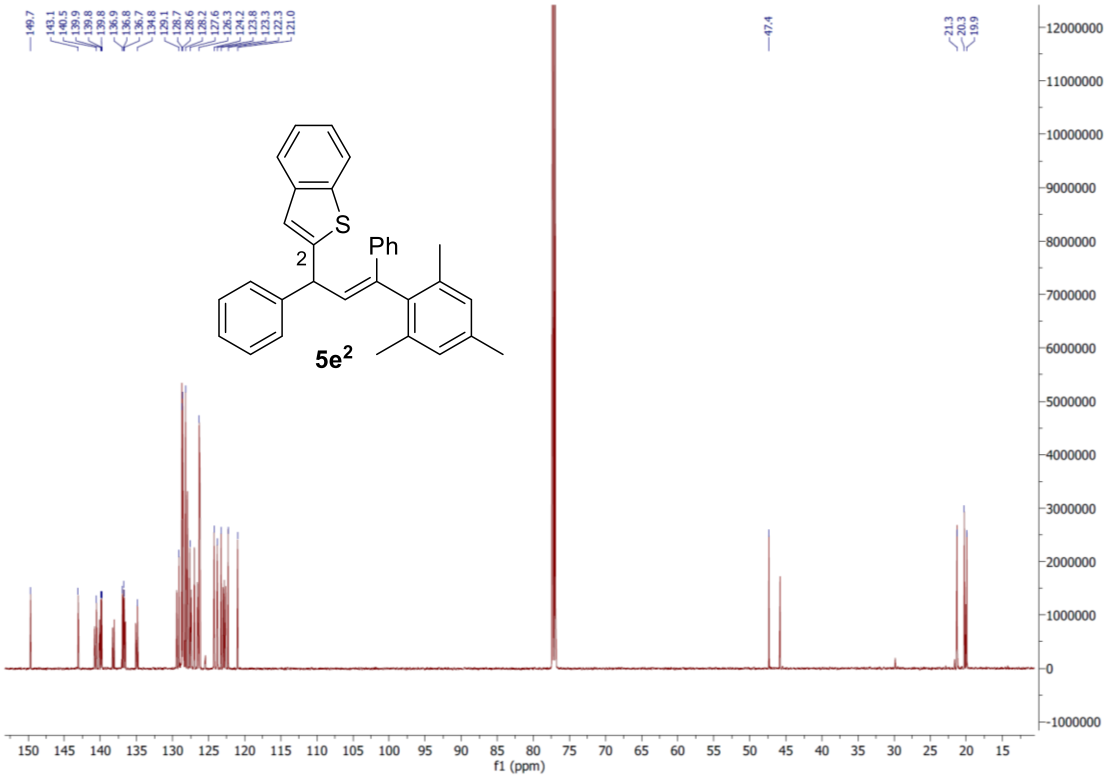

^{1}H NMR spectrum of **5e³**

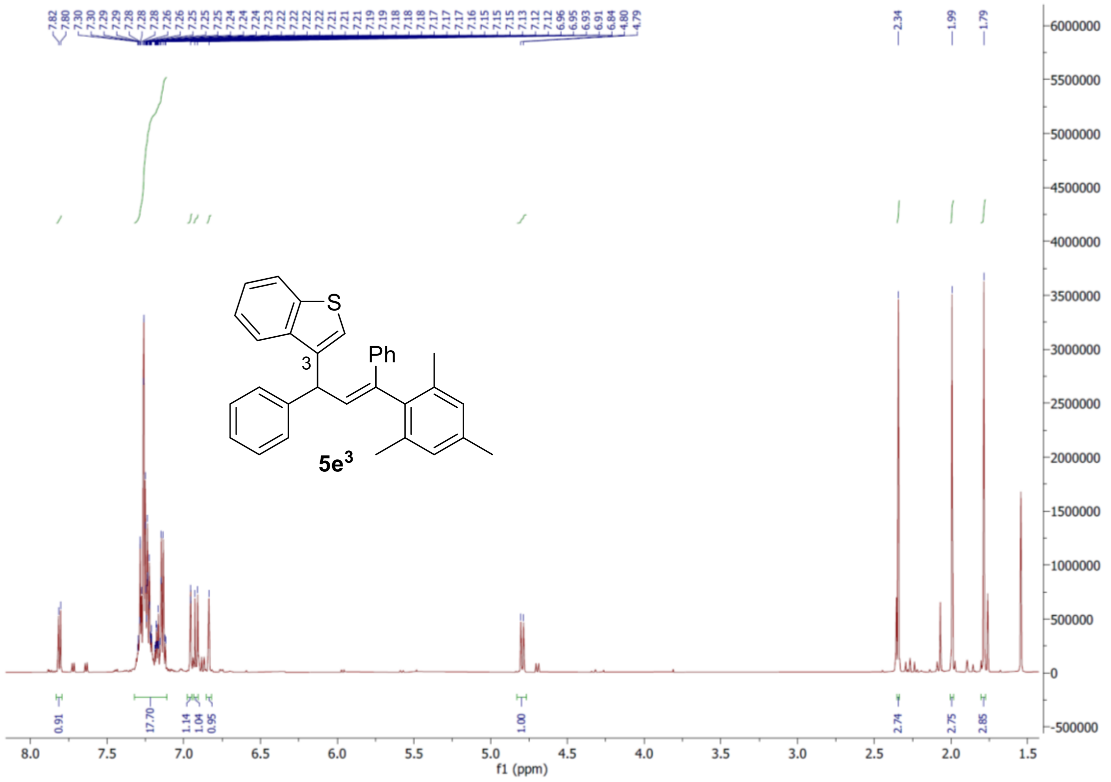

^{13}C NMR spectrum of **5e³**

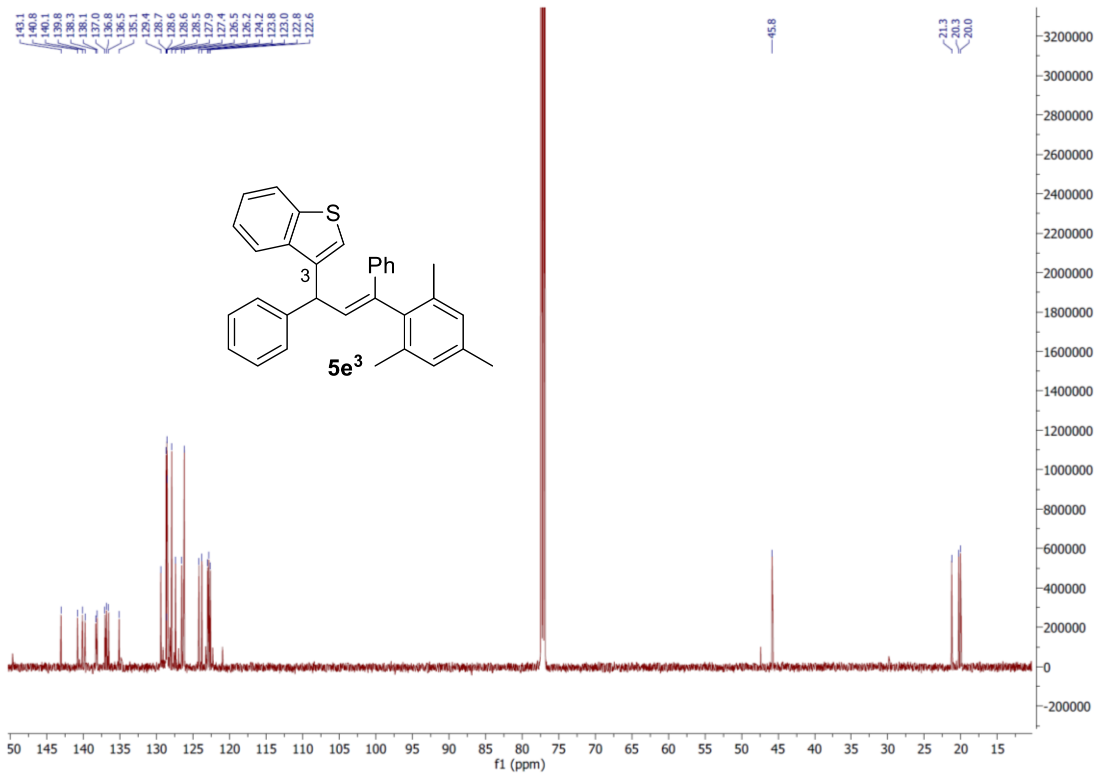

^{1}H NMR spectrum of **5f**

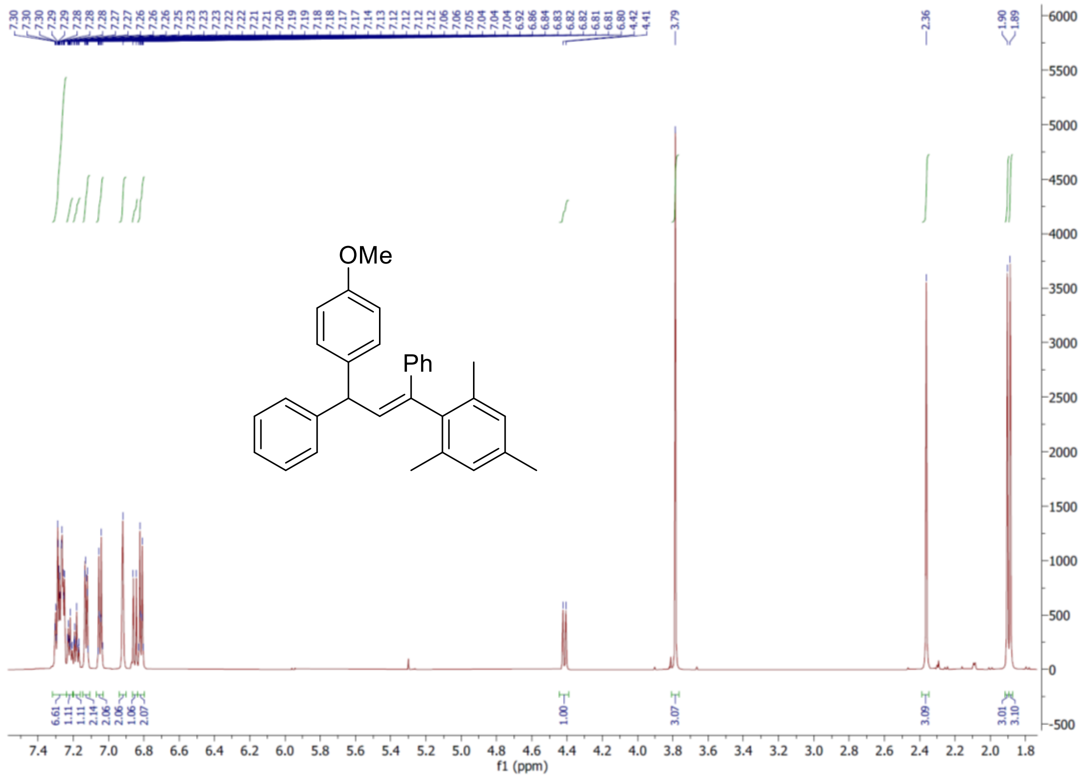

^{13}C NMR spectrum of **5f**

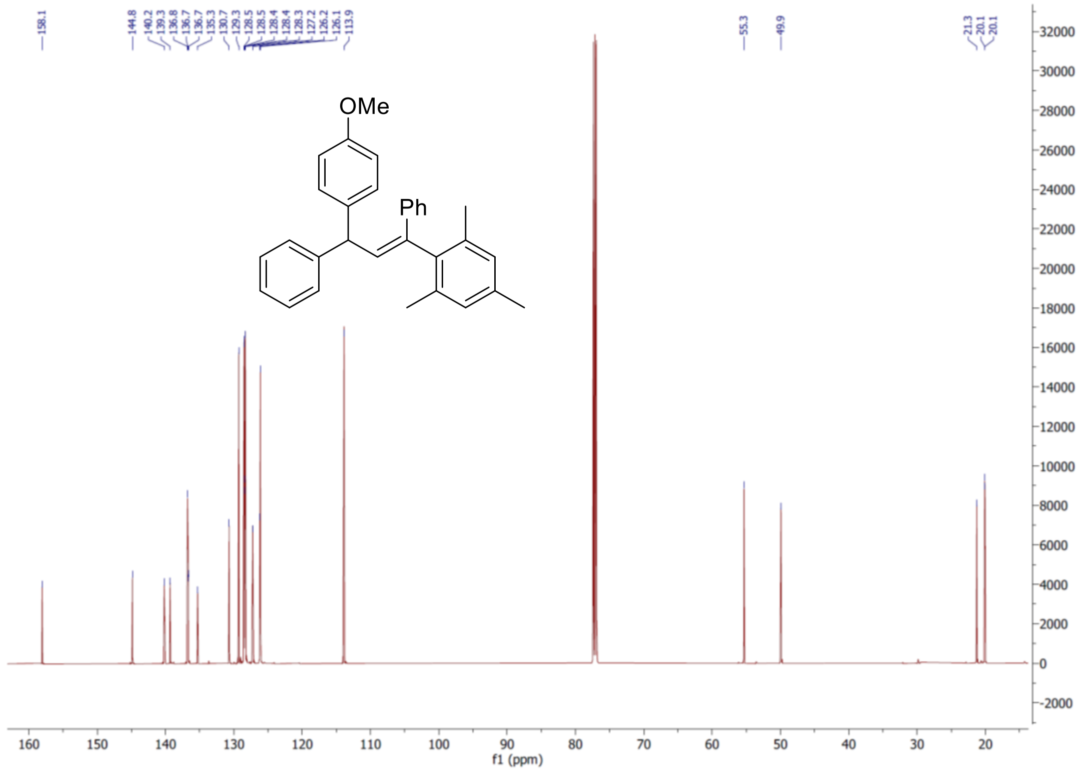

^{1}H NMR spectrum of **5g** (7:3 mixture of **5g** and 1,3,5-trimethoxybenzene)

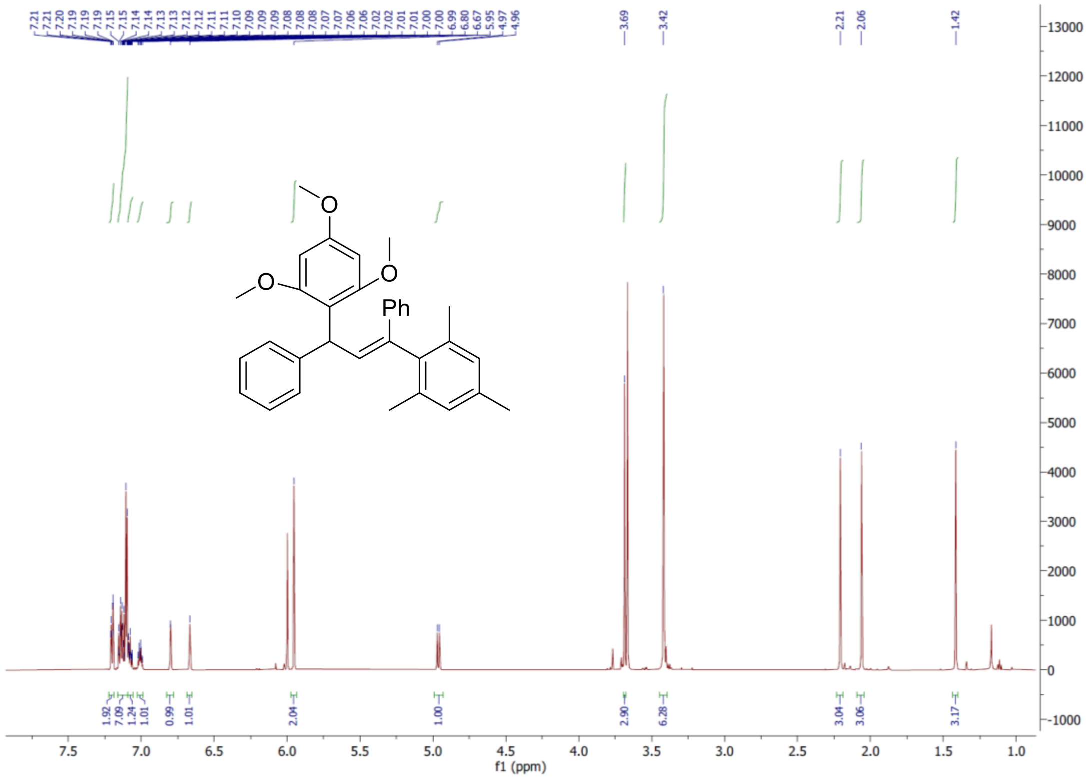

^{13}C NMR spectrum of **5g** (7:3 mixture of **5g** and 1,3,5-trimethoxybenzene)

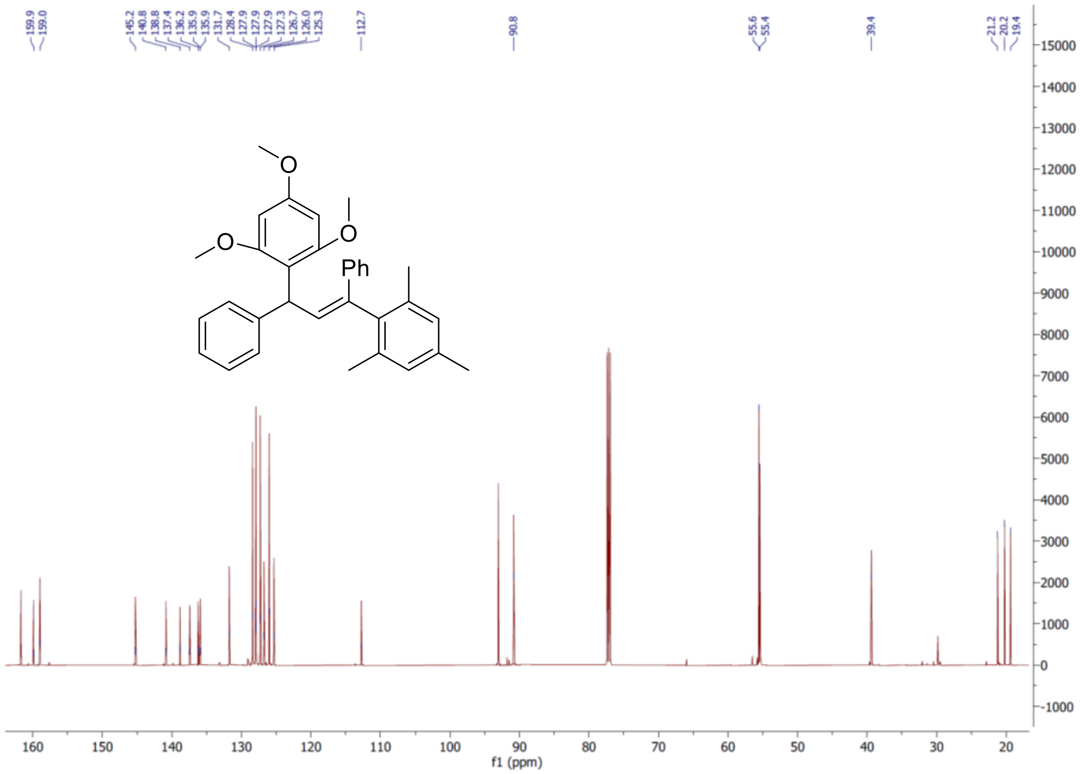

Specific assignments (^{1}H, ^{13}C NMR) of products 2-4:

(based on 2D NMR studies; COSY, HSQC, HMBC, NOESY)

<u>C3 Substitution products 2:</u>

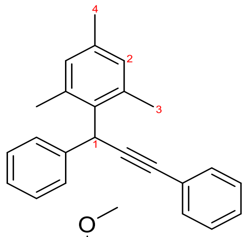

(3-Mesitylprop-1-yne-1,3-diyl)dibenzene (**2a**):
^{1}H NMR (400 MHz, $CDCl_3$) δ (ppm): 7.46 (m, 2H, Ph), 7.38 (d, *J* = 7.7, 2H, Ph), 7.25-7.31 (m, 4H, Ph), 7.20 (m, 2H, Ph), 6.88 (s, 2H, H2), 5.72 (s, 1H, H1), 2.29 (bs, 6H, H3), 2.28 (bs, 3H, H4).

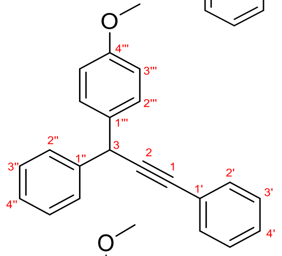

(3-(4-Methoxyphenyl)prop-1-yne-1,3-diyl)dibenzene (**2l**):
^{1}H NMR (600 MHz, $CDCl_3$) δ (ppm): 7.44-7.48 (m, 2H, H2'), 7.40-7.44 (m, 2H, H2''), 7.25-7.36 (m, 7H, H3', H4', H3'' and H2'''), 7.18-7.24 (m, 2H, H4''), 6.82-6.87 (m, 2H, H3'''), 5.16 (s, 1H, H3), 3.75 (s, 3H, OMe4''').

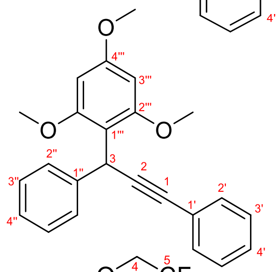

(3-(2,4,6-Trimethoxyphenyl)prop-1-yne-1,3-diyl)dibenzene (**2m**):
^{1}H NMR (600 MHz, $CDCl_3$) δ (ppm): 7.50 (d, *J* = 7.8, 2H, H2''), 7.43-7.48 (m, 2H, H2'), 7.22-7.48 (m, 5H, H3', H4' and H3''), 7.15 (t, *J* = 7.3, 1H, H4''), 6.15 (s, 2H, H3'''), 5.86 (s, 1H, H3), 3.80 (s, 6H, Me2'''), 3.78 (s, 3H, Me4''').

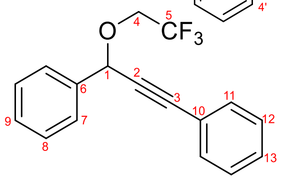

(3-(2,2,2-Trifluoroethoxy)prop-1-yne-1,3-diyl)dibenzene (**2a$^{CF3-EtO}$**):
^{1}H NMR (600 MHz, $CDCl_3$) δ (ppm): 7.60 (m, 2H, H7), 7.50 (m, 2H, H11), 7.42 (m, 2H, H8), 7.38 (m, 1H, H9), 7.36 (m, 1H, H13), 7.34 (m, 2H, H12), 5.65 (s, 1H, H1), 4.06 (dq, *J* = 12.0, 8.6, 1H, H4), 3.98 (dq, *J* = 12.0, 8.8, 1H, H4).
^{13}C NMR (150 MHz, CDCl3) δ (ppm): 137.0 (C6), 131.9 (C11), 128.98-128.99 (C9 and C13), 128.7 (C8), 128.4 (C12), 127.6 (C7), 124.05 (q, *J* = 279.0, C5), 121.9 (C10), 89.2 (C3), 84.7 (C2), 72.8 (C1), 64.6 (q, *J* = 34.6, C4).

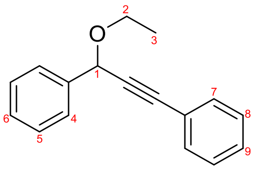

(3-Ethoxyprop-1-yne-1,3-diyl)dibenzene (**2a^{OEt}**):
^{1}H NMR (400 MHz, $CDCl_3$) δ (ppm): 7.58 (d, *J* = 7.6, 2H, H4), 7.47 (m, 2H, H7), 7.39 (t, *J* = 7.6, 2H, H5), 7.27-7.36 (m, 4H, H6, H8 and H9), 5.39 (s, 1H, H1), 3.80 (dq, *J* = 8.8, 7.1, 1H, H2), 3.63 (dq, *J* = 8.9, 7.1, 1H, H2), 1.29 (t, *J* = 7.0, 3H, H3).

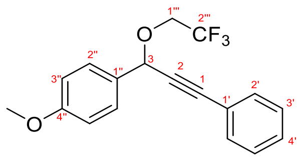

1-Methoxy-4-(3-phenyl-1-(2,2,2-trifluoroethoxy)prop-2-yn-1-yl)benzene (**2e$^{CF3-EtO}$**):
^{1}H NMR (600 MHz, $CDCl_3$) δ (ppm): 7.47-7.54 (m, 4H, H2', H2''), 7.31-7.39 (m, 3H, H3', H4'), 6.94 (d, *J* = 8.7, 2H, H3''), 5.61 (s, 1H, H3), 4.03 (dq, *J* = 12.0, 8.6, 1H, H1'''), 3.93 (dq, *J* =11.8, 8.9, 1H, H1'''), 3.83 (s, 3H, Me4'').
^{13}C NMR (150 MHz, $CDCl_3$) δ (ppm): 160.2 (C4''), 131.9 (C2'), 129.15 (C2''), 129.12 (C1''), 128.9 (C4'), 128.4 (C3'), 124.1 (q, *J* = 279.5, C2'''), 122.0 (C1'), 114.0 (C3''), 89.0 (C1), 84.9 (C2), 72.4 (C3), 64.4 (q, *J* = 34.4, C1'''), 55.4 (Me4'').

<u>Allenes 3:</u>

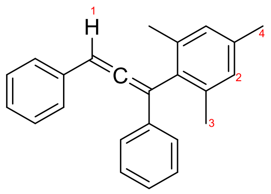

(1-Mesitylpropa-1,2-diene-1,3-diyl)dibenzene (**3a**):
^{1}H NMR (600 MHz, $CDCl_3$) δ (ppm): 7.38 (m, 2H), 7.16-7.34 (m, 8H), 6.94 (s, 2H, H2), 6.59 (s, 1H, H1), 2.31 (s, 3H, H4), 2.25 (s, 6H, H3).

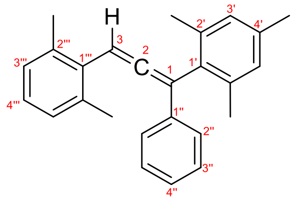

2-(3-(2,6-Dimethylphenyl)-1-phenylpropa-1,2-dien-1-yl)-1,3,5-trimethylbenzene (**3b**):
^{1}H NMR (600 MHz, $CDCl_3$) δ (ppm): 7.27 (dd, *J* = 7.6, 7.6, 2H, H3''), 7.23 (d, *J* = 8.3, 2H, H2''), 7.19 (t, *J* = 7.1, 1H, H4''), 6.99-7.06 (m, 3H, H3''' and H4'''), 6.92 (bs, 2H, H3'), 6.71 (s, 1H, H3), 2.35 (s, 6H, Me2'''), 2.31 (s, 3H, Me4'), 2.22 (bs, 3H, Me2'), 2.07 (bs, 3H, Me2').
^{13}C NMR (150 MHz, $CDCl_3$) δ (ppm): 205.8 (C2), 137.2 (broad, C2'), 137.1 (broad, C2'), 137.0 (C4'), 136.6 (C2'''), 136.3 (C1''), 131.7 (C1'), 131.4 (C1'''), 128.51 (C3'), 128.48 (C3''), 128.1 (C3'''), 126.74 (C4'''), 126.70 (C4''), 126.3 (C2''), 106.4 (C1), 92.2 (C3), 21.3 (Me2'''), 21.1 (Me4'), 20.3 (Me2').

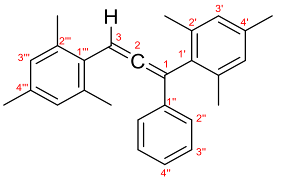

2,2'-(1-Phenylpropa-1,2-diene-1,3-diyl)bis(1,3,5-trimethylbenzene) (**3c**):
^{1}H NMR (600 MHz, $CDCl_3$) δ (ppm): 7.27 (dd, *J* = 7.5, 7.5. 2H, H3''), 7.22 (d, *J* = 7.2, 2H, H2''), 7.18 (dd, *J* = 7.1, 7.1, 1H, H4''), 6.92 (bs, 2H, H3'), 6.85 (s, 2H, H3'''), 6.70 (s, 1H, H3), 2.32 (s, 6H, Me2'''), 2.31 (s, 3H, Me4'), 2.26 (s, 3H, Me4'''), 2.21 (bs, 3H, Me2'), 2.07 (bs, 3H, Me2').
^{13}C NMR (150 MHz, $CDCl_3$) δ (ppm): 205.8 (C2), 137.2 (broad, C2'), 137.0 (broad, C2'), 136.9 (C4'), 136.5 (C2''' and C4'''), 136.3 (C1''), 131.9 (C1'), 129.0 (C3'''), 128.49 (C3'), 128.45 (C3''), 128.3 (C1'''), 126.6 (C4''), 126.3 (C2''), 106.2 (C1), 92.1 (C3), 21.2 (Me2'''), 21.1 (Me4'), 20.9 (Me4'''), 20.3 (Me2').

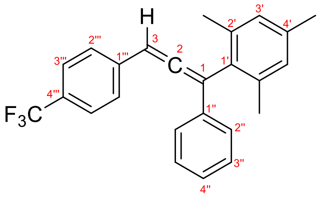

1,3,5-Trimethyl-2-(1-phenyl-3-(4-(trifluoromethyl)phenyl)propa-1,2-dien-1-yl)benzene (**3d**):
^{1}H NMR (600 MHz, $CDCl_3$) δ (ppm): 7.55 (d, *J* = 8.2, 2H, H3'''), 7.46 (d, *J* = 8.1, 2H, H2'''), 7.29 (m, 2H, H3''), 7.20-7.25 (m, 3H, H2'', H4''), 6.95 (s, 2H, H3'), 6.61 (s, 1H, H3), 2.32 (s, 3H, Me4'), 2.25 (s, 6H, Me2').
^{13}C NMR (150 MHz, $CDCl_3$) δ (ppm): 206.8 (C2), 138.2 (C1'''), 137.4 (C4'), 136.8 (C2'), 134.9 (C1''), 131.1 (C1'), 126.0 (q, *J* = 32.2, C4'''), 128.8 (C3''), 128.6 (C3'), 127.5 (C4''), 127.2 (C2'''), 126.3 (C2''), 125.7 (q, *J* = 3.7, C3'''), 124.2 (q, *J* = 271.8, $CF_3$4'''), 110.2 (C1), 96.1 (C3), 21.1 (Me4'), 20.5 (Me2').

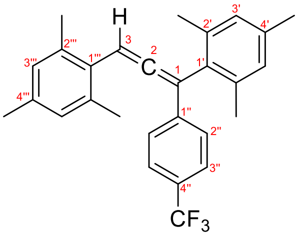

2,2'-(1-(4-(Trifluoromethyl)phenyl)propa-1,2-diene-1,3-diyl)bis(1,3,5-trimethylbenzene) (**3i**):
^{1}H NMR (600 MHz, $CDCl_3$) δ (ppm): 7.51 (d, *J* = 8.2, 2H, H3''), 7.32 (d, *J* = 8.2, 2H, H2''), 6.94 (bs, 2H, H3'), 6.86 (s, 2H, H3'''), 6.76 (s, 1H, H3), 2.32 (s, 3H, Me4'), 2.31 (s, 6H, Me2'''), 2.27 (s, 3H, Me4'''), 2.20 (bs, 3H, Me2'), 2.04 (bs, 3H, Me2').
^{13}C NMR (150 MHz, $CDCl_3$) δ (ppm): 206.5 (C2), 140.5 (C1''), 137.4 (C4'), 137.3 (broad, C2'), 136.9 (broad, C2'), 136.7 (C4'''), 136.5 (C2'''), 131.1 (C1'), 129.1 (C3'''), 128.7 (C3'), 128.6 (*J* = 32.3, C4''), 127.7 (C1'''), 126.4 (C2''), 125.4 (*J* = 3.7, C3''), 124.3 (*J* = 271.8, $CF_3$4''), 105.6 (C1), 92.5 (C3), 21.2 (Me2'''), 21.1 (Me4'), 20.9 (Me4'''), 20.3 (Me2').

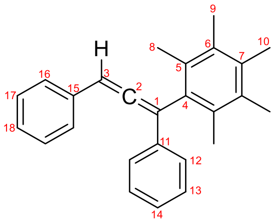

(1-(2,3,4,5,6-Pentamethylphenyl)propa-1,2-diene-1,3-diyl)dibenzene (**3j**):
^{1}H NMR (400 MHz, $CDCl_3$) δ (ppm): 7.36 (d, *J* =7.6, 2H, H16), 7.23-7.31 (m, 6H, H12, H13,H17), 7.19 (m, 2H, H14 and H18), 6.56 (s, 1H, H3), 2.31 (s, 3H), 2.27 (s, 3H), 2.25 (s, 3H), 2.22 (s, 6H).
^{13}C NMR (150 MHz, $CDCl_3$) δ (ppm): 206.1 (C2), 136.1 (C15), 134.51, 134.45, 133.2, 132.75, 132.74, 132.5, 132.3, 132.2, 128.63, 128.60, 127.2 (C16), 127.1 and 127.0 (C14 and C18), 126.4, 111.2 (C1), 96.9 (C3), 18.8, 17.8, 16.8, 16.7, 16.6.

<u>Indene 4b:</u>

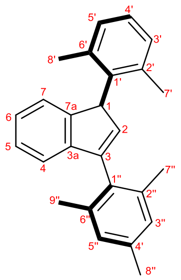

1-(2,6-Dimethylphenyl)-3-mesityl-1H-indene (**4b**):
^{1}H NMR (600 MHz, $CDCl_3$) δ (ppm): 7.23 (t, *J* = 7.3, H5), 7.14-7.215 (m, 3H, H6, H7, H3'), 7.09 (t, *J* = 7.5, 1H, H4'), 7.01 (d, *J* = 7.5, 1H, H4), 6.97 (bs, 2H, H3'', H5''), 6.89 (d, *J* = 7.4, 1H, H5'), 6.39 (d, *J* = 2, 1H, H2), 5.17 (s, 1H, H1), 2.66 (s, 3H, H7'), 2.35 (s, 3H, H8''), 2.18 (s, 3H, H9''), 2.11 (s, 3H, H7''), 1.65 (s, 3H, H8').
^{13}C NMR (150 MHz, $CDCl_3$) δ (ppm): 147.1 (C7a), 144.4 (C3a), 142.9 (C3), 138.2 (C6'), 137.6 (C2'), 137.2 (C6''), 137.0 (C2''), 136.9 (C4''), 135.8 (C2), 135.5 (C1'), 132.0 (C1''), 129.2 (C5'), 128.3 (C3' and C3''), 128.2 (C5''), 126.7 (C4'), 126.3 (C5), 125.2 (C6), 122.9 (C7), 120.6 (C4), 52.3 (C1), 21.9 (C7'), 21.1 (C8''), 20.7 (C9''), 20.4 (C7''), 19.0 (C8').
